# Melatonin enhances osteoblastogenesis of senescent bone marrow stromal cells through NSD2‐mediated chromatin remodelling

**DOI:** 10.1002/ctm2.746

**Published:** 2022-02-27

**Authors:** Ying Xie, Na Han, Feng Li, Lijuan Wang, Gerui Liu, Meilin Hu, Sheng Wang, Xuelei Wei, Jing Guo, Hongmei Jiang, Jingjing Wang, Xin Li, Yixuan Wang, Jingya Wang, Xiyun Bian, Zhongjiao Zhu, Hui Zhang, Chunhua Liu, Xiaozhi Liu, Zhiqiang Liu

**Affiliations:** ^1^ The Province and Ministry Co‐Sponsored Collaborative Innovation Center for Medical Epigenetics; Tianjin Key Laboratory of Cellular Homeostasis and Human Diseases; Department of Physiology and Pathophysiology, School of Basic Medical Science Tianjin Medical University Heping China; ^2^ Department of Central Laboratory and Institute of Clinical Molecular Biology Peking University People's Hospital; National Center for Trauma Medicine Beijing China; ^3^ Department of Orthopaedics Weifang People's Hospital Weifang China; ^4^ Central Laboratory; Linyi Key Laboratory of Tumor Biology Linyi People's Hospital Linyi China; ^5^ Department of Pharmacology, School of Basic Medical Science Tianjin Medical University Heping China; ^6^ Tianjin Medical University School of Stomatology Heping China; ^7^ Department of Emergency Tianjin Hospital Tianjin China; ^8^ Central Laboratory; Tianjin Key Laboratory of Epigenetics for Organ Development in Preterm Infants The Fifth Central Hospital of Tianjin Binhai Tianjin China; ^9^ Department of Orthopaedics Tengzhou Central People's Hospital Tenghzou China; ^10^ Department of Cardiology, Heart Centre; Ministry of Education Key Laboratory of Child Development and Disorders National Clinical Research Center for Child Health and Disorders; Chongqing Key Laboratory of Pediatrics; China International Science and Technology Cooperation Base of Child Development and Disorders Children's Hospital of Chongqing Medical University Chongqing China; ^11^ Department of Physiology Shandong First Medical University (Shandong Academy of Medical Sciences) Jinan Shandong China

**Keywords:** bone marrow stromal cells, melatonin, NSD2, osteoporosis, senescence

## Abstract

**Background:**

Aging‐associated osteoporosis is frequently seen in the elderly in clinic, but efficient managements are limited because of unclear nosogenesis. The current study aims to investigate the role of melatonin on senescent bone marrow stromal cells (BMSCs) and the underlying regulating mechanism.

**Methods:**

Melatonin levels were tested by ELISA. Gene expression profiles were performed by RNA‐sequencing, enrichment of H3K36me2 on gene promoters was analyzed by Chromatin Immunoprecipitation Sequencing (ChIP‐seq), and chromatin accessibility was determined by Assay for Transposase‐Accessible Chromatin with high‐throughput sequencing (ATAC‐seq). Osteogenesis of BMSCs in vitro was measured by Alizarin Red and Alkaline Phosphatase staining, and in vivo effects of melatonin was assessed by histological staining and micro computed tomography (micro‐CT) scan. Correlation of NSD2 expression and severity of senile osteoporosis patients were analyzed by Pearson correlation.

**Results:**

Melatonin levels were decreased during aging in human bone marrow, accompanied by downregulation of the histone methyltransferase nuclear receptor binding SET domain protein 2 (NSD2) expression in the senescent BMSCs. Melatonin stimulated the expression of NSD2 through MT1/2‐mediated signaling pathways, resulting in the rebalancing of H3K36me2 and H3K27me3 modifications to increase chromatin accessibility of the osteogenic genes, runt‐related transcription factor 2 (RUNX2) and bone gamma‐carboxyglutamate protein (BGLAP). Melatonin promoted osteogenesis of BMSCs in vitro, and alleviates osteoporosis progression in the aging mice. In clinic, severity of senile osteoporosis (SOP) was negatively correlated with melatonin level in bone marrow, as well as NSD2 expression in BMSCs. Similarly, melatonin remarkably enhanced osteogenic differentiation of BMSCs derived from SOP patients in vitro.

**Conclusions:**

Collectively, our study dissects previously unreported mechanistic insights into the epigenetic regulating machinery of melatonin in meliorating osteogenic differentiation of senescent BMSC, and provides evidence for application of melatonin in preventing aging‐associated bone loss.

## Graphical abstract

1. Melatonin enhances osteoblastogenesis of senescent BMSCs by upregulating the expression of the histone methyltransferase NSD2.
2. NSD2 rebalances the distribution of H3K36me2 and H3K27me3 and faciliates chromatin accessibility on promoters of osteogenic genes *RUNX2* and *BGLAP*.
3. Our study potentiates the application of melatonin as a therapeutic agent for the prevention of aging‐associated osteoporosis in clinic.

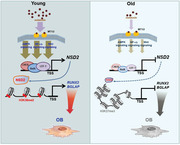

## 1 INTRODUCTION

Large numbers of elderly people are suffering from osteoporosis in the clinic, a debilitating chronic disease characterized by the loss of bone mass and strength, resulting in fragile bones and fracture.^1^ Melatonin is secreted predominantly by the pineal gland under controls of the suprachiasmatic nucleus and circadian rhythms.^2^, ^3^ Accumulating evidences have also indicated that melatonin is involved in bone remolding, osteoporosis, osseointegration of dental implants, and dentine formation.^4^, ^5^ The aging‐related reduction of melatonin levels has been associated with bone loss and osteoporosis during aging.^6^ Therefore, serum melatonin levels might be utilized as a biomarker for the early monitoring and prevention of osteoporosis, and a better understanding of the functional machinery of melatonin will benefit the application of melatonin in alleviating the aging‐related progression of osteoporosis.^6^

Bone marrow stromal cells (BMSCs) are multipotent stem cells that can differentiate into a variety of osteogenic, chondrogenic, adipogenic, or myogenic lineages.^7^ Melatonin can modulate multiple signals to drive the commitment and differentiation of BMSCs into osteoblasts.^8^, ^9^ Increased oxidative stress and cell injury with aging are causal factors of reduced osteogenesis by BMSCs.^10^ Studies have confirmed that melatonin can promote osteoblast‐like cell proliferation, enhance the expression of type I collagen and bone marker proteins and facilitate the formation of a mineralized matrix.^11^, ^12^ A study also suggested that melatonin exerts suppressive effects on osteoclasts via the up‐regulation of calcitonin secretion by osteocytes.^13^ Mechanistically, through binding to the MT2 receptor, melatonin elevates the gene expression of bone morphogenetic protein 2 (*BMP2*), *BMP6*, alkaline phosphatase (*ALP*), osteocalcin and osteoprotegerin to favour osteogenesis and simultaneously suppresses the receptor activator of NF‐κB ligand pathway to attenuate osteolysis.^6^ However, despite these known phenotypes and functions of melatonin on osteoblastogenesis, the substantial molecular regulatory mechanisms, especially the epigenetic machinery, are still not well elucidated.

Osteoblasts are bone‐forming cells derived from BMSCs, and the stemness and differentiation properties of BMSCs have been shown to decline with cellular senescence.^14^ Nevertheless, an in‐depth understanding of the mechanisms involved in cellular senescence remains elusive due to the highly intrinsic heterogeneity and complicated genetic or epigenetic regulatory processes in BMSCs. Melatonin is an effective agent for the alleviation of apoptotic factors to protect BMSCs from cell injury.^15^ A series of studies conducted by Lee and colleagues have demonstrated that melatonin treatment enhances kidney‐derived MSC proliferation and prevents cell senescence.^15^, ^16^, ^17^ A study showed that melatonin could restore the osteoporosis‐impaired osteogenic potential of BMSCs by preserving Sirtuin 1‐mediated intracellular antioxidation.^18^ Moreover, incubation of BMSCs with melatonin predominantly enhances the expression of BCL2 but decreases the expression of BAX, to protect BMSCs from apoptosis.^19^ Despite these findings, it is unknown whether the beneficial effect of melatonin on maintaining BMSC regeneration is based on an aging‐associated mechanism.

In the present study, we measured melatonin level in the aging human bone marrow and mouse blood plasma and profiled genes expressions of human and mouse senescent BMSCs to screen differentially expressed genes related to aging and discovered the expression of a histone methyltransferase NSD2, which catalyses H3K36me2 and activates gene expression, was closely correlated with decreased melatonin level during aging. We investigated the epigenetic regulating machinery of NSD2 on osteogenesis of the senescent BMSCs through in vitro and in vivo experiments and assessed the significance of melatonin in alleviating SOP in aging mouse model and in promoting osteogenesis of clinical samples.

## 2 METHODS

### 2.1 Ethic approval

The ethic committee of Tianjin Medical University has approved this study (number: TMUhMEC2018014), and protocols were conformed to the ethical guidelines of the World Medical Association Declaration of Helsinki. All participating individuals have signed informed consent prior to participation in the study. The Committee on Animal Research and Ethics of Tianjin Medical University and the Animal Experiments Ethics Committee of the Fifth Central Hospital of Tianjin (number: TJWZX2018047) both have approved animal studies in our study (number: TMUaMEC2018001). All protocols are complied with the Guidelines for Ethical Conduct in the Care and Use of Nonhuman Animals in Research.

### 2.2 Collection of bone marrow samples and isolation of BMSCs

Donor's samples were all obtained at 8:00∼11:00 in the morning, and supernatants were collected to determine melatonin levels. BMSCs were collected from femur, vertebra, or anterior superior iliac spine. For BMSCs isolation from young (*n* = 12, 17–44 years old, median = 25, 10 males and 2 females) and elderly healthy donors (*n* = 12, 65–82 years old, median = 68.5, 10 males and 2 females) or osteoporosis patients (*n* = 12, 74–90 years old, median = 79, all male), 3–5 ml of bone marrow biopsies were diluted up to 10 ml with complete culture media,^20^ and cells were then fractionated by density gradient method. For BMSCs isolation from femur of aborted human fetuses (*n* = 12, 16–22 weeks, median = 18.5, 7 males and five females) and C57BL/6 mouse, bone marrow contents were flushed into 10‐cm dish with the complete culture media for culture and purification after removing non‐adherent cells. Thereafter, media were changed every 2 days. BMSCs derived from donors from different ages have been cultured for the same number of passages and maintained at the same percentage of confluence before passaging. When BMSCs reached 85%–95% confluence, cells were trypsinized and counted for further experiments. Identity of BMSC properties was validated by flow cytometry using CD14, CD45, CD90 and CD105. BMSCs from passages 3–6 were applied to further experiments.

### 2.3 Virus packaging and infection

Transient transfections and virus packaging were performed as previously described.^21^ Briefly, viral particles were produced in HEK293T cells transfected with 4 μg PMD_2_G, 6 μg psPAX_2_ and 8 μg lentiviral expressing vectors encoding target genes plasmids (Addgene, Watertown, MA, USA) in a 10‐cm dish. pCMV3‐C‐HA‐NSD2 expressing NSD2 and pLKO.1 vector encoding shRNAs targeting *NSD2* gene were used. Viral particles were harvested 48 h after transfection and concentrated to 100 × volume by Poly (ethylene glycol) 8000 (Sigma‐Aldrich, St. Louis, MS, USA).

For viral infection, 2 × 10^5^ BMSCs in 1‐ml new media were added with 50‐μl concentrated viral in presence of 8‐μg/ml polybrene, and then spin at 1800 × rpm for 45 min at room temperature. Medium was changed 12 h after spinfection, and cells were cultured for 2 days until further designed experiments.

### 2.4 Senescence β‐galactosidase staining

BMSCs were cultured in six‐well plate for indicated time, then culture medium was removed and washed by 1 × Phosphate Buffered Saline (PBS). After cells were fixed with 1 mL of fixative solution (10% formaldehyde, 1% glutaraldehyde in DPBS) for 15 min and washed three times by PBS, staining solution (1 mL) was added to incubate cells at 37°C for hours until chromogenic reaction achieved.

### 2.5 Osteogenesis induction in vitro

Cells were normalized at the beginning to ensure that BMSCs reached the consistent confluence. After reaching 80% confluence, culture medium was changed to osteogenic medium in the presence or absence of 1 μmol/L melatonin for 7 days (immature osteoblasts stage) and 14 days (mature osteoblasts stage) with a medium change every 3 days. The osteogenic differentiation medium of hBMSCs was composed of L‐ascorbic acid (50 μg/mL), dexamethasone (.1 μmol/L) and β‐glycerolphosphate (10 mmol/L) in Dulbecco's Modified Eagle Medium (DMEM) complete media, and mBMSCs were in Alpha Minimum Essential Media (Alpha MEM) (dexamethasone+) with L‐ascorbic acid (.1 mmol/L), β‐glycerophosphate (2 mmol/L). Osteogenic differentiation efficiency was detected by Alizarin Red S staining or ALP assay as previously described.^22^, ^23^

### 2.6 Enzyme immunoassay

For serum P1NP measurements, blood samples were collected from 18‐month‐old mice treated with vehicle control or melatonin at 2:00 pm. P1NP was measured using commercial kits (AC‐33F1 for P1NP, Immunodiagnostic Systems) according to the manufacturer's instructions.

Melatonin level in bone marrow plasma was measured with human MT (Melatonin) Enzyme Linked Immunosorbent Assay (ELISA) Kit (Elabscience, Cat: E‐EL‐H2016c, Wuhan, China) accordingly. Briefly, bone marrow plasma from young (*n* = 15, aging 17–45 years, median = 30, 11 males and 4 females) and elderly donors (*n* = 24, aging 56–84 years, median = 65.5, 15 males and 9 females) were diluted at 1:5, filled into a 96‐well plate with primary antibody, and incubated at 37°C for 45 min. Then, secondary antibodies were diluted (1:500) and added into plates at 37°C for 30 min. Finally, signals were developed for detection after substrate was added. The optical density (value) of each well was determined at once with wavelength 450 nm.

### 2.7 Quantitative Polymerase Chain Reaction (qPCR) and Western blotting

Procedures of qPCR and Western blotting have been detailed in our previous study.^21^ The formula 2^–ΔΔCt^ was used to calculate the fold change in gene expression, and the average ΔCt value of three biological experiments was recorded. Primers and antibodies used in this study were listed in the supplementary resources. At least three independent experiments were used for statistical analysis.

### 2.8 Chromatin‐immunoprecipitation assays

In this study, 20 million BMSCs were used for chromatin‐immunoprecipitation (ChIP) assay, and protocol has been detailed in our previous report.^21^ Deoxyribo nucleic acid (DNA) fragments were precipitated and analysed by qPCR or high‐throughput sequencing (Novogene Co., Ltd.). Antibodies for WB and ChIP assays were all listed in the supplementary resources.

### 2.9 RNA‐sequencing and analysis

TRIzol (Invitrogen) was used to isolate total ribonucleic acids (RNAs) from BMSCs. cDNA libraries were amplified and sequenced using the BGISEQ‐500 platform (BGI Group, Shenzhen, P.R. China). FastQC (version 0.11.8) was used to check raw sequencing reads. Adapter trimming and low‐quality filtering was analysed by Cutadapt (v2.0) to get the clean data. Homo sapiens (hg38, GRCh38) from GENCODE were used to analyse genome sequence and gene annotation. The gene expression was quantified by featureCounts (v1.6.0), and differential expressed genes (DEGs) between treatment groups were created by DESeq2 (R version 3.3.2). Cutoff value of fold change (fc) ≥1.5 and adjust *p*‐value ≤.05 was used.

### 2.10 Assay for transposase‐accessible chromatin with high‐throughput sequencing assay

Briefly, after washed by cold PBS, 5 × 10^4^ cells were lysed in lysis buffer (10 mM Tris‐Cl, pH 7.4, 10 mM NaCl, 3 mM MgCl2 and .1% IGEPAL CA‐630). Immediately afterwards, nuclei were spun down and resuspended in the transposase reaction mix for 30 min at 37°C. Followed by transposition, samples were purified using a DNA Clean and Concentrator‐5 kit (ZYMO RESEARCH, California, USA). Library fragments were amplified using Q5 Hot Start High‐Fidelity DNA Polymerase using PCR primers N5 and N7 (TruePrep Index Kit V2 for Illumina, Vazyme, TD202). After 13 cycles of amplification, the PCR reaction was purified by VAHTS DNA Clean Beads (Vazyme, N411‐01). Purified DNA fragments were analysed by high‐throughput sequencing (Novogene, Beijing, China). The UCSC hg38 reference genome with BOWTIE2 was referred to align the assay for transposase‐accessible chromatin with high‐throughput sequencing (ATAC‐seq) reads. ATAC‐seq peaks were called out and annotated through MACS2, and known transcription factor binding motifs were further analysed in the ATAC‐seq peaks by HOMER.

### 2.11 Animal experiment and bone morphology in vivo

Aging C57 mice (18 months old) and adult C57 mice (2 months old) were blindly randomized to mock group and melatonin treatment group and treated with vehicle or 10 mg/kg melatonin respectively by subcutaneous injection for 10 weeks, twice a week. To assess the in vivo bone morphology, high‐resolution X‐ray microtomography was carried out on mice femur using the SkyScan 1276 microtomograph (Bruker microCT, Kontich, Belgium). Femurs dissected from mice were loaded into special tubes and scanned with 7‐μm resolution. The scans were integrated into 3D voxel images, and a region of interest (a 1 mm length starting from .5 mm proximal to the growth plate) that consisted of 140 slices was chosen for analysis. CtAn software (release 2.5, Skyscan) was used to construct 3D models, and 3D measurements were obtained with the CtAn software (release 2.5, Skyscan).

### 2.12 Histology of mouse bone tissues

Mouse femoral tissues were fixed with 4% paraformaldehyde in PBS, decalcified and embedded in paraffin completely. Paraffin‐embedded tissues were cut into 3‐μm thick sections. Bone tissue slides were undergone hematoxylin and eosin (H&E) staining, toluidine blue staining, or immunostained with anti‐mouse Leptin Receptor, anti‐mouse NSD2, and anti‐mouse RUNX2 antibodies. The sections were observed under a microscope Olympus FV1000 (Tokyo, Japan).

### 2.13 Statistical analysis

Data are shown as mean ± s.e.m for at least three biological experiments. Differences between different treatments were analysed using two‐tailed Student's *t*‐test or two‐way Analysis of Variance (ANOVA). For post hoc analysis, the least significant difference (LSD) test is used when treatments ≤ 2, and the Tukey's honest significant difference test is used when treatments are ≥ 3. Pearson correlation measuring correlations were all determined by GraphPad Prism 9.0. A *p* value <.05 was considered significantly difference. ***p < .001; ****p < .0001, respectively if applicable.

## 3 RESULTS

### 3.1 Melatonin levels decrease with aging in human bone marrow and mouse blood

**FIGURE 1:**
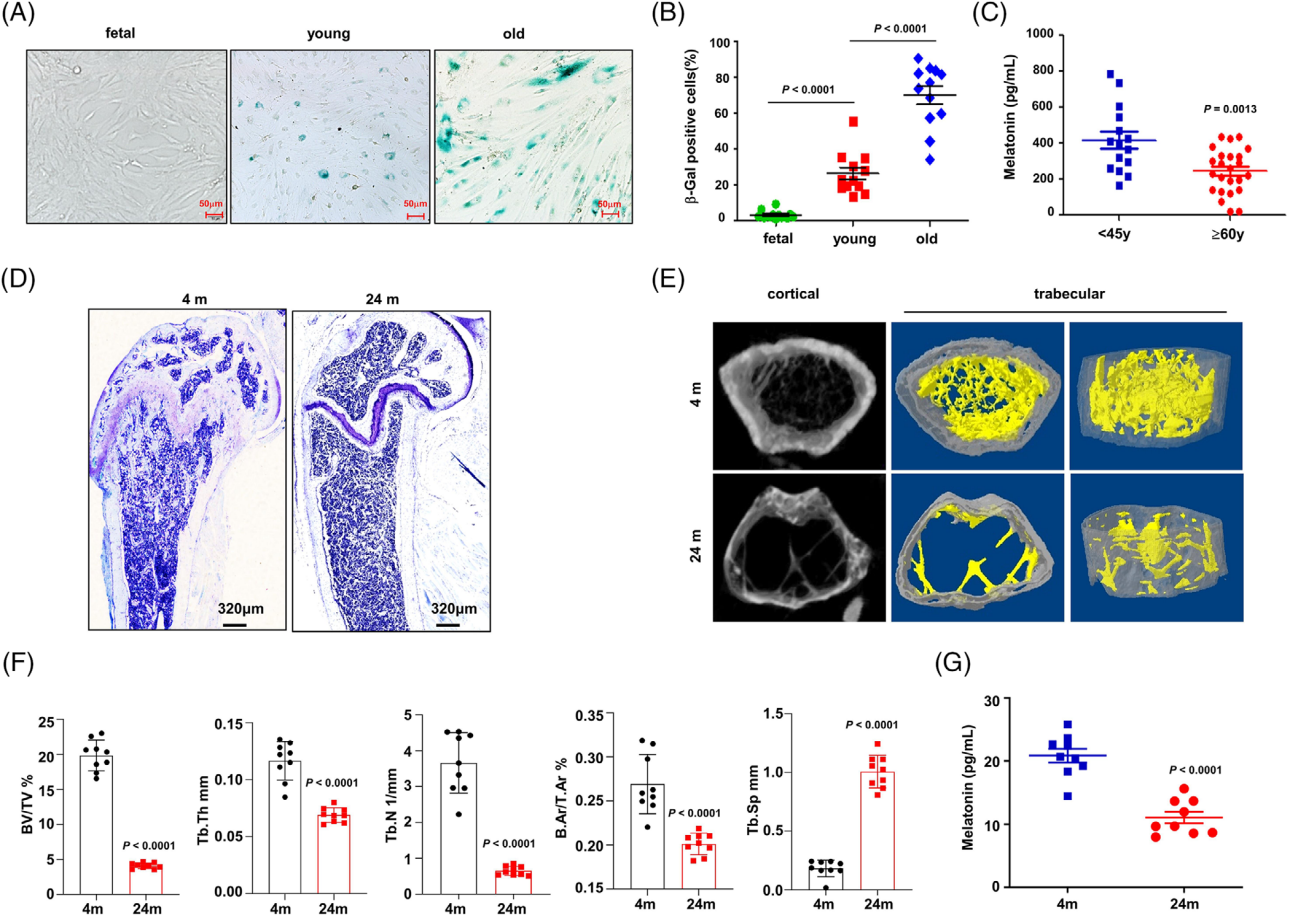
Melatonin level declines with aging in human bone marrow and mouse plasma. (A) Representative images of lysosomal β‐galactosidase staining in bone marrow stromal cells (BMSCs) from donors with different ages. Scale bar, 50 μm. (B) Quantification of the percentage of β‐galactosidase positive BMSCs in three groups (*n* = 12). Nine random vision fields with 200 × magnification were analysed. (C) Melatonin levels in the bone marrow plasma of donors under 45 years old (*n* = 15) or over 60 years old (*n* = 24) measured by ELISA. (D) Toluidine Blue O staining of the trabecular bone near the distal femoral metaphyseal region from adult (4 months) and aged mice (24 months). (E) Representative three‐dimensional trabecular architecture examined by a micro‐CT scan of the trabecular bone near the distal femoral metaphyseal region from adult and aged mice. Region of interest (ROI), 1 mm length starting from .5 mm proximal to the growth plate consisting of 140 slices. (F) Quantifications of the trabecular bone volume/tissue volume (BV/TV), trabecular thickness (Tb.Th), trabecular number (Tb.N), trabecular separation (Tb.Sp) and mean cortical area fraction (B.Ar/T.Ar) (*n* = 9 mice per group). (G) Melatonin level in the peripheral blood of adult and aged mice. *n* = 9 mice per group. Data are mean ± s.e.m. *p* values are determined by unpaired two‐sided *t*‐tests with Welch's correction

Because melatonin is an important endocrine hormone regulating osteogenesis and bone homeostasis, and previous studies have reported the declining melatonin level in human peripheral blood,^24^, ^25^ thus we firstly confirmed the melatonin level in human bone marrow plasma from donors of different ages, as well as in mouse blood plasma. The aging phenotypes of human BMSCs were evidenced by increasing positive rate of beta‐galactosidase staining (Figure 1A,B), based on our successful isolation and purification of BMSCs (Figure S1A). The amount of melatonin in bone marrow decreased in an age‐associated manner, as the average level of melatonin in donors aged under 45 years was approximately 400 pg/ml, but dropped to less than 250 pg/ml in donors over 60 years old (Figure 1C). Meanwhile, we assessed the expression of melatonin receptors, *MT1* and *MT2*, in the BMSCs of different groups but did not find significant changes (Figure S1B). At the same time, using a 24 months old aging mouse model and 4 months adult mice as control, in which the aged mice were all presented bone mass loss (Figure 1D), as evidenced by reduced trabecular bone volume fraction (Bone Volume/Total Volume, BV/TV), number (trabecula numbers [Tb.N]), thickness (Tb.Th), mean cortical area fraction (B.Ar/T.Ar), and by expanded separation (trabecula separation [Tb.Sp]) in trabecular of the femur metaphysis (Figure 1E,F), we also confirmed that melatonin level was significantly declined in the aged mice compared to the adult controls (Figure 1G). Seemingly, melatonin levels in bone marrow have a close link with aging‐associated osteoporosis.

### 3.2 NSD2 expression is suppressed in the senescent BMSCs

**FIGURE 2:**
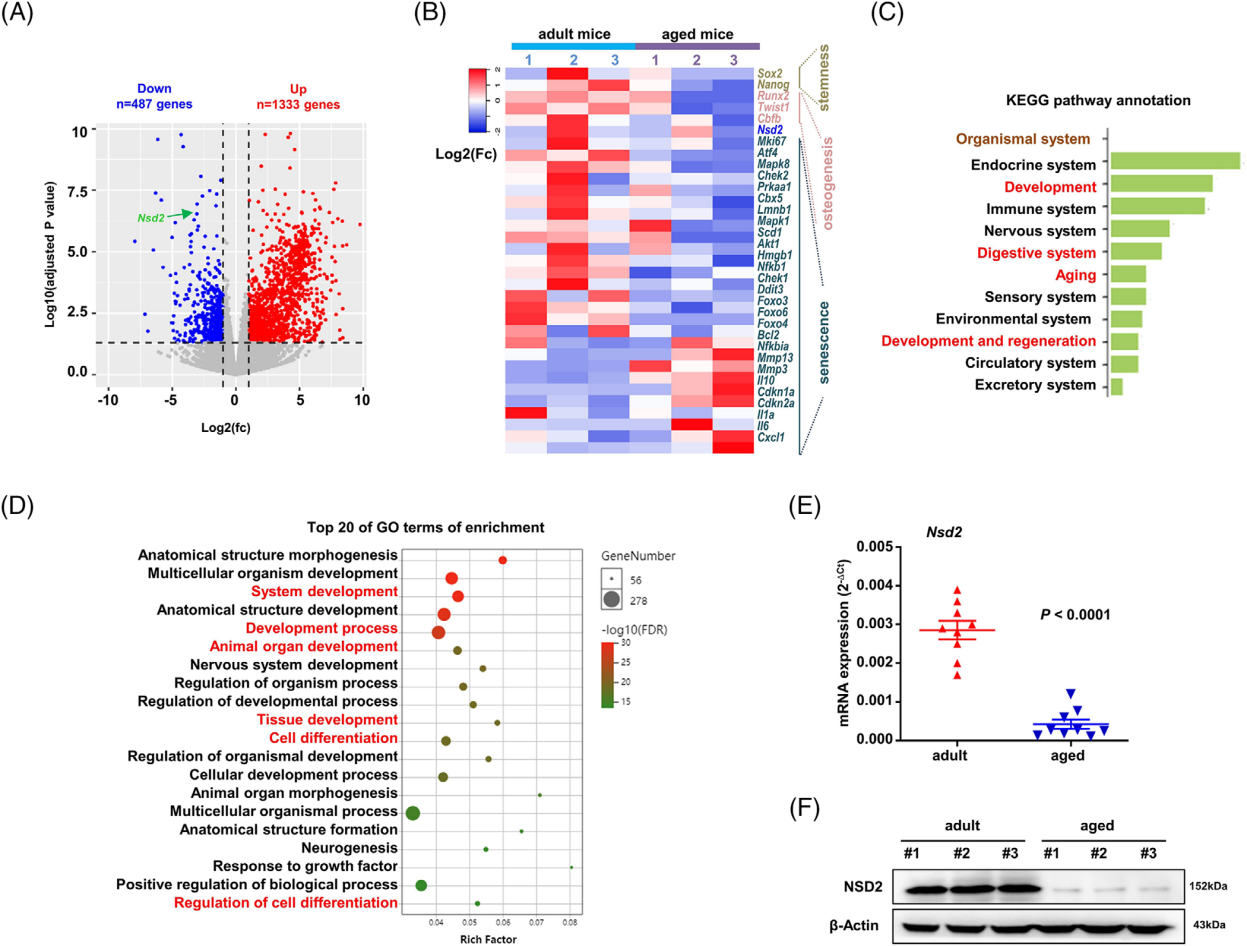
Gene expression profiling identifies NSD2 as marker of senescent bone marrow stromal cells (BMSCs). (A) Volcano plot showing differently expressed genes in BMSCs from aged mice compared to the young adult as control. Dashed lines show differential expression cutoffs (log 2 [fc] at 1% FDR). (B) Heat map of differently expressed key genes of stemness, senescence and osteogenesis in BMSCs from aged and adult mice. (C) Kyoto Encyclopedia of Genes and Genomes (KEGG) pathway annotation of differential expressed genes (DEGs) involved in organismal system. (D) Gene Ontology (GO) analysis of top 20 enriched terms of the DEGs. (E) qPCR analysis of *Nsd2* expression in BMSCs derived from adult and aged mice (*n* = 9 mice per group). (F) Western blotting assay of the NSD2 level in BMSCs isolated from adult and aged mice. Data are mean ± s.e.m. *p* values are determined by unpaired two‐sided *t*‐tests with Welch's correction

To screen for differentially expressed genes related to aged mice, we compared the differently expressed genes (GEGs) of BMSCs derived from adult and aged mice, using RNA‐sequencing analysis. RNA‐seq analysis identified 1820 differentially expressed genes (DEGs), among which 1333 genes were up‐regulated and 487 genes were down‐regulated in the aged group compared to the adult group (Figure 2A, Table S1). Some key down‐regulated genes were classified into stemness‐related genes, such like *Sox2, Nanog;* osteogenesis‐related genes such like *Runx2*, *Twist1*, and *Cbfb*; and senescence‐related up‐regulated genes such like *IL1a, IL6, Cdkn1a*, and *Cxcl1*; importantly, we found expression of NSD2, a histone methyltransferase catalysing H3K36me2, was remarkably down‐regulated (Figure 2B). The KEGG pathway annotation showed the DEGs were involved in development, regeneration and aging (Figure 2C), and the GO enrichment analysis for the up‐regulated genes indicated the DEGs were closely related to system development and cell differentiation (Figure 2D).

Since NSD2‐mediated H3K36me2 plays very important in regulating gene transcription,^26^ and H3K36me2 is critical to maintain embryonic stem cells property,^26^ we next sought to investigate whether melatonin regulates BMSC senescence through NSD2. When the messenger ribonucleic acid (mRNA) level and protein levels of NSD2 were evaluated in BMSCs from the adult and aged mice, we confirmed a significant inhibition of *Nsd2* expression in the aged BMSCs (Figure 2E), and Western blot analysis further revealed that NSD2 protein level was significantly decreased in the senescent BMSCs (Figure 2F). In addition, we also isolated and purified BMSCs from mouse bone marrow (Figure S1C). Real‐time PCR confirmed that *NSD2* was maintained at a relatively high level in MSCs from fetal and young adult donors, while its expression was dramatically reduced in senescent BMSCs from elderly donors (Figure S1D). Western blot analysis further revealed that NSD2 protein level was significantly decreased in the senescent BMSCs (Figure S1E). Taken together, these data indicated that NSD2 may be an important factor in aging‐associated senescence of BMSCs.

### 3.3 Osteogenic potential of senescent BMSCs declines in parallel with NSD2 down‐regulation

**FIGURE 3:**
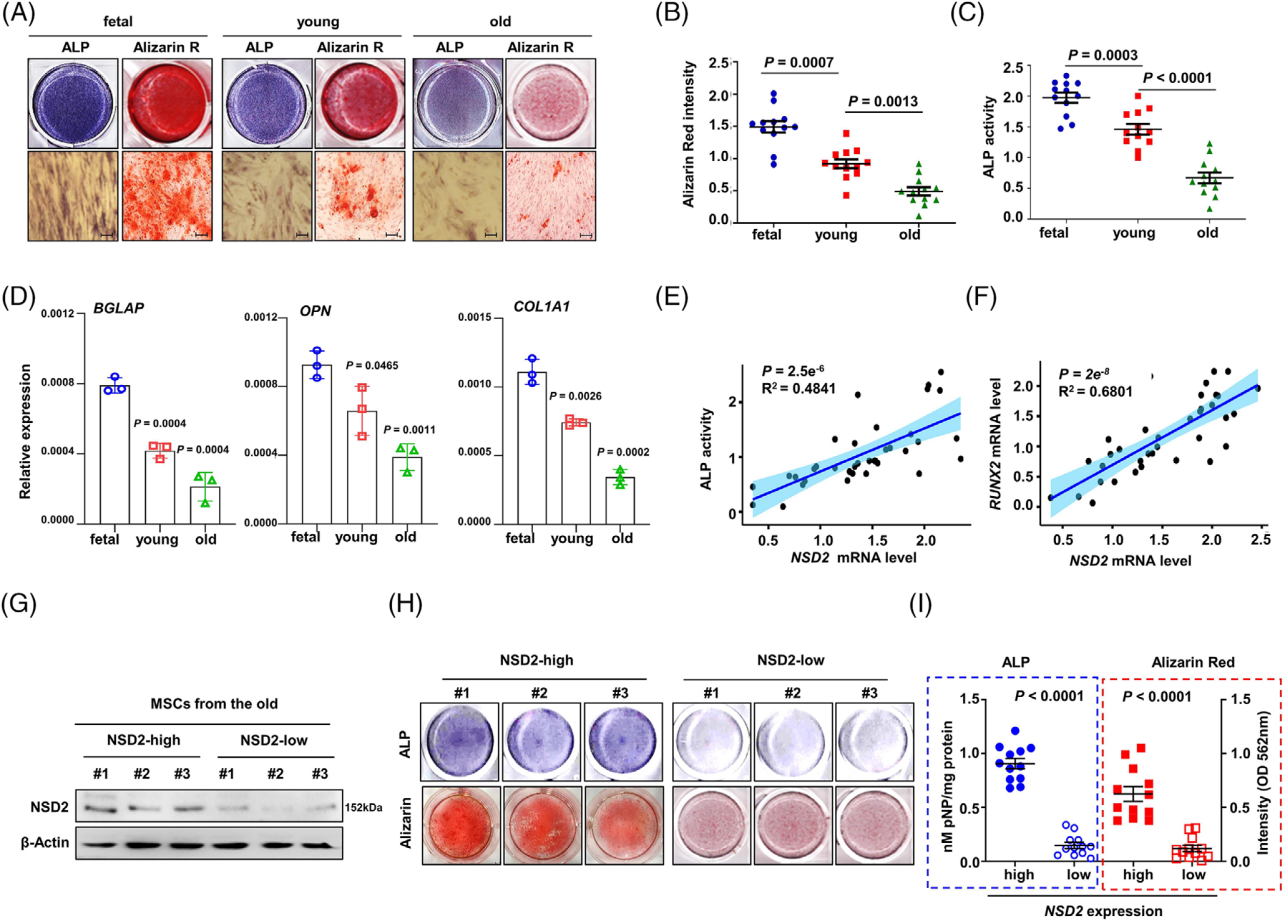
Osteogenic potential of senescent bone marrow stromal cells (BMSCs) correlates with NSD2 expression. (A) Representative images of alkaline phosphatase assay (ALP) staining and Alizarin Red S assay for BMSCs from fetal, young or old donors cultured with osteogenic media for 14 days and quantification of Alizarin Red staining (B) and ALP staining (C) (*n* = 12 samples per group). Scale bar, 100 μm. (D) qPCR assay for expressions of osteogenic genes *BGLAP, OPN, COL1A1* in different aged BMSCs after induced for 14 days. (E) Correlation of ALP activity and *NSD2* expression in BMSCs of different groups (*n* = 36). (F) Correlation of *RUNX2* and *NSD2* expression in BMSCs of different groups (*n* = 36). (G) Representative Western blotting analysis of NSD2 protein level in the senescent BMSCs. (H) Representative images of ALP staining and Alizarin Red S assay in the senescent BMSCs with different NSD2 levels. (I) Quantification of ALP staining and Alizarin Red S assay in human senescent BMSCs with different NSD2 levels (*n* = 12 samples per group). Data are mean ± s.e.m. *p* values are determined by unpaired two‐sided *t*‐tests with Welch's correction

To assess the osteogenic differentiation potential of human and mouse BMSCs isolated from the different age groups, we cultured them in osteoblast differentiation medium for 14 days. We found that senescence led to the impaired osteogenic potential of human BMSCs, as indicated by decreased ALP activity and mineralization staining (Figure 3A). Quantification of ALP activity and the mineralized nodules also confirmed the gradual decline of osteogenic potential with the senescence of human BMSCs (Figure 3B,C). Meanwhile, the expression of osteogenic marker genes, including *BGLAP*, osteopontin (*OPN*) and type I collagen alpha 1 (*COL1A1*), was reduced in the senescent BMSCs (Figure 3D). Remarkably, association analysis indicated a strong correlation between ALP levels and *NSD2* expression (Figure 3E), as well as between *RUNX2* and *NSD2* expression (Figure 3F). Interestingly, BMSCs from elderly donors exhibited higher ALP activity and mineralized nodule formation after the induction of osteogenesis if they possessed higher levels of NSD2 expression (Figure 3G–I). When comparing the old and young groups, we used the method of matching age and NSD2 expression at the same time to exclude diverse individual backgrounds and ensure the reliability of experimental results. Akin to human samples, BMSCs from different age mouse had different NSD2 levels (Figure S2A), and BMSCs with higher NSD2 expression engendered stronger ALP activity and more mineralization after ontogenetic induction (Figure S2B–D), which was also reflected by significantly high expressions of osteogenic marker genes (Figure S2E). These data strongly indicate that there was a significant correlation between NSD2 and osteogenesis in BMSCs.

### 3.4 Melatonin stimulates NSD2 expression in senescent BMSCs

**FIGURE 4:**
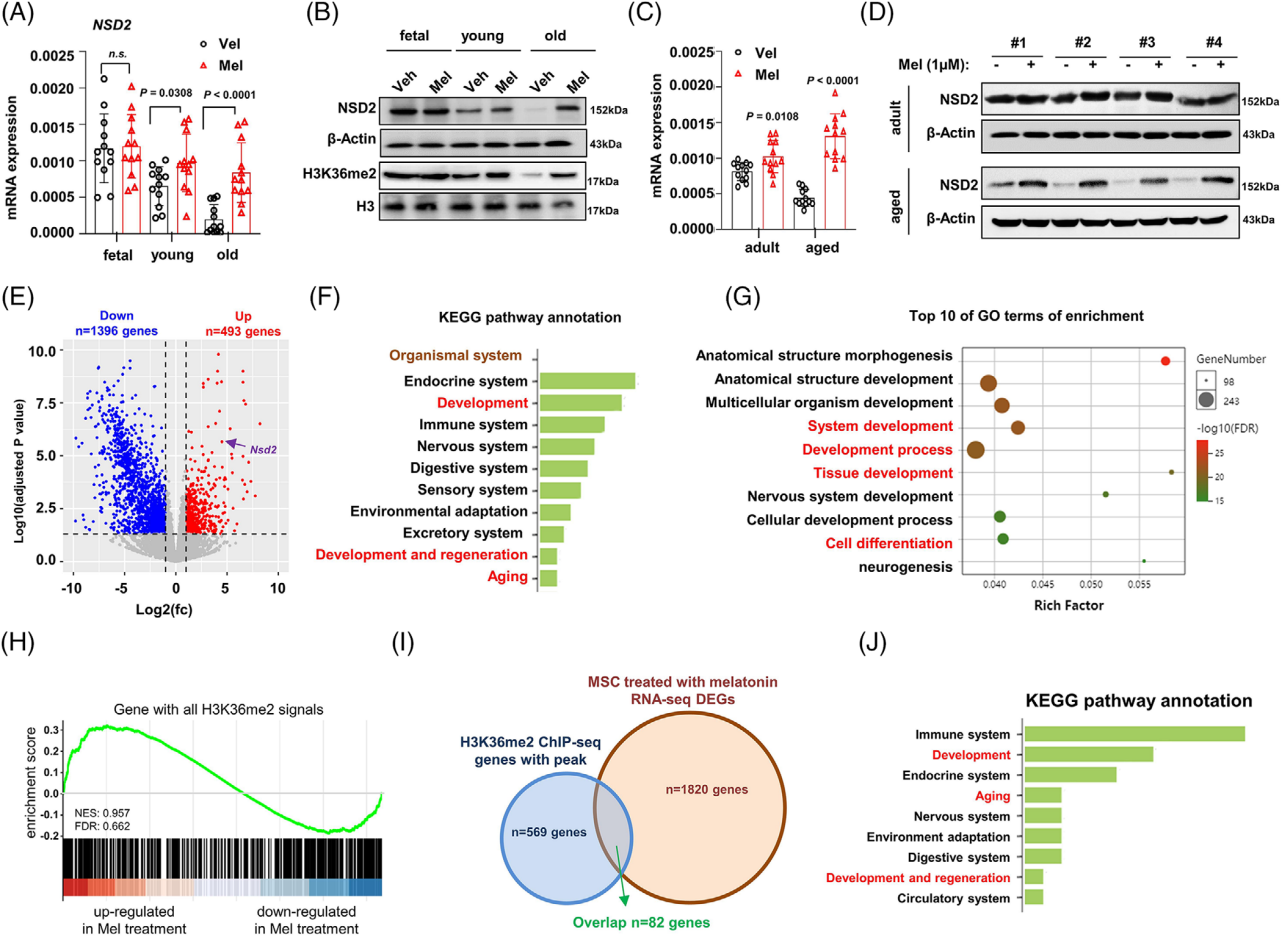
Melatonin stimulates NSD2 expression in senescent bone marrow stromal cells (BMSCs). (A) qPCR analysis of *NSD2* expression in human BMSCs from donors of different ages treated with 1 μM melatonin for 24 h (n = 12 samples per group). n.s., no significance. (B) Western blotting analysis of NSD2 and H3K36me2 levels in human BMSCs from donors of different ages and treated with 1 μM melatonin for 24 h. (C) qPCR analysis of *Nsd2* expression in mouse BMSCs from adult and aged groups and treated with 1 μM melatonin for 24 h (*n* = 12 samples per group). (D) NSD2 level in mouse BMSCs from adult and aged groups and treated with 1 μM melatonin for 24 h. (E) Volcano plot of differentially expressed genes analysed from bulk RNA‐sequencing in the senescent mouse BMSCs treated with vehicle or 1 μM melatonin for 24 h. Blue, down‐regulated genes; red, up‐regulated genes; light gray, statistically non‐significance genes (*n* = 3 mice per group). (F) Kyoto Encyclopedia of Genes and Genomes (KEGG) pathway annotation of differential expressed genes (DEGs) involved in organismal system. (G) Gene Ontology (GO) analysis of top 10 enriched terms of the DEGs. (H) The gene set enrichment analysis (GSEA) showing up‐ and down‐regulated genes enriched by H3K36me2 antibody in mouse senscent BMSCs treated with 1 μM melatonin for 24 h by chromatin‐immunoprecipitation (ChIP)‐sequation (*n* = 3 biological experiemtns). (I) Venn diagram showing the number of overlaps between genes bound by H3K36me2 ChIP‐seq assay and DEGs upon melatonin treatment by RNA‐seq assay. (J) Kyoto Encyclopedia of Genes and Genomes (KEGG) pathway annotation of overlapped genes bound by H3K36me2 ChIP‐seq assay and DEGs upon melatonin treatment by RNA‐seq assay. Data are mean ± s.e.m. *p* values are determined by paired two‐sided *t*‐tests with Welch's correction

Previous studies have discovered that melatonin has stimulative effect on osteogenic differentiation of BMSCs.^27^ To clarify whether melatonin promotes osteogenic differentiation through NSD2, we treated cultured BMSCs from different aged donors with exogenous melatonin (1 μM) and then examined the NSD2 expression. Melatonin failed to induce remarkable change of NSD2 at mRNA level in the fetal BMSCs, but changes in the young BMSCs were significant (*p* < .05), and the differences in the old BMSCs were even distinct (*p* < .001) (Figure 4A). Consistently, lower NSD2 protein level was correlated with lower global H3K36me2 level in aging BMSCs, and melatonin treatment restored the NSD2 and H3K36me2 levels only in the aged BMSCs, indicating sensitivity to melatonin of BMSCs was age‐related (Figure 4B, Figure S3A). Similarly, melatonin treatment elicited much significant elevation of NSD2 expression in the senescent BMSCs from aged mice compared with adult controls both at mRNA and proteins levels (Figure 4C,D, Figure S3B). Moreover, when the senescent BMSCs were treated with melatonin for RNA sequencing, we identified 1396 down‐regulated genes and 493 up‐regulated genes (Figure 4E, Table S2); the KEGG pathway annotation showed the DEGs were involved in development, regeneration and aging (Figure 4F), and the GO enrichment analysis for the up‐regulated genes indicated the DEGs were closely related to development and cell differentiation (Figure 4G). Since NSD2 catalyses H3K36me2 modification and activates gene expression, we performed ChIP‐seq assay to determine whether melatonin‐induced genes are associated with H3K36me2 in the senescent BMSCs treated with melatonin. Gene set enrichment analysis identified that up‐regulated genes induced by melatonin were associated with enrichment of H3K36me2 (Figure 4H), and 82 genes were overlapped between melatonin‐induced 1820 differently expressed genes and the H3K36me2 ChIP‐seq peaked genes (Figure 4I, Table S3). KEGG analysis showed that overlapped genes were significantly enriched for the regulation of development, aging and regeneration (Figure 4J), consistent with the role of melatonin in enhancing differentiation of BMSCs.

### 3.5 NSD2 is a direct target of melatonin‐MT1/2 signalling pathway

Given that melatonin stimulates NSD2 expression, we next clarify whether NSD2 is a direct target of melatonin. Studies have revealed that functions of melatonin on BMSCs dependent on its MT1 and MT2 receptors at membrane,^28^ and melatonin activates MAPK, PI3K/AKT, Wnt and NF‐κB signalling pathways in BMSCs.^29^ We confirmed that melatonin activated these signalling pathways in human BMSCs, as evidenced by a dose‐dependent manner of increasing phosphorylation of Extracellular Signal‐regulated Kinase 1/2 (ERK1/2), AKT Serine/Threonine Kinase 1 (AKT), p65 and accumulation of β‐catenin (Figure S4A). Notably, when these signalling pathways were blocked by specific inhibitors, melatonin treatment failed to up‐regulate the NSD2 expression in human BMSCs (Figure S4B). We constructed a 2Kb *NSD2* promoter and transfected into human BMSCs and then treated with increasing dosage of melatonin and found the transcriptional activity of *NSD2* promoter was increased gradually (Figure S4C). After checked putative transcription factor binding sites on *NSD2* promoter by an online library, the PROMO, multiple cis‐elements for transcriptional factors, such as cAMP‐response Element Binding Protein (CREB), Transcription Factor 4/Lymphoid Enhancer Binding Factor 1 (TCF4/LEF1), Nuclear Factor Kappa B (NF‐κB) were identified (Figure S4D). Using ChIP‐PCR, representative TFs of these signalling pathways, such as CREB, RelA and TCF4/LEF1 were all successfully immunoprecipitated on the *NSD2* promoter in human BMSCs (Figure S4E), suggesting the *NSD2* was regulated by multiple transcriptional factors of these known signalling pathways. On the contrary, when the binding sites of these TFs were mutated, luciferase activity of *NSD2*‐promoter induced by melatonin was partially attenuated in human BMSCs (Figure S4F). Thus, these results suggest that *NSD2* is a direct target of melatonin‐MT1/2 signalling pathway.

### 3.6 Melatonin stimulates osteogenesis of senescent BMSCs through NSD2‐mediated chromatin remodelling

**FIGURE 5:**
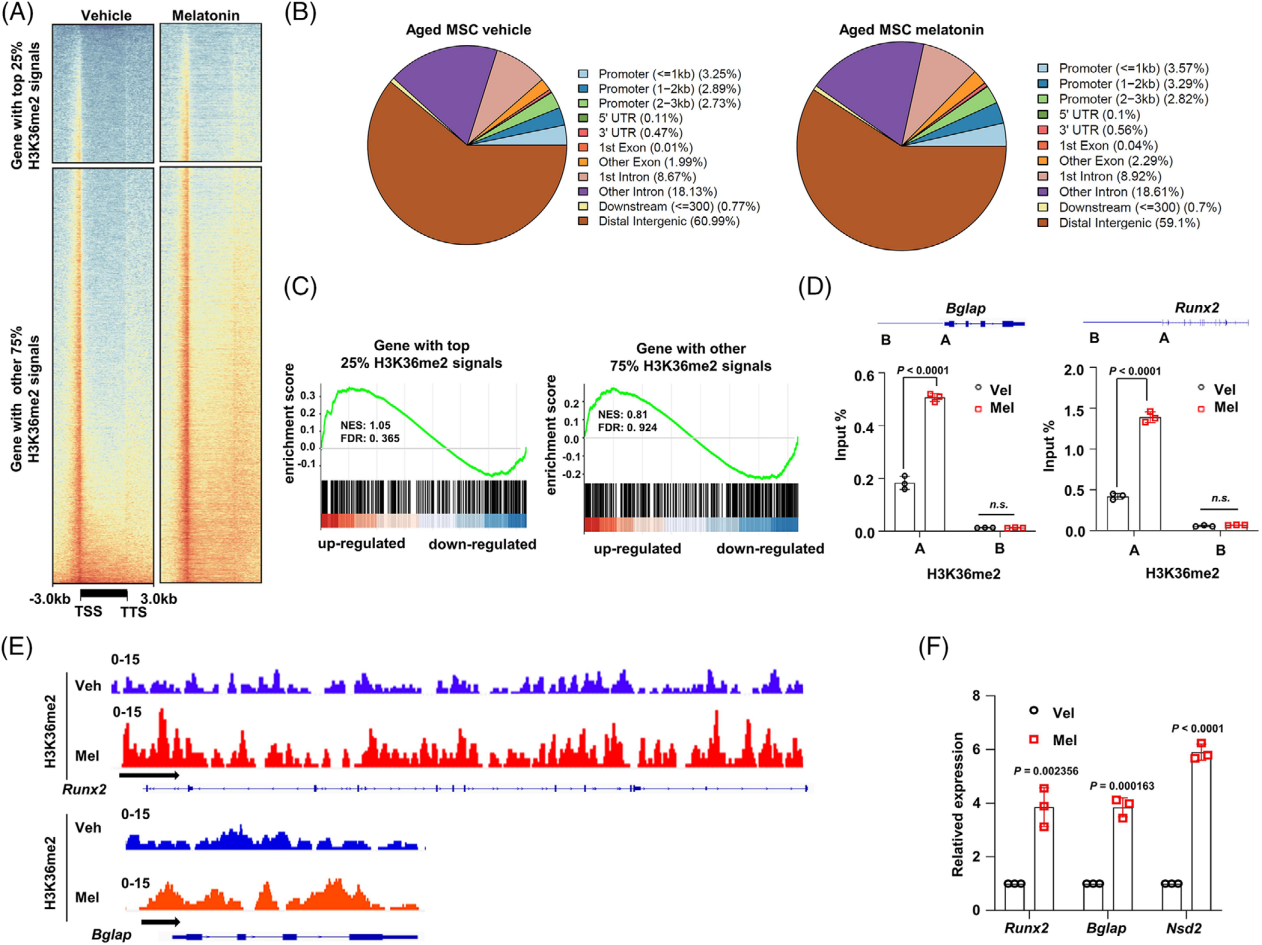
Melatonin promotes H3K36me2 enrichment on osteogenic gene promoters. (A) Genome‐wide distribution of H3K36me2 binding regions in the senescent mouse bone marrow stromal cells (BMSCs) treated with vehicle or melatonin for 24 h (*n* = 3 biological experiments). (B) Pie charts of the distribution of H3K36me2 peaks relative to gene features in the senescent BMSCs treated with vehicle control or melatonin for 24 h. (C) The GSEA enrichment of genes with top 25% H3K36me2 signals and other 75% signals. (D) Chromatin‐immunoprecipitation (ChIP)‐qPCR of H3K36me2 at *Runx2* and *Bglap* gene loci in the senescent mouse BMSCs treated with vehicle or melatonin (*n* = 3 biological experiments). Schematic representation of PCR primer design is provided. (E) Gene tracks of representative ChIP‐seq profiles for the H3K36m2 mark at the *Runx2* and *Bglap* gene loci. (F) qPCR expression analyses of *Runx2, Bglap* and *Nsd2* mRNA expression levels in mouse senescent BMSCs in osteogenic induction medium for 7 days treated with vehicle or melatonin (*n* = 3 biological experiments). Data are mean ± s.e.m. *p* values are determined by paired two‐sided *t*‐tests with Welch's correction

To further interpret the role of melatonin‐induced NSD2 in regulating osteogenesis of BMSCs, we mapped genome‐wide H3K36me2 distribution in melatonin‐treated mouse BMSCs and found a large proportion of H3K36me2 distributed on the gene promoter and transcription start site (TSS) regions, neither gene body nor transcription terminate site regions (Figure 5A). Actually, when analysing the proportion of H3K36me2 distributions, we observed considerable changes occurred in all promoter regions, including ≤1 kb, 1∼2 kb and 2∼3 kb promoters (Figure 5B). The enrichment scores were positively correlated with H3K36me2 occupancies on its target genes, especially when comparing the genes with top 25% H3K36me2 signals to other 75% signals (Figure 5C). Among all the enriched genes by H3K36me2, we verified two key regulators of osteogenesis, *Runx2* and *Bglap*, whose TSS sites of promoter, but not distal intergenic regions, could be enriched by H3K36me2 with melatonin simulation (Figure 5D). Representative tracks profiles of the *Runx2* and *Bglap* genes indicated that changes of H3K36me2 enrichment were occurred mainly in promoter regions (Figure 5E). Importantly, when the BMSCs from aged mice were treated with melatonin, transcriptional activities of *Runx2, Nsd2* and *Bglap* were substantially enhanced, as evidenced by significantly up‐regulated mRNAs (Figure 5F). These data suggest that stimulation on osteogenesis of senescent BMSCs by melatonin is correlated with NSD2‐mediated chromatin modifications.

### 3.7 ATAC‐seq assay identifies chromatin accessibility alteration in melatonin‐treated senescent BMSCs

**FIGURE 6:**
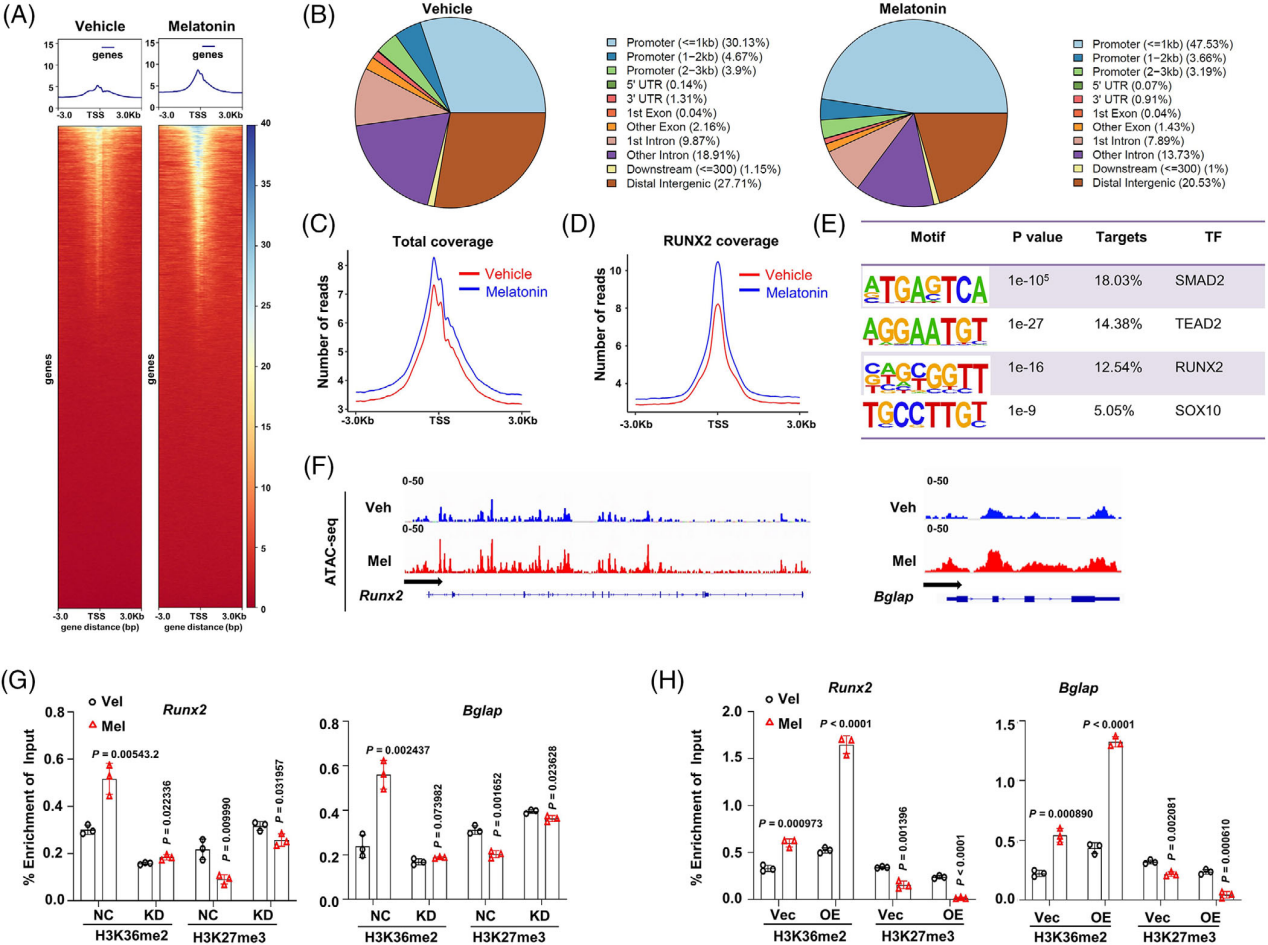
Melatonin favours chromatin accessibility of osteogenic genes. (A) Heat maps of signal distribution around the transcription start site (TSS) ± 3Kb of genes in mouse senescent bone marrow stromal cells (BMSCs) treated with vehicle control or melatonin for 24 h (*n* = 3 biological experiments). (B) Pie charts of the distribution of assay for transposase‐accessible chromatin with high‐throughput sequencing (ATAC‐seq) consensus peaks relative to gene features in mouse senescent BMSCs treated with vehicle control or melatonin for 24 h. (C) Total average coverage of enrichment around the TSS regions in the vehicle control (red line)‐ and melatonin (blue line)‐treated mBMSCs. (D) Average coverage of RUNX2 enrichment around the TSS regions in the veihicle control (red line)‐ and melatonin (blue line)‐treated mBMSCs. (E) Sequences of the most significantly enriched motifs detected in the differentially accessible regions. (F) ATAC‐seq tracks for the identification of accessible chromatin regions of *Runx2* and *Bglap* genes in mouse senescent BMSCs treated with vehicle control or melatonin. Chromatin‐immunoprecipitation (ChIP)‐qPCR assay for changes of H3K36me2 and H3K27me3 enrichment on the *Runx2* and *Bglap* gene promoters in mouse senscent BMSCs, (G) with (nontarget control, NC) or NSD2 kowckdown (KD), (H) with (vector control, Vec) or NSD2 overexpression (OE) (*n* = 3 indepednent experiments). Data are mean ± s.e.m. *p* values are determined by paired two‐sided *t*‐tests with Welch's correction

Given that H3K36me2 modification remodels chromatin to facilitate gene transcription,^30^ we employed ATAC‐seq to investigate the impact of melatonin on chromatin accessibility of BMSCs. Senescent BMSCs from aged mice treated with melatonin or vehicle for 24 h were collected for ATAC‐seq. Global augmentation of chromatin accessibility in the melatonin‐treated BMSCs was observed when compared to the vehicle control (Figure 6A), and the chromatin remodelling occurred mainly at the key regions of promoters (≤1 Kb), but not significance in the distal promoters, gene body, nor untranslated regions (Figure 6B). As expected, genome wide distribution and intensity of ATAC‐seq signal at the TSS regions were elevated after melatonin treatment (Figure 6C), including the osteogenesis transcriptional factor RUNX2 (Figure 6D). Additionally, HOMER de novo motif analysis showed that the most highly ranked transcriptional factors were SMAD2, TEAD2, RUNX2, and SOX10, which are all involved in stem cell maintenance or osteoblast differentiation^31^, ^32^, ^33^ (Figure 6E). We found that ATAC‐seq signal in the promoter regions of *Runx2* and *Bglap* in the melatonin‐treated senescent BMSCs displayed an increase compared with that in the vehicle control (Figure 6F), which is also consistent with the expression pattern of *Runx2* and *Bglap* in these BMSCs as shown in the above Figure 5F. These data clearly indicated that melatonin facilitates the chromatin accessibility of key genes governing osteogenesis in senescent BMSCs.

Previous study has revealed that rebalance of H3K36me2 and H3K27me3 modifications regulates the chromatin accessibility in gene transcription^34^; therefore we evaluated the effect of melatonin on rebalancing these two histone modifications on osteogenic gene promoters. In normal human BMSCs from different ages, we observed that BMSCs with higher level of NSD2 confer more global H3K36m2 and lower H3K27me3 modifications (Figure S5A), which is consistent with the previous report. When NSD2 expression was abolished using lentivirus carrying shRNA (Figure S5B), osteogenesis capacity was significantly suppressed, evidence by remarkably down‐regulated ALP activity and Alizarin red intensity (Figure S5C,D), and vice versa when NSD2 was overexpressed (Figure S5E–G). Notably, when NSD2 was depleted in the senescent mouse BMSCs, and these cells were treated with melatonin, the augmentation of H3K36me2 and suppression of H3K27me3 became indistinctive (Figure 6G); on the contrary when NSD2 was overexpressed, this alteration of these modifications induced by melatonin turn to more remarkable (Figure 6H), accompanied with remarkable *RUNX2* and *BGLAP* expressions (Figure S5H). Moreover, with NSD2 knockdown, osteogenic genes expressions response to melatonin became indolent (Figure S5I), but NSD2 overexpression sensitized the senescent BMSCs to melatonin largely (Figure S5J), both at a dose dependent manner. Collectively, these data suggest melatonin promotes osteogenic gene expression through NSD2‐mediated chromatin remodelling and accessibility.

### 3.8 Melatonin ameliorates osteoporosis in aged mice via up‐regulation of NSD2

**FIGURE 7:**
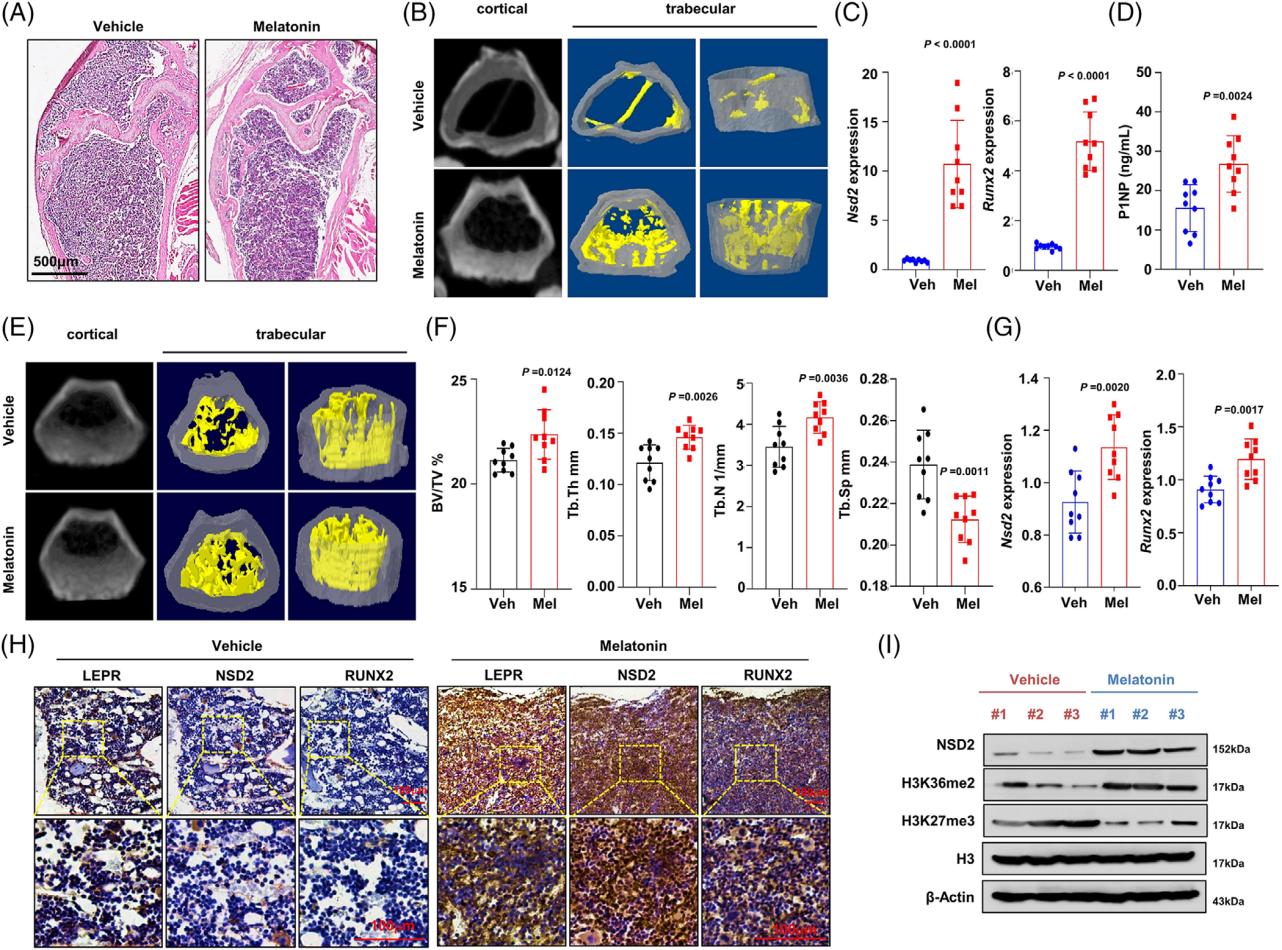
Melatonin facilitates osteogenesis of bone marrow stromal cells (BMSCs) from aging mice via NSD2. (A) Representative hematoxylin and eosin staining of trabecular bone structures of the femur metaphysis from the aged mice treated with vehicle control or melatonin. (B) MicroCT analysis of the femur metaphysis from the aged mice treated with vehicle control or melatonin. Region of interest (ROI), 1 mm length starting from .5 mm proximal to the growth plate consisting of 140 slices. (C) qPCR assay of *Nsd2* and *Runx2* expression in BMSCs and (D) enzyme immunoassay (EIA) assay of P1NP level in serum from the aged mice treated with vehicle control or melatonin. (E) MicroCT analysis of the femur metaphysis from the adult mice treated with vehicle control or melatonin (ROI: a 1 mm length starting from .5 mm proximal to the growth plate). (F) Quantification of microCT analysis of bone volume ratio to tissue volume (BV/TV), trabecular thickness (Tb.Th), and trabecular number (Tb.N) and trabecular separation (Tb.Sp) in adult mice treated with vehicle control or melatonin (*n* = 9). (G) qPCR assay of *Nsd2* and *Runx2* expression in BMSCs from the adult mice treated with vehicle control or melatonin. Data are mean ± s.e.m. *p* values are determined by unpaired two‐sided *t*‐tests with Welch's correction. (H) Immunohistochemistry assay for the expression of leptin receptor (LPER) as the marker of BMSCs, NSD2 and RUNX2 in the sequential slide of femur tissue from the aged mice treated with vehicle control or melatonin. Scale bar, 100 μm. (I) Western blotting assay of the NSD2, H3K36me2 and H3K27me3 level in BMSCs from the aged mice treated with vehicle control or melatonin. Data are mean ± s.e.m. *p* values are determined by paired two‐sided *t*‐tests with Welch's correction

We evaluated the effects of melatonin on ameliorating osteoporosis in an aging mouse model. Aged mice (18 months old) and young adult mice (2 months old) were treated with vehicle or melatonin by subcutaneous injection for 10 weeks. H&E staining for histological structure of the metaphysis showed that melatonin treatment, but not vehicle control, noticeably increased the numbers of bone trabecula in the aged mice femur (Figure 7A). The micro‐CT assay showed visible improvement in the femoral trabecular microstructure (Figure 7B), evidenced by a remarkable recuperation of trabecula disruption in the melatonin treatment group, with higher percentages of bone volume density (BV/TV), Tb.N, cortical thickness (Tb.Th), and lower size of Tb.Sp (Figure S6A). Meanwhile, *Nsd2* and *Runx2* expression in BMSCs from melatonin‐treated aged mice was significantly elevated (Figure 7C). Next, we measured the level of serum procollagen type 1 N‐terminal propeptide (P1NP), a marker for new bone formation, and found that serum P1NP was remarkably elevated in the aged mice treated with melatonin (Figure 7D). However, melioration of femoral trabecular microstructure and up‐regulation of *Nsd2* and *Runx2* (Figure 7E–G) were not so exceptional in the young mice compared with that in the aged ones. Co‐localized by the expression of leptin receptor, a marker of BMSCs, we also validated the NSD2 and RUNX2 protein levels were both remarkably up‐regulated compared with the vehicle control (Figure 7H). As expected, BMSCs from aged mouse treated with melatonin, but not vehicle control, exhibited much extensive elevation in osteogenesis capacity compared with the adult control (Figure S6B–D). Meanwhile, the suppressed NSD2 and global H3K36me2 levels were significantly rescued, but H3K27me3 level was inhibited by melatonin treatment in the senescent BMSCs (Figure 7I, Figure S6E). Overall, these data from aged mice support the hypothesis that melatonin ameliorates osteogenesis and bone mass of aged mice in an NSD2‐dependent way.

### 3.9 Melatonin treatment recovers the osteogenic potential of BMSCs derived from patients with SOP

**FIGURE 8:**
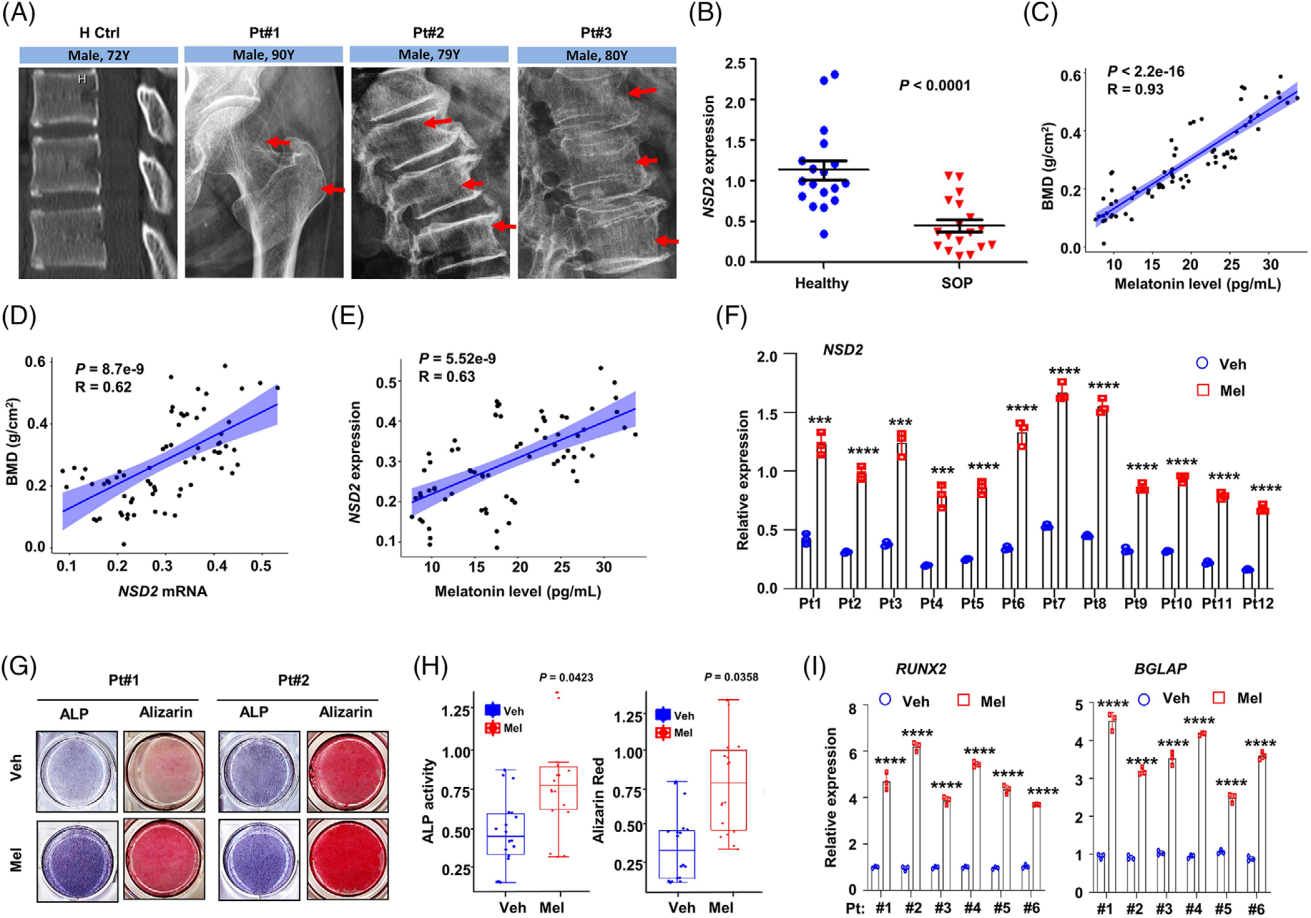
Melatonin recovers osteogenic potential of bone marrow stromal cells (BMSCs) from patients with senile osteoporosis. (A) Radiographic images of healthy control (H Ctrl) and patients with senile osteoporosis (Pt). Red arrows indicate the typical area with osteoporosis. (B) *NSD2* expression in BMSCs isolated from healthy control and patients with senile osteoporosis (*n* = 18 with each detection triplicated). (C) Correlation of bone mass density (BMD) with melatonin level in bone marrow plasma. (D) *NSD2* expression in BMSCs from senile osteoporosis patients (three independent reads for *n* = 12 patients). (E) Correlation of *NSD2* expression in BMSCs and melatonin level in bone marrow plasma of patients with senile osteoporosis (three independent reads for *n* = 12 patients). (F) *NSD2* expression in 12 BMSCs from patients with senile osteoporosis treated with 1 μM melatonin for 24 h (*n* = 3 with each detection triplicated). (G) Representative images of alkaline phosphatase (ALP) and Alizarin Red S staining in BMSCs from donors with senile osteoporosis treated with osteogenic media for 14 days in the presence of vehicle or melatonin. (H) Quantification of ALP and Alizarin Red S staining for BMSCs under osteogenesis induction and in the presence of Dimethyl Sulfoxide (DMSO) vehicle or melatonin (three independent reads for *n* = 6 BMSCs). (I) mRNA levels of *RUNX2* and *BGLAP* in BMSCs under osteogenesis induction and in the presence of vehicle or melatonin (*n* = 3 with each detection triplicated). Data are mean ± s.e.m. *p* values are determined by two‐sided *t*‐tests with Welch's correction. ***p < .001; ****p < .0001

Finally, we investigated the association between NSD2 and SOP in the clinical patients. BMSCs were collected from bone biopsy samples from healthy elderly individuals and patients with SOP (Figure 8A), and bone mineral density of SOP patients was examined by dual energy X‐ray absorptiometry. Similar to what we have found in the aging mouse model, *NSD2* expression was remarkably suppressed in BMSCs from donors with SOP than in those from age‐matched control donors (Figure 8B). There was a significant positive correlation between bone mineral density and melatonin levels in bone marrow in patients with SOP (Figure 8C), and between bone mineral density and *NSD2* expression (Figure 8D). Moreover, *NSD2* expression showed a very strong correlation with melatonin levels in bone marrow (Figure 8E). Accordingly, melatonin (1 μM) treatment enhanced *NSD2* expression in MSCs derived from patients with SOP (Figure 8F) and improved their differentiation efficiency after osteogenic induction, as evidenced by higher levels of ALP activity, increased numbers of mineralized nodules (Figure 8G,H) and up‐regulated *RUNX2* and *BGLAP* expression (Figure 8I). These data strongly suggest that NSD2 down‐regulation is a characteristic of aging‐associated osteoporosis and imply that melatonin is efficient to restore osteogenesis capacity of BMSCs in patients with SOP.

## 4 DISCUSSION

In the current study, we report that melatonin in bone marrow decreases in an aging‐related manner, and treatment with melatonin can reverse the impaired osteogenic potential of senescent BMSCs through an epigenetic regulatory mechanism mediated by the histone methyltransferase NSD2. Our study therefore supports the application of melatonin as a potent therapeutic agent for the prevention of aging‐associated osteoporosis.

Melatonin is known to modulate bone formation and osteoblast differentiation of BMSCs.^9^ Studies have revealed that melatonin can boost osteogenic differentiation by up‐regulating osterix protein stability and expression,^12^ reduce autophagy activation in high glucose‐cultured osteoblasts and meliorate diabetes‐induced osteoporosis by inhibiting ERK pathway.^35^ However, it is generally unknown how melatonin promotes the osteogenic potential of BMSCs undergoing aging. In this study, we report that melatonin in bone marrow is declined with aging, and NSD2 (also known as MMSET or WHSC1) is an important factor mediating the effects of melatonin. Melatonin treatment effectively improves NSD2 expression and rebalances the distribution of H3K36me2 and H3K27me3 modifications on osteogenic gene promoters in the senescent BMSCs, consequently modulates the chromatin accessibility of osteogenic genes. By contrast, RNA interference‐mediated *NSD2* depletion attenuates melatonin‐reversed osteogenic differentiation. NSD2 is a histone methyltransferase that catalyses lysine methylations of histones H3 and H4.^36^ NSD2 overexpression leads to an augmentation in H3K36me2 and a suppression in H3K27me3 across the genome, causing a looser chromatin structure.^34^ However, the relationship between NSD2 with aging and osteoporosis has not been reported previously, and this is the first description of the importance of this mediator in BMSC senescence. Our results established a link between NSD2 expression and aging. We identified about 40 genes whose expressions were altered during the senescence of human BMSC, most of that are stemness‐related or senescence‐related, such as *CCDC28B, SLC19A1, SOX9, ABCG2, NANOG, RUNX2* and *CDKN2A* as expected. The results also revealed that *NSD2* was remarkably decreased in the senescent BMSCs derived from elderly donors, which is also seen in the BMSCs from aged mouse. Our study also indicates that NSD2 down‐regulation in senescent BMSCs is accompanied by decreased osteogenic potential, and NSD2 ablation effectively abolished the melatonin‐enhanced osteogenic differentiation of senescent BMSCs. Thus, NSD2 down‐regulation seems to be an important characteristic of senescent BMSCs, and it weakens the osteogenic differentiation potential of BMSCs. SOP arises from multiple age‐related events associated with the aberrant expression of multiple genes, and accumulating evidence links aging‐associated osteoporosis to epigenetic alterations.^37^ Epigenetic mechanisms are very important in the acquisition of cell fate in stem cells. Given that NSD2 is a histone methyltransferase, we speculate that it affects osteogenic differentiation via epigenetic mechanisms. Ample evidences have indicated that H3K36me2 regulates transcription activity around the TSS region, such as antagonizing Polycomb Repressive Complex 2‐mediated transcription silencing through H3K27me3, and acts as a safeguard to ensure transcription fidelity.^38^ Our ChIP‐seq and ATAC‐seq data reveal that melatonin rebalances the distribution of H3K36me2 and H3K27me3 modifications on the osteogenic gene promoters. Therefore, melatonin meliorates bone loss through enhancing transcription activities of *RUNX2* and *BGLAP* through modifying the chromatin accessibility status around the TSS region of osteogenic gene promoter. However, other cis‐elements or enhancer located far away from the TSS region has no significant changes. These results strongly indicate that NSD2 renders the differentiation propensity of BMSCs towards osteoblasts via an epigenetic mechanism. Although our study suggested NSD2 is a critical responder to melatonin treatment in ameliorating the loss of bone, it is probably only one part of the consequence of senescence and one that could be of general relevance, since the loss of expression results in a general impact on loss of bone. Other genes playing important roles in osteogenesis, or an integrated role under melatonin stimulation, should be considered.

Taken together, our study identified NSD2 as a modulator of melatonin‐mediated osteogenesis. Melatonin modifies the chromatin remodelling of osteogenic gene promoter in BMSCs to promote bone formation. Nevertheless, the current study focuses on restoring and accentuating bone formation using melatonin, but the in vivo bone dynamics comprise osteoblast‐derived bone formation and osteoclast‐driven resorption.^39^ Thus, apart from the assessment of bone formation in response to melatonin, the residual components of bone have not been investigated in this study, although recent reports have revealed inhibitory effects of melatonin on osteoclastogenesis.^27^, ^40^ Despite the benefit of melatonin on aging‐associated osteoporosis, the effect of melatonin on estrogen deficiency‐induced osteoporosis has not been investigated in the present study, which deserves further investigations.

## CONFLICT OF INTEREST

The authors declare no competing financial interests.

## Supporting information

Table S1

Table S2

Table S3

Supporting Information

Figure S1‐S6
