# Supplementary material for: Melatonin enhances osteoblastogenesis of senescent bone marrow stromal cells through NSD2‐mediated chromatin remodelling
Source: Clin Transl Med. 2022 Feb 27;12(2):e746. doi: 10.1002/ctm2.746 (PMC8882236; doi:10.1002/ctm2.746)
Supplement: Supplementary file 1 — Table S1 [file CTM2-12-e746-s004.docx]

**Table S 1 DEGs of mouse BMSCs aged versus adult**

| **Down genes** | **Up genes** |
| --- | --- |
| Zfpm2 | Mmp13 |
| Zfp967 | Cfh |
| Zfp9 | Crispld2 |
| Zfp882 | Slc25a45 |
| Zfp827 | Myom1 |
| Zfp57 | Sp7 |
| Zfp521 | Synpo2 |
| Zfp469 | Glipr1 |
| Zfp395 | Ackr4 |
| Zfp184 | Efnb1 |
| Zfhx4 | Loxl2 |
| Zeb1 | Ibsp |
| Zcchc3 | S100b |
| Zc3hav1l | Vsig4 |
| Xkr5 | Ampd3 |
| Wscd2 | Trf |
| Wasf1 | A530064D06Rik |
| Vat1l | Lrmda |
| Uty | Clec5a |
| Utp14b | Emp3 |
| Usp11 | Prdx6 |
| Unc5c | Mmp3 |
| Ugt1a5 | Nlrp3 |
| Uck2 | Abcc3 |
| Twist1 | Ccr5 |
| Ttll5 | Galnt6 |
| Ttc3 | Slc7a7 |
| Ttbk1 | Folr2 |
| Tspyl5 | Ccl12 |
| Tspan15 | Dpep2 |
| Trpv1 | Slc16a7 |
| Trps1 | Ica1l |
| Trim66 | Tgtp2 |
| Trib3 | H2-Q9 |
| Traip | Mylk |
| Tnmd | Nxpe5 |
| Tnfaip8l3 | Prxl2b |
| Tnfaip6 | Slco2b1 |
| Tmtc2 | Fcna |
| Tmod2 | Cd300ld |
| Tmem74 | Myo1f |
| Tmem254c | Bank1 |
| Tmem169 | Abcg1 |
| Tmem158 | Otulinl |
| Tlcd4 | Hck |
| Thbs3 | Msr1 |
| Tgif2 | B430306N03Rik |
| Tgfbr3l | Shtn1 |
| Tfap4 | Ugt1a7c |
| Tex26 | Ccl9 |
| Tet1 | Trpv4 |
| Tenm3 | Hhex |
| Tcim | Tmem273 |
| Tcf4 | Pira2 |
| Tbx4 | Mmp12 |
| Tbx3 | Wdfy4 |
| Tbx20 | Cfp |
| Tbx18 | Alox5ap |
| Taf7l | Bcam |
| Syde2 | Ifi204 |
| Sybu | Selplg |
| Sulf1 | Gpsm3 |
| Steap4 | Itgb2 |
| Stc2 | P2ry12 |
| Sspo | Cd180 |
| Ssc5d | Tal1 |
| Ssbp2 | Pld4 |
| Srd5a1 | Mmp27 |
| Spx | Ccl6 |
| Sptbn2 | Dock2 |
| Spta1 | Sorbs2 |
| Spock2 | Hpgds |
| Spink2 | Hk3 |
| Spin4 | Ccr1 |
| Spats2l | Susd3 |
| Sox2 | Trpm2 |
| Sox12 | Cd68 |
| Sorcs2 | Xlr |
| Socs1 | Barx1 |
| Smarca1 | Arap3 |
| Slitrk6 | H2-DMb1 |
| Slit2 | Adam8 |
| Slc7a5 | Casp1 |
| Slc7a3 | Ncf2 |
| Slc6a9 | Myo1g |
| Slc5a7 | Tmem51 |
| Slc4a11 | Ptprc |
| Slc38a4 | Cd300lb |
| Slc25a27 | Tlr13 |
| Slc24a3 | Trpv2 |
| Slc1a3 | Themis2 |
| Slc16a1 | Bin2 |
| Skida1 | B3gnt5 |
| Six4 | 2200002D01Rik |
| Shroom2 | Ncf1 |
| Sh3rf3 | Tnfaip8l2 |
| Sh2d6 | Cadm1 |
| Sfrp2 | Ctsc |
| Sfmbt2 | Pik3cg |
| Setbp1 | Lrrc25 |
| Sesn3 | Clec4a3 |
| Sertad4 | Pdgfb |
| Serinc4 | Tmem106a |
| Septin6 | Mpeg1 |
| Sema6a | Trem2 |
| Sema3f | Prkch |
| Sema3a | Milr1 |
| Sec16b | Gbp5 |
| Scx | Csf2rb |
| Scn3a | Igf1 |
| Scd1 | Tlr7 |
| Scarf2 | Adap2 |
| Sardh | Fcgr2b |
| Sapcd2 | Bcl2 |
| Runx2 | Cd300lf |
| Rtkn | Csf2rb2 |
| Rpp25 | Il7r |
| Robo2 | Gda |
| Rnf43 | Wfdc17 |
| Rnf165 | Lilra5 |
| Rnf122 | Nckap1l |
| Rhobtb3 | Malrd1 |
| Rgs6 | Fgf13 |
| Rgma | Lair1 |
| Rflna | Cd84 |
| Rcc2 | Gm14548 |
| Rassf8 | Pygl |
| Rasl11b | Tm6sf1 |
| Ramp3 | Lpxn |
| Rab9 | Pf4 |
| Rab39b | Ctss |
| Rab27b | C5ar1 |
| Pycr1 | Lmo2 |
| Pxdn | Csf1r |
| Ptn | Lilr4b |
| Ptgis | Mrc1 |
| Ptchd1 | Igsf6 |
| Psip1 | Lilrb4a |
| Psat1 | Fap |
| Prx | Plek |
| Prtg | Atp8b4 |
| Prrt2 | Akr1c12 |
| Prkaa1 | Trem1 |
| Prickle2 | Lcp1 |
| Prickle1 | Atp1a3 |
| Prdm8 | Nfam1 |
| Ppp4r4 | Coro2a |
| Ppm1l | Lyz2 |
| Ppargc1a | Cd163 |
| Postn | Irf5 |
| Podn | Cd200r1 |
| Pmch | Abhd15 |
| Plxna3 | Arhgap25 |
| Plk2 | Nr1h3 |
| Plekhg5 | Ret |
| Plekha5 | Adcyap1r1 |
| Plekha4 | Ms4a6d |
| Plat | Il2rg |
| Plagl1 | Pkib |
| Pknox2 | Cd37 |
| Pkhd1l1 | Rassf3 |
| Pitx1 | Fyb |
| Pik3ip1 | Fcer1g |
| Pi15 | Cd300a |
| Phgdh | Cd36 |
| Phex | Ptpn6 |
| Pgm5 | Arhgap45 |
| Penk | Cd72 |
| Peg10 | Epsti1 |
| Pear1 | Gsta3 |
| Pdzrn3 | Tlr8 |
| Pdzd7 | Tbxas1 |
| Pdgfrl | Cd33 |
| Pde9a | Zc3h12d |
| Pcsk4 | Lgals3 |
| Pck2 | Cacna1d |
| Pcdhga3 | Fmnl1 |
| Pcdhb20 | Cyth4 |
| Pcdh7 | Inpp5d |
| Pcdh10 | Itgam |
| Pbx1 | Tfec |
| Patj | Rnase6 |
| Pard6b | Gm21451 |
| Pard3b | Adap1 |
| Pabpc4l | Btk |
| P2rx5 | Clec4n |
| Osbpl6 | Adrb1 |
| Oprk1 | Blnk |
| Olfml1 | AB124611 |
| Obsl1 | Crybg1 |
| Nsd2 | Bcl2a1b |
| Nrip2 | Fat3 |
| Nrg1 | Lcp2 |
| Nr4a2 | Mtarc1 |
| Npy1r | Vav1 |
| Nptx1 | Frmd4b |
| Npas2 | Fcgr3 |
| Nova1 | Ptpro |
| Nhsl1 | Neurl3 |
| Nfkb1 | C130026I21Rik |
| Nfix | Tnfrsf11a |
| Nfil3 | Icam1 |
| Nfia | Clec4d |
| Nfatc4 | Was |
| Neto2 | Itgax |
| Nedd9 | Ly9 |
| Ndst3 | Cd52 |
| Ncald | Laptm5 |
| Nanog | Ms4a6c |
| N4bp2 | Stap1 |
| Myzap | Rab32 |
| Myh10 | Stard8 |
| Muc1 | Cd300c2 |
| Mtss2 | Icosl |
| Mtcl1 | Bmp2 |
| Msln | P2ry6 |
| Mrgprf | Hcls1 |
| Mmp14 | Cd22 |
| Mki67 | Lpar5 |
| Mgp | Cd28 |
| Mfap4 | LOC102638047 |
| Mfap2 | Oas3 |
| Mex3a | AI467606 |
| Met | Prex1 |
| Meox2 | Lyz1 |
| Meox1 | Cybb |
| Megf10 | Stxbp2 |
| Mecom | Marchf1 |
| Mcc | Lyn |
| Mboat1 | Nectin4 |
| Mapk8 | Ehd3 |
| Mapk1 | Slamf9 |
| Map2 | Bst1 |
| Macrod2 | Adgb |
| Lypd6 | Snx20 |
| Lvrn | Cd40 |
| Lurap1 | Entpd1 |
| Ltbp4 | Cd53 |
| Lrrtm3 | Apobec1 |
| Lrrc71 | Pde1b |
| Lrig3 | Zfp979 |
| Lrig1 | Rgs14 |
| Lrfn4 | Slc15a3 |
| Lpin3 | Smagp |
| Lpar2 | Siglec1 |
| Loxl1 | Fcgr1 |
| LOC115489542 | Ch25h |
| LOC115488023 | Gm21188 |
| Lmnb1 | Grk3 |
| Lhx9 | Nlrp1b |
| Lgi2 | Nfatc2 |
| Ldb2 | Samsn1 |
| Lck | Pgap6 |
| Lcat | C1qa |
| Lamc1 | Cd93 |
| Klhl29 | Lat2 |
| Klf5 | C5ar2 |
| Klf12 | Nlrp1a |
| Kif21a | Csf3r |
| Kdm5d | Apoe |
| Kcnma1 | Dok2 |
| Kcnip1 | Cxcr3 |
| Kcnc2 | Clec12a |
| Kcna4 | Mmp9 |
| Itih2 | Ms4a14 |
| Itgb3 | Slc43a2 |
| Itga2b | Vsir |
| Isoc1 | Adgre1 |
| Inava | Fgd2 |
| Il33 | Iqgap2 |
| Il1rapl1 | S100a1 |
| Il17rd | Kng1 |
| Igsf10 | Sirpa |
| Igf2bp2 | Slfn5 |
| Ift81 | Cysltr1 |
| Htr7 | Cd300ld3 |
| Hspa12a | C1qb |
| Hpdl | Fermt3 |
| Hoxd9 | Arhgap30 |
| Hoxd8 | Arhgap9 |
| Hoxc12 | P2ry14 |
| Hopx | Arhgap4 |
| Hmgb2 | Slc11a1 |
| Hmgb1 | Hnmt |
| Hmcn1 | Cyfip2 |
| Hlf | Gvin1 |
| Hivep2 | Tnfaip3 |
| Hid1 | Lyl1 |
| Hic1 | Irf8 |
| Hhip | Pik3r5 |
| H4c11 | Angptl7 |
| H1f10 | Abcb4 |
| H1f0 | Rapgef5 |
| Gxylt2 | Sncaip |
| Grik2 | Pla2r1 |
| Gpt2 | Pilrb1 |
| Gprc5a | Arl11 |
| Gprasp2 | Ucp2 |
| Gpr88 | Sema4d |
| Gpr62 | Tmem221 |
| Gpr153 | Cxcl16 |
| Gnat2 | Aldh2 |
| Gm9241 | Pik3ap1 |
| Gm52666 | Rasgef1b |
| Gm42346 | Pirb |
| Gm3993 | Arrb2 |
| Gm38699 | Rac2 |
| Gli1 | Ccdc88b |
| Glce | S100a16 |
| Glcci1 | Naip5 |
| Gfra2 | Cd244a |
| Gfod1 | Sh3bp2 |
| Gemin8 | Ssh3 |
| Gem | Tmem141 |
| Gdf7 | Sla |
| Gdf1 | C1qc |
| Gcnt4 | Cd200r4 |
| Gcat | Aatk |
| Gata3 | Dock10 |
| Gask1a | Ccl4 |
| Gas1 | Pycard |
| Garem1 | Smpdl3a |
| Galnt17 | Sash3 |
| Gal3st3 | Slc7a8 |
| Gabbr1 | Slc46a3 |
| Fzd6 | Ptafr |
| Fzd2 | C3ar1 |
| Fsbp | Map3k9 |
| Frmpd4 | Creg1 |
| Fras1 | 5430427O19Rik |
| Foxo6 | Napsa |
| Foxo4 | St8sia4 |
| Foxo3 | Hcar2 |
| Foxg1 | Ncf4 |
| Fndc4 | Spi1 |
| Fndc1 | Ankrd33b |
| Flvcr2 | Fabp7 |
| Flt1 | Tlr1 |
| Flrt3 | Atp6v0d2 |
| Flnc | Dock8 |
| Fjx1 | LOC101055663 |
| Fgf5 | Sirpb1b |
| Fgf21 | Asb2 |
| Fgd1 | Lrmp |
| Fbn2 | Kcnk13 |
| Fbln5 | Oas1g |
| Fbln1 | Bco2 |
| Fat4 | Npl |
| Farp1 | Lyzl4 |
| Fancf | Ms4a4a |
| Fam228b | Tmem156 |
| Fam180a | Cndp2 |
| Fam131b | Abcg3 |
| F3 | Gm1966 |
| F2rl1 | Gpr137b |
| F11r | Ikzf1 |
| Erg | Grap |
| Ephb6 | Stab1 |
| Ephb3 | Cd48 |
| Epha7 | Prkcb |
| Epha4 | Sh3tc1 |
| Epha3 | Gm7609 |
| En1 | Lpcat2 |
| Elovl6 | Dnmt3l |
| Eln | Lilra6 |
| Elavl2 | Il18bp |
| Egr3 | Cxcr4 |
| Egfl7 | Gpr84 |
| Egfl6 | Trim29 |
| Efna1 | Pparg |
| Edil3 | Rinl |
| Dpyd | Mbp |
| Dock9 | Chst8 |
| Dnmt3 | Gm38510 |
| Dll1 | Ms4a6b |
| Dennd3 | Alpl |
| Ddit3 | Isyna1 |
| Ddah2 | Rasal3 |
| Dchs2 | Unc93b1 |
| Dchs1 | Ccl7 |
| Daam2 | Gmfg |
| Cyp7b1 | Cebpa |
| Cyp2c23 | F13a1 |
| Cx3cl1 | Avpr1a |
| Cttnbp2 | Rtn4rl1 |
| Cthrc1 | Clec4e |
| Cth | Lst1 |
| Ctdsp2 | Cotl1 |
| Cspg4 | Cnr2 |
| Csdc2 | Mcfd2 |
| Cryz | Renbp |
| Crabp2 | C130050O18Rik |
| Crabp1 | Gpx3 |
| Cplx2 | Abr |
| Coro2b | Efhd2 |
| Col3a1 | Psmb8 |
| Col27a1 | Rab19 |
| Col1a2 | Slc39a12 |
| Col1a1 | Dok3 |
| Col16a1 | I830077J02Rik |
| Col12a1 | Gatm |
| Col11a1 | Adgrf5 |
| Cnksr3 | Gm5431 |
| Cmya5 | Oas1a |
| Clip4 | Gm7592 |
| Clec1a | Card9 |
| Cldn34c2 | Gdf10 |
| Cldn19 | Lrp4 |
| Chst10 | Prss46 |
| Chek1 | Aim2 |
| Chac1 | Tnfsf13b |
| Cftr | Parvg |
| Cfap20dc | Cd5l |
| Ces1a | Apol6 |
| Cdrt4 | Spon1 |
| Cdon | Plxnb3 |
| Cdkn1b | Cd38 |
| Cdkl5 | Crip1 |
| Cdh3 | Tnfsf13 |
| Cdh24 | Psd2 |
| Cd34 | Cd83 |
| Cd276 | Ms4a7 |
| Cd109 | Klhl6 |
| Ccn3 | Cdkn1a |
| Ccdc8 | Cln3 |
| Ccdc3 | Mcoln2 |
| Ccdc18 | Aif1 |
| Ccdc141 | Acvrl1 |
| Ccdc136 | Il21r |
| Cbx5 | Plcb2 |
| Cbfb | Gm4070 |
| Cavin4 | Coro1a |
| Card10 | Nlrc4 |
| Car10 | Cxcl14 |
| Capn6 | Srgn |
| Cand2 | Tmem104 |
| Cacna1c | Rps6ka1 |
| Cachd1 | Cmklr1 |
| C1qtnf6 | Syngr1 |
| C1qtnf1 | Bdkrb1 |
| Bnc2 | Fcrl1 |
| Bmpr1b | Eif4e3 |
| Bmf | Alox5 |
| Bicd1 | Tmem71 |
| Bend5 | H2-D1 |
| Bdh2 | Pla2g7 |
| Bcat1 | Vav3 |
| BC030500 | Il10rb |
| B3gnt9 | Dmpk |
| AW551984 | Sgk3 |
| Avil | Ptprj |
| Auts2 | Cyp4f18 |
| Atrnl1 | Lpl |
| Atp8b1 | Clec4a2 |
| Atp1b1 | Plcg2 |
| Atf4 | Mag |
| Asxl3 | Fpr1 |
| Aspn | Vnn3 |
| Armcx6 | Abi3 |
| Arl5b | Gdf15 |
| Arhgap33 | Dhrs3 |
| Arhgap32 | Cd59a |
| Arhgap20 | Ctsb |
| Apod | Fmo3 |
| Apcdd1 | Zmynd15 |
| Ano5 | Casz1 |
| Ankrd34a | Mgat4a |
| Ank2 | Gm12250 |
| Angptl6 | H2-Q6 |
| Aldh1l2 | S1pr1 |
| Aldh18a1 | Creg2 |
| Akt1 | Il10ra |
| Akr1c14 | Tmem171 |
| Adm2 | Unc13d |
| Adh7 | Tmem243 |
| Adh1 | Ccl2 |
| Adgrl3 | Cidec |
| Adgra2 | Blvrb |
| Adamts6 | LOC115485726 |
| Adamts4 | Rab20 |
| Adamts20 | Fam78a |
| Acvr2a | Akr1c18 |
| Acsl3 | Cd207 |
| Acot2 | Slfn10-ps |
| Acot1 | Rnf149 |
| Abi3bp | Rcsd1 |
| Aadat | Prss16 |
|  | Atp8a1 |
|  | Susd5 |
|  | Ms4a4c |
|  | Adcy5 |
|  | Spic |
|  | Thsd4 |
|  | Gngt2 |
|  | Orai2 |
|  | Angpt2 |
|  | Dennd1c |
|  | Sh3bgr |
|  | Cd14 |
|  | Tmem140 |
|  | Itga4 |
|  | Dhdh |
|  | Mrap |
|  | Inpp4b |
|  | Pou2f2 |
|  | Ctsd |
|  | Ckb |
|  | Kit |
|  | Fes |
|  | Arrdc4 |
|  | Itgal |
|  | Pon3 |
|  | Tifab |
|  | Pik3r6 |
|  | Mfsd13a |
|  | Tnip3 |
|  | Kcnab2 |
|  | Arrb1 |
|  | Tmc8 |
|  | Ggt5 |
|  | Ptpn18 |
|  | B3gnt8 |
|  | Msrb1 |
|  | Il16 |
|  | Pnpo |
|  | Slc37a2 |
|  | Slc13a3 |
|  | Mlph |
|  | H2-K1 |
|  | Dpep3 |
|  | Psd4 |
|  | Ric3 |
|  | Ces1g |
|  | Rbks |
|  | Fut7 |
|  | Htr1b |
|  | Lrrc27 |
|  | Tmem26 |
|  | Ecscr |
|  | Siglece |
|  | Akirin1 |
|  | Sfrp4 |
|  | Tspan32 |
|  | Mfsd6 |
|  | Inpp5j |
|  | Sirpb1a |
|  | Arhgap27 |
|  | Map3k5 |
|  | Scarf1 |
|  | Acp2 |
|  | A630001G21Rik |
|  | Tmem229b |
|  | Gpr141 |
|  | Calcrl |
|  | Fxyd5 |
|  | Gpr65 |
|  | Psma8 |
|  | Ceacam10 |
|  | Kprp |
|  | Cacna1f |
|  | Xlr4b |
|  | Tpbgl |
|  | Sema6b |
|  | Syt3 |
|  | Epas1 |
|  | H2-M3 |
|  | Rab7b |
|  | Sp110 |
|  | Npy |
|  | Pitpna |
|  | Ikbke |
|  | Rgs18 |
|  | Il1a |
|  | Batf2 |
|  | Cd55 |
|  | Arg1 |
|  | Dnase1l1 |
|  | Rhbdf2 |
|  | Gm6377 |
|  | Slc28a2 |
|  | Apoc2 |
|  | Lacc1 |
|  | Fam53b |
|  | Rgs1 |
|  | Nrros |
|  | Nrg4 |
|  | Tlr2 |
|  | Rnf130 |
|  | Mfng |
|  | Id2 |
|  | Pstpip1 |
|  | Stk10 |
|  | Hcst |
|  | Angpt4 |
|  | Ccr3 |
|  | Selenop |
|  | Treml1 |
|  | Slc39a4 |
|  | Lhfpl4 |
|  | Gimap9 |
|  | Exoc3l4 |
|  | Ccrl2 |
|  | Plxdc1 |
|  | Rnpep |
|  | Lgmn |
|  | Unc13a |
|  | Fnd3c2 |
|  | Lgals9 |
|  | Slfn4 |
|  | Flt4 |
|  | Maf |
|  | Ninj1 |
|  | Fam174a |
|  | Sh3bgrl2 |
|  | Ptger2 |
|  | Scly |
|  | Stk17b |
|  | Mgat5 |
|  | Lrg1 |
|  | Cysltr2 |
|  | Fgf11 |
|  | Dnajb13 |
|  | Kcnk6 |
|  | Trarg1 |
|  | Nkx3-2 |
|  | Zeb2 |
|  | Unc5a |
|  | LOC115486430 |
|  | Hgsnat |
|  | Tgm2 |
|  | Gpr183 |
|  | Fmo2 |
|  | Spn |
|  | Akr1b10 |
|  | Klra2 |
|  | Dab2 |
|  | Ebi3 |
|  | Taldo1 |
|  | H2-Ab1 |
|  | Pkd1l2 |
|  | Syp |
|  | G6pdx |
|  | Fads6 |
|  | Man2b1 |
|  | Orai1 |
|  | Gpr34 |
|  | Pcdhga10 |
|  | Slfn2 |
|  | Hmox1 |
|  | Macir |
|  | Acp5 |
|  | Fgr |
|  | Nbeal2 |
|  | Rims3 |
|  | Naip6 |
|  | Rhoh |
|  | Dio3 |
|  | Cat |
|  | Rnaset2a |
|  | Map11 |
|  | Pid1 |
|  | Fbxl2 |
|  | Mtss1 |
|  | Afp |
|  | Cracr2b |
|  | Adipoq |
|  | Efs |
|  | Cracr2a |
|  | Tcn2 |
|  | Mocos |
|  | Stac2 |
|  | Psmb9 |
|  | Clec1b |
|  | Casp4 |
|  | Slc13a2 |
|  | Cytip |
|  | Ifitm6 |
|  | Kcnc3 |
|  | Arhgdig |
|  | Atf7ip2 |
|  | Tmsb4x |
|  | Nceh1 |
|  | Ptk2b |
|  | Fmo1 |
|  | Kng2 |
|  | Gpnmb |
|  | Fcgr4 |
|  | L1cam |
|  | Tgfb1 |
|  | Sp100 |
|  | Gcnt1 |
|  | P2rx4 |
|  | St3gal5 |
|  | Slc37a1 |
|  | Metrnl |
|  | Gbp8 |
|  | Olfr1033 |
|  | H2-T-ps |
|  | Pilrb2 |
|  | Ppm1h |
|  | Sp140 |
|  | Gdf6 |
|  | Slc22a4 |
|  | Ifi213 |
|  | Ptpre |
|  | Gm4788 |
|  | Rasgrp4 |
|  | Mmp8 |
|  | Naip2 |
|  | Evi2b |
|  | Frzb |
|  | Cerk |
|  | Tmem160 |
|  | Blvra |
|  | Slfn1 |
|  | Xylt2 |
|  | Kctd12 |
|  | Lpin2 |
|  | Slco4a1 |
|  | Tmem114 |
|  | Podnl1 |
|  | Pappa2 |
|  | Tmem189 |
|  | Neil2 |
|  | Tnfrsf1b |
|  | Rnf128 |
|  | 0610040J01Rik |
|  | Rnaset2b |
|  | Rasgrp2 |
|  | Pilra |
|  | Clec10a |
|  | Gusb |
|  | Lipa |
|  | Klhdc7a |
|  | Adssl1 |
|  | Hp |
|  | Zdhhc23 |
|  | Traf3ip3 |
|  | 3830403N18Rik |
|  | Slc28a2b |
|  | Grhl1 |
|  | Gpr27 |
|  | Lonrf3 |
|  | Dnah8 |
|  | Dram1 |
|  | Il4ra |
|  | Cd300ld4 |
|  | Zfp710 |
|  | Arl5c |
|  | Arhgap19 |
|  | Tspan13 |
|  | H2-Ob |
|  | Sdcbp |
|  | Tnfsf9 |
|  | Pctp |
|  | Cox4i2 |
|  | Cfap53 |
|  | Bmx |
|  | Il10 |
|  | Gm8909 |
|  | Ttc7 |
|  | Sdf2l1 |
|  | H2-Q4 |
|  | Ifi27l2a |
|  | Aldh3b1 |
|  | Sell |
|  | Gfra4 |
|  | Tor4a |
|  | Serping1 |
|  | Ccl8 |
|  | Rassf4 |
|  | Ctsz |
|  | Trim30c |
|  | Gna15 |
|  | Mitf |
|  | Fgd4 |
|  | Rerg |
|  | Rbpms |
|  | Fkbp1b |
|  | Pnpla7 |
|  | Il1rn |
|  | Rhog |
|  | Osbpl8 |
|  | Ifi47 |
|  | Clec7a |
|  | Rbp4 |
|  | Slc31a2 |
|  | P2ry2 |
|  | Rab8b |
|  | Pvrig |
|  | Adra1a |
|  | Tnfrsf13b |
|  | Ogfrl1 |
|  | Ticam2 |
|  | Prl2c2 |
|  | Abhd12 |
|  | Scel |
|  | St18 |
|  | Ap1b1 |
|  | LOC100041057 |
|  | Ppfibp2 |
|  | Rgs10 |
|  | Sgsh |
|  | Ero1b |
|  | Fgf2 |
|  | Des |
|  | Nfkbia |
|  | Madd |
|  | Itga11 |
|  | Nfkbie |
|  | Ankrd66 |
|  | H2-M2 |
|  | Havcr2 |
|  | Ccdc40 |
|  | Saa3 |
|  | Gm2619 |
|  | Rnf17 |
|  | Gm33373 |
|  | Vcam1 |
|  | Stx7 |
|  | Ehd4 |
|  | Oasl1 |
|  | Nat8l |
|  | Padi4 |
|  | C2 |
|  | Aoah |
|  | Mfsd12 |
|  | Cfd |
|  | Mertk |
|  | Fcrls |
|  | Lfng |
|  | Tlr9 |
|  | Acss1 |
|  | Sdc3 |
|  | Ppt2 |
|  | Atp13a2 |
|  | Ehhadh |
|  | Plcl2 |
|  | Gprin3 |
|  | Tspan18 |
|  | Gm13212 |
|  | Cd55b |
|  | Grk2 |
|  | Ifi209 |
|  | Gpr146 |
|  | Scamp5 |
|  | Pgm2l1 |
|  | Psme2b |
|  | Ajm1 |
|  | Il18 |
|  | Heph |
|  | Slc9a7 |
|  | Aldh3a1 |
|  | A530032D15Rik |
|  | Gab2 |
|  | Atp2a3 |
|  | Zbtb8b |
|  | Sema4a |
|  | Csmd1 |
|  | Slc22a18 |
|  | Stra6l |
|  | Stard3 |
|  | Zdhhc14 |
|  | Kctd12b |
|  | Mefv |
|  | Cryl1 |
|  | Zranb3 |
|  | Slamf7 |
|  | Slc12a7 |
|  | Syt8 |
|  | Cmtm5 |
|  | Dgkz |
|  | Fgf9 |
|  | Cyp4v3 |
|  | Prdx5 |
|  | LOC100503923 |
|  | Hey1 |
|  | Ago4 |
|  | Dlx3 |
|  | Crlf3 |
|  | Ankdd1b |
|  | Mansc1 |
|  | Tspyl3 |
|  | Akr1c13 |
|  | Gm36079 |
|  | Dmrt2 |
|  | Icam2 |
|  | Fhad1 |
|  | Serpinb9b |
|  | Epor |
|  | Micall2 |
|  | Arhgdib |
|  | Arhgef6 |
|  | Tcp11x2 |
|  | Ecm1 |
|  | Clec4a1 |
|  | Dnase2a |
|  | Pth1r |
|  | F830045P16Rik |
|  | Ceacam16 |
|  | Atp6v0a1 |
|  | Rasgrp3 |
|  | Tnfsf15 |
|  | Zc3h12a |
|  | Lrp3 |
|  | Sec11c |
|  | Traf1 |
|  | Serpinb6b |
|  | Pgd |
|  | Cdk18 |
|  | Cd300e |
|  | Htatip2 |
|  | Tnfrsf8 |
|  | Rnf207 |
|  | Cyp4f16 |
|  | Rap2b |
|  | Pik3cd |
|  | Tmem268 |
|  | Cdkn2a |
|  | Cd274 |
|  | Aldoc |
|  | Ifi206 |
|  | Fkbp15 |
|  | Pdxk |
|  | Cd302 |
|  | Mrgpre |
|  | Ppfia4 |
|  | Sucnr1 |
|  | Pon2 |
|  | Wdr91 |
|  | Cmbl |
|  | 9930111J21Rik1 |
|  | Mafb |
|  | Tnni2 |
|  | Xdh |
|  | Il17ra |
|  | Ramp1 |
|  | Gm52768 |
|  | F5 |
|  | Fam222a |
|  | Gzme |
|  | Wwp1 |
|  | AI661453 |
|  | Cyth1 |
|  | Akap3 |
|  | Plekhm1 |
|  | Tcirg1 |
|  | P2ry13 |
|  | Slc9a3r1 |
|  | Arpc3 |
|  | Olfr99 |
|  | Cyp2r1 |
|  | Slamf8 |
|  | Ssh2 |
|  | 4933430I17Rik |
|  | Mapk13 |
|  | Capg |
|  | Cbln1 |
|  | Jph2 |
|  | Gm15448 |
|  | Nrp2 |
|  | Gmip |
|  | Calcb |
|  | Parp14 |
|  | Pram1 |
|  | Crct1 |
|  | Gas6 |
|  | Rgs9 |
|  | M6pr |
|  | Endod1 |
|  | Tnf |
|  | Ly86 |
|  | Il13ra2 |
|  | Map3k7cl |
|  | Cdk6 |
|  | Hexa |
|  | Aph1c |
|  | Tpd52 |
|  | Kcnj16 |
|  | Tfpi2 |
|  | BC049715 |
|  | Tgm1 |
|  | Susd1 |
|  | Lce1g |
|  | BC147527 |
|  | Il15 |
|  | Grid1 |
|  | Lrp12 |
|  | Slc17a9 |
|  | Adra2a |
|  | Tspan14 |
|  | Gpr18 |
|  | Pcdha8 |
|  | Nrcam |
|  | 9930111J21Rik2 |
|  | Skap2 |
|  | C3 |
|  | LOC546061 |
|  | Gng2 |
|  | Ralgapa2 |
|  | Acox3 |
|  | Smim38 |
|  | Zfp972 |
|  | Ifi27l2b |
|  | Tnip1 |
|  | Tlr6 |
|  | Draxin |
|  | Best1 |
|  | Gm52795 |
|  | Rarres2 |
|  | Syt5 |
|  | Ifi207 |
|  | Gla |
|  | Ldlrap1 |
|  | Car5b |
|  | Csf2ra |
|  | Cd86 |
|  | Armc3 |
|  | Cxcl2 |
|  | LOC101055672 |
|  | Slc8b1 |
|  | Slc24a5 |
|  | Plin1 |
|  | Nrap |
|  | Uba7 |
|  | Slc48a1 |
|  | Epb41l4a |
|  | Ubl3 |
|  | Pcdhga5 |
|  | Rtn4rl2 |
|  | Otor |
|  | Tjp3 |
|  | Sephs2 |
|  | Wfdc1 |
|  | Asic3 |
|  | Pigz |
|  | Ccr2 |
|  | Chn2 |
|  | Dusp3 |
|  | Wfdc18 |
|  | Cyp11b1 |
|  | Plscr4 |
|  | Atp6v1b2 |
|  | Tbx2 |
|  | Slc35f6 |
|  | Cd300ld5 |
|  | Trim30b |
|  | Map3k11 |
|  | Asb10 |
|  | Erbb3 |
|  | Ache |
|  | Plekhm3 |
|  | Sez6l2 |
|  | Syn1 |
|  | Gch1 |
|  | Colq |
|  | Sirpb1c |
|  | Bcl6b |
|  | Cfhr2 |
|  | Exoc6 |
|  | Prkn |
|  | Hvcn1 |
|  | Trem3 |
|  | Mctp1 |
|  | Irak2 |
|  | Fhl5 |
|  | Snx8 |
|  | Htr2b |
|  | Mgst1 |
|  | Tbc1d30 |
|  | Fgl2 |
|  | Cd79b |
|  | Plbd1 |
|  | Tmem82 |
|  | Pip4k2a |
|  | Adrb2 |
|  | Kcnu1 |
|  | Pla1a |
|  | Fam20c |
|  | Cdc42se2 |
|  | Elmo1 |
|  | Hgf |
|  | Glul |
|  | Pacc1 |
|  | Il6 |
|  | Tmc6 |
|  | Nostrin |
|  | Kcnj2 |
|  | Cmtm6 |
|  | Slfn3 |
|  | Sfxn5 |
|  | LOC100039029 |
|  | Gm29825 |
|  | Kif19a |
|  | Plin2 |
|  | Slamf6 |
|  | Por |
|  | Gab3 |
|  | Qpct |
|  | Adgrl4 |
|  | Gm5127 |
|  | Itga9 |
|  | Cfap47 |
|  | Gzmc |
|  | Slit1 |
|  | Ephx2 |
|  | Lbp |
|  | Fam167a |
|  | Rab39 |
|  | Slc12a1 |
|  | Ctla2b |
|  | Psen2 |
|  | Oas2 |
|  | Ly6k |
|  | Cyrib |
|  | Gdf3 |
|  | Tm4sf19 |
|  | Slc43a3 |
|  | Zfp982 |
|  | Atf3 |
|  | BC049352 |
|  | Rgs20 |
|  | Selenbp2 |
|  | Pcdha9 |
|  | Phf11a |
|  | Fpr2 |
|  | Nat8f3 |
|  | Tmod1 |
|  | Tlcd2 |
|  | 1700001C19Rik |
|  | Klra17 |
|  | Fth1 |
|  | Fgd3 |
|  | Zfyve28 |
|  | Sphk2 |
|  | Dusp1 |
|  | Rarres1 |
|  | Slc2a9 |
|  | Emcn |
|  | Kcne3 |
|  | Phf11b |
|  | Bcl3 |
|  | Mak |
|  | Neu1 |
|  | Slc16a9 |
|  | Ccn5 |
|  | Dok5 |
|  | Gm35498 |
|  | Rnasel |
|  | Pnp2 |
|  | Ptger4 |
|  | Fli1 |
|  | Olfr111 |
|  | F10 |
|  | Grn |
|  | Fuca2 |
|  | Gstt3 |
|  | Sod2 |
|  | Cds1 |
|  | Cd101 |
|  | 1700010I14Rik |
|  | Gm42517 |
|  | Gm38525 |
|  | Tnfaip2 |
|  | Rnase2b |
|  | Pld3 |
|  | Slc16a10 |
|  | LOC115489401 |
|  | Acod1 |
|  | Nr3c2 |
|  | St3gal6 |
|  | Phyhipl |
|  | Radx |
|  | Zfp534 |
|  | Klra9 |
|  | Syk |
|  | Slc7a4 |
|  | Slc6a6 |
|  | Slc16a13 |
|  | Skint3 |
|  | Msx2 |
|  | N4bp2l1 |
|  | Tmem37 |
|  | F9 |
|  | Slc26a7 |
|  | Zbp1 |
|  | Fam124a |
|  | Ptpn22 |
|  | Slc13a5 |
|  | Atp8a2 |
|  | Als2cl |
|  | Ptprd |
|  | 1700001J03Rik |
|  | Gsap |
|  | H2-T10 |
|  | Arhgap6 |
|  | Ceacam1 |
|  | Myl2 |
|  | H2-Q10 |
|  | Lce1h |
|  | Cln8 |
|  | Gpr160 |
|  | Atp9a |
|  | Nlrp10 |
|  | St14 |
|  | 2310030G06Rik |
|  | Pdzk1ip1 |
|  | Stmn2 |
|  | Ap5b1 |
|  | Bcl2a1a |
|  | Slc25a21 |
|  | Gm2427 |
|  | Mob3b |
|  | Jaml |
|  | Gclm |
|  | Klra3 |
|  | Gsr |
|  | Marco |
|  | Shank3 |
|  | Grpr |
|  | Myo18b |
|  | S100a8 |
|  | Shpk |
|  | LOC108167387 |
|  | Fabp4 |
|  | Ifi30 |
|  | Dpysl4 |
|  | Arfgef3 |
|  | Urah |
|  | Epop |
|  | Rgs11 |
|  | Gdpd5 |
|  | Tmem176a |
|  | Slc27a1 |
|  | Gpr31b |
|  | Coro7 |
|  | Acsl1 |
|  | Ftl1 |
|  | Tnfrsf21 |
|  | Psmb10 |
|  | Prss23 |
|  | Cox6b2 |
|  | Mst1 |
|  | Serpinb12 |
|  | Cntn2 |
|  | Cyba |
|  | Ldb3 |
|  | Ct55 |
|  | Rnf144b |
|  | Vipr1 |
|  | Ly75 |
|  | Slc1a1 |
|  | Tecpr1 |
|  | Nod2 |
|  | Nlrc5 |
|  | Gm46894 |
|  | Zfand2a |
|  | Atp6v0c |
|  | Gapt |
|  | Sdsl |
|  | Esm1 |
|  | H2-T24 |
|  | Cxcl9 |
|  | Muc16 |
|  | Hip1 |
|  | Dlx4 |
|  | Slc16a6 |
|  | Tmem150b |
|  | Apoc1 |
|  | Paqr7 |
|  | Lrch4 |
|  | Rhov |
|  | Gm46272 |
|  | Dapk1 |
|  | Glrx |
|  | Cck |
|  | Rbm47 |
|  | Slc36a2 |
|  | Maneal |
|  | Emilin2 |
|  | LOC100041708 |
|  | Prl2c5 |
|  | Dpep1 |
|  | Arhgap15 |
|  | Sdr42e1 |
|  | 1500009L16Rik |
|  | Tspan7 |
|  | Rgs17 |
|  | Pecam1 |
|  | Dtx1 |
|  | Fbxo16 |
|  | Lhpp |
|  | Tnfaip8 |
|  | Foxd1 |
|  | Ascl2 |
|  | Fam83f |
|  | Trim65 |
|  | Tifa |
|  | Cxcl13 |
|  | Asb4 |
|  | Gzmd |
|  | Tmem154 |
|  | Gmfg-ps |
|  | Gm52481 |
|  | Bhlhe41 |
|  | Gml |
|  | Sema6d |
|  | Lrrc3 |
|  | Selenbp1 |
|  | Gm11545 |
|  | Pstpip2 |
|  | Sncg |
|  | Creb5 |
|  | Slc40a1 |
|  | Gm5150 |
|  | Tmem176b |
|  | Epn3 |
|  | Ccdc194 |
|  | LOC115489311 |
|  | Slfn8 |
|  | Pnpla2 |
|  | Cxcl1 |
|  | Ralyl |
|  | Gbgt1 |
|  | Shisa2 |
|  | Casp7 |
|  | Lratd2 |
|  | Hfe |
|  | Engase |
|  | Ankrd1 |
|  | Camk4 |
|  | Esrp2 |
|  | Cdh5 |
