# Supplementary material for: Melatonin enhances osteoblastogenesis of senescent bone marrow stromal cells through NSD2‐mediated chromatin remodelling
Source: Clin Transl Med. 2022 Feb 27;12(2):e746. doi: 10.1002/ctm2.746 (PMC8882236; doi:10.1002/ctm2.746)
Supplement: Supplementary file 2 — Table S2 [file CTM2-12-e746-s003.docx]

**Table S 2. Aged mouse BMSCs treated with melatonin versus vehicle.**

| **Down genes** | **Up genes** |
| --- | --- |
| Mmp13 | Tenm3 |
| Myom1 | Pcdh10 |
| Cfh | Stc2 |
| Sp7 | Hopx |
| Glipr1 | Crabp1 |
| Loxl2 | Cthrc1 |
| Pnp2 | Asxl3 |
| B3gnt5 | Fbn2 |
| Efnb1 | Scn3a |
| Ackr4 | Flrt3 |
| Clec4a1 | Il33 |
| Bcam | Penk |
| Slc28a2 | Robo2 |
| Tgtp2 | Pear1 |
| Gbp5 | Capn6 |
| Cd36 | Slc38a4 |
| Asb2 | Slc5a7 |
| Trf | Epha7 |
| Itgal | Egfl6 |
| Il2rg | Plagl1 |
| Slc7a7 | Adgrl3 |
| Rassf3 | Psip1 |
| Cd300ld | Tcf4 |
| Slc15a3 | Msln |
| Nlrp3 | Neto2 |
| Fpr1 | Tgif2 |
| Pilrb2 | Fam180a |
| Cd300e | Elavl2 |
| Itgam | Aspn |
| Ncf4 | Mex3a |
| Prxl2b | Scx |
| Folr2 | Unc5c |
| Alpl | H1f10 |
| Aif1 | Aadat |
| Slc25a45 | Pdgfrl |
| Pilra | D630045J12Rik |
| Fcgr4 | Il17rd |
| Cxcl16 | Hivep2 |
| Hck | Nptx1 |
| Siglece | Tcim |
| H2-Ab1 | Klf5 |
| Mrap | Abi3bp |
| Tnip3 | Frmpd4 |
| AB124611 | F2rl1 |
| Esm1 | Htr7 |
| B430306N03Rik | Gem |
| Ret | Nova1 |
| Barx1 | Arhgap32 |
| Casp1 | Apod |
| Aoah | Nrk |
| Acp5 | Meox2 |
| Adgb | Mfap4 |
| Fcgr1 | Rab39b |
| Slc11a1 | Ano5 |
| Fcna | Sema3a |
| Gda | Tmem74 |
| Slc16a7 | Adm2 |
| Clec12a | Trim66 |
| Adgrf5 | Slitrk1 |
| Blnk | Phgdh |
| Clec4a2 | Bdh2 |
| Ccl9 | Fbln1 |
| Angpt2 | Cdh24 |
| Ampd3 | Cdkn1c |
| Clec4e | Ankrd34a |
| Msr1 | Erg |
| Pf4 | Ldb2 |
| Alox5ap | Sec16b |
| Fpr2 | Gabbr1 |
| Pilrb1 | Eln |
| Adgre1 | Ncald |
| Slamf7 | Lamc1 |
| Cd72 | Ppm1l |
| Gpr65 | Dennd3 |
| Fyb | Garem1 |
| Rac2 | Arhgap33 |
| Tnf | Muc1 |
| Lrrc25 | Syde2 |
| Csf1r | Coro2b |
| Sirpb1c | Xpnpep2 |
| Ptpn6 | Dchs1 |
| LOC115485726 | Septin6 |
| Cadm1 | Pcsk4 |
| Ly86 | Nrg1 |
| Hcls1 | Itgb3 |
| Ctsc | Sfrp2 |
| C5ar1 | Psat1 |
| H2-DMb1 | Ccdc3 |
| Abhd15 | Lrfn4 |
| Scarf1 | Dennd11 |
| Trem1 | Zfp57 |
| Synpo2 | Tnmd |
| Gm38510 | Kcnma1 |
| Clec4a3 | Ptchd1 |
| Cd300ld3 | Dnm3 |
| Gm35498 | Sulf1 |
| Chst8 | Zfp9 |
| Myo1g | Elovl6 |
| Prdx6 | Fjx1 |
| Hk3 | Rgs6 |
| Abcc3 | Tmod2 |
| Was | Mtcl1 |
| Fermt3 | Fgf21 |
| Slamf9 | Tbx18 |
| Tmem273 | Gli3 |
| A530064D06Rik | Ramp3 |
| Cd48 | Adh7 |
| Dpep2 | Prickle2 |
| Ikzf1 | Zfhx4 |
| Hhex | Sema6a |
| Rapgef5 | Igf2bp2 |
| Cd52 | Rnf165 |
| Mmp3 | Gcnt4 |
| 2200002D01Rik | Pi15 |
| Adcyap1r1 | Aldh1l2 |
| Gm21188 | Arl5b |
| Mmp8 | Tspan15 |
| Kng1 | Map2 |
| Smagp | Cdon |
| Ncf1 | Tnfaip6 |
| Tnfrsf11a | Cth |
| Selplg | Rhobtb3 |
| Vav1 | Pgm5 |
| Lrmda | Adamts4 |
| Cd300lf | Srd5a1 |
| Lcp1 | Slc6a9 |
| Cybb | Rnf43 |
| Tm6sf1 | Akr1c14 |
| Cfp | Pak3 |
| Prkch | Dpyd |
| Adipoq | Xkr5 |
| Ccr5 | Nhsl1 |
| Ugt1a7c | Lratd1 |
| Bin2 | Prdm8 |
| Sorbs2 | Gpr62 |
| Arhgap4 | Slit2 |
| C1qb | Utp14b |
| Marchf1 | Arhgap20 |
| Cyth4 | Magi3 |
| Slc13a3 | Cftr |
| Gm5150 | Chac1 |
| C1qa | Il1rapl1 |
| Trpv2 | Macrod2 |
| Lpxn | Auts2 |
| Pygl | Cyp2c23 |
| Trpm2 | Zfp827 |
| Themis2 | Bnc2 |
| Kcnk13 | Cd34 |
| Bst1 | Adamts20 |
| Lrmp | Apold1 |
| Shtn1 | Zfp599 |
| Tbxas1 | Zc3hav1l |
| Dok2 | Kcnd2 |
| Adam8 | Hlf |
| Mmp27 | Cavin4 |
| Tmem106a | Tram1l1 |
| Prss16 | Fzd6 |
| Ms4a6d | Slc7a5 |
| Irf8 | Gucy1a2 |
| Gm12250 | Rassf8 |
| Gpx3 | Flt1 |
| Dock2 | Meox1 |
| S100b | Phex |
| Slco4a1 | Prx |
| Sirpb1b | Mboat1 |
| Ncf2 | Pdzd7 |
| Adrb1 | Eomes |
| Lcp2 | Gxylt2 |
| Mylk | Ccn3 |
| Fcer1g | Nrip2 |
| Spi1 | A830018L16Rik |
| Slfn10-ps | Ddit3 |
| Ebi3 | Nr4a2 |
| Tcp11x2 | Aldh18a1 |
| P2ry6 | Rpp25 |
| Nfam1 | Gfra2 |
| Sla | Trib3 |
| Nfatc2 | Six4 |
| Tyrobp | Tmem169 |
| C1qc | Gpt2 |
| Mpeg1 | Rgma |
| Dennd1c | Serinc2 |
| Abcg1 | Mtbp |
| Pira2 | Myom2 |
| Itgb2 | Slc1a3 |
| Pycard | Nsd2 |
| Lilra5 | Plxna3 |
| Cd244a | Col3a1 |
| Cd38 | Setbp1 |
| Stxbp2 | Itih2 |
| Arhgap9 | Lvrn |
| Slfn5 | Sorcs2 |
| Ms4a6c | Ptn |
| Gpsm3 | Npas2 |
| Icam1 | Cspg4 |
| Gm14548 | Shroom2 |
| Avpr1a | Ank2 |
| Gm1966 | Wls |
| Tlr13 | Zfp184 |
| Arhgap30 | Mgp |
| P2ry14 | Adgra2 |
| Ica1l | Klf12 |
| Tlr1 | Slc25a27 |
| Mtarc1 | Lgi2 |
| Igsf6 | Pard6b |
| Snx20 | Fndc4 |
| Rinl | Plk2 |
| Ptpn18 | Wasf1 |
| Ccl6 | Gfod1 |
| Clec5a | Kcnip1 |
| Tmem221 | Cdkn1b |
| Slc7a8 | Tbx3 |
| Ctss | Acsl3 |
| Siglec1 | Galnt5 |
| Atp8b4 | Tet1 |
| Vsig4 | Gramd4 |
| Mmp12 | Ndst4 |
| Slc43a2 | Itga2b |
| Aldh2 | Rnf122 |
| Epsti1 | Cdkl5 |
| Rnase6 | 4930447C04Rik |
| Rgs1 | Fgd1 |
| Tlr8 | Tlcd4 |
| Batf2 | Gng13 |
| Klhl6 | Cachd1 |
| Prkcb | Asns |
| LOC100041057 | Myh10 |
| Neurl3 | Tbx4 |
| Lyn | Uck2 |
| Cd59a | Epha4 |
| Sh3tc1 | AW551984 |
| H2-Q7 | Srgap1 |
| Ankrd33b | Podn |
| Csf2rb | Zeb1 |
| Gpr84 | Lrig3 |
| Csprs | F11r |
| Arap3 | Met |
| Acod1 | Sox12 |
| Cd68 | Myzap |
| Trarg1 | Eps8l3 |
| Ccdc88b | Pycr1 |
| Tnfrsf13b | Col12a1 |
| Cd55 | Syt11 |
| Bcl2a1b | Sema3f |
| Nr1h3 | Chst10 |
| Rtn4rl1 | Bmpr1b |
| Gvin1 | Pwwp3b |
| Apobec1 | Ttbk1 |
| Cox4i2 | Tmtc2 |
| C130026I21Rik | Inka1 |
| Ehd3 | Ltbp4 |
| Nckap1l | Cldn34c1 |
| Lst1 | P2rx3 |
| Trem2 | Igsf10 |
| Ccl12 | Prrt2 |
| Arl11 | Col11a1 |
| Coro1a | Lingo4 |
| Pik3r5 | Pde9a |
| Cd180 | P2rx5 |
| Stap1 | Gls2 |
| Pirb | Pcdhb20 |
| Frmd4b | Bcat1 |
| Galnt6 | Sh3rf3 |
| Samsn1 | Nfix |
| Ly9 | Hhip |
| Apoe | Brinp1 |
| Arhgap25 | Gask1a |
| Clec4d | Bmf |
| Trpv4 | Cmya5 |
| Otulinl | Mapk10 |
| Cebpa | Pcdhb18 |
| Saa3 | Fndc1 |
| Lair1 | Ostn |
| Mmp9 | Lix1l |
| Ckb | Itga7 |
| Pld4 | Ptprq |
| Fgd2 | Strbp |
| Acvrl1 | Atp6v0a4 |
| Lmo2 | Tox |
| Btk | Ctdsp2 |
| Cacna1d | Rev3l |
| Cd53 | Runx2 |
| H2-Q6 | Angptl6 |
| Lyz2 | Acot2 |
| Dock8 | Hoxd4 |
| Clec7a | Lpar4 |
| Milr1 | Hmgb2 |
| Vsir | Dock9 |
| Fmnl1 | Hid1 |
| Slc39a4 | Hoxd9 |
| F13a1 | Lyplal1 |
| Tifab | Mmp14 |
| Il10ra | Lmx1b |
| Lgals3 | Daam2 |
| Cd207 | Peg10 |
| Crybg1 | Ndst3 |
| Akr1c12 | En1 |
| Nectin4 | Cacna1c |
| Myo1f | Ddah2 |
| Cyfip2 | Tfap4 |
| Arrb2 | Rcc2 |
| Arhgap45 | Pcdhga3 |
| Ms4a6b | Spock2 |
| Cd300c2 | Nlrp2 |
| Ifi204 | Slitrk6 |
| Inpp4b | Atp1b1 |
| Pla2r1 | Cdh3 |
| Pou2f2 | Olfml1 |
| Abcb4 | Spats2l |
| Gm7609 | Car6 |
| Oas3 | Steap4 |
| Laptm5 | Ephb3 |
| Fcgr3 | Nedd9 |
| Tlr7 | C1qtnf1 |
| Xlr | Avil |
| Coro2a | H1f0 |
| Ccl3 | Acot1 |
| Iqgap2 | Ovgp1 |
| Csf2rb2 | Cryz |
| Ptprc | Sertad4 |
| Sash3 | Gpr88 |
| Epas1 | Cd109 |
| Hpgds | Ephb6 |
| Wfdc17 | Zfp395 |
| Csmd1 | Epha3 |
| Isyna1 | Zfpm2 |
| Srgn | Depdc1a |
| Cd86 | Ncam2 |
| Dok3 | Pck2 |
| Nlrp1b | Gpr153 |
| Oas1a | Cacnb4 |
| Irf5 | Lhx9 |
| Crispld2 | Kcnc2 |
| Grap | Thbs4 |
| Ccr1 | Bend5 |
| Pik3cg | Nfatc4 |
| Crip1 | Pxdn |
| Rcsd1 | Lrrtm3 |
| Apoc2 | Eps8 |
| Mbp | Tbx20 |
| Fabp7 | Gpsm2 |
| Cysltr1 | Ppargc1a |
| Tnfaip3 | Opn1sw |
| Hnmt | Plekha5 |
| Oas1g | Tex52 |
| Kit | Slc16a1 |
| Prex1 | Ccdc136 |
| Tbc1d30 | Sema3c |
| Il18bp | Skida1 |
| Lyz1 | Tspyl5 |
| Pik3ap1 | Plat |
| Ptpro | Pknox2 |
| Spn | Col1a2 |
| Angpt4 | Osbpl6 |
| Ucp2 | Mfap2 |
| Chil3 | Ssbp2 |
| Tnfaip8l2 | Wscd2 |
| Rgs14 | Prtg |
| Sirpa | Atrnl1 |
| 5430427O19Rik | Fv1 |
| Wdfy4 | Cd276 |
| Cd40 | Ypel4 |
| Cxcr3 | Uty |
| Cd37 | Gnat2 |
| Sp110 | Adcy1 |
| Pde1b | Fbxo2 |
| Aatk | Adamts6 |
| Rasgef1b | Efna1 |
| Inpp5d | Kcna4 |
| Sfrp4 | Fgf5 |
| Apol6 | Apcdd1 |
| Psmb8 | Zfp521 |
| Cxcl14 | Slc26a10 |
| Ric3 | Adh1 |
| Malrd1 | Apln |
| Adap1 | Cyp7b1 |
| C3ar1 | Lag3 |
| Tmem171 | Klhl29 |
| Fbxl2 | Rflna |
| Efhd2 | Tcea3 |
| Smpdl3a | Scarf2 |
| Cd84 | B3gnt9 |
| Il21r | Sesn3 |
| Fap | Inava |
| Cd93 | Crispld1 |
| Card9 | Zcchc3 |
| Stab1 | Lrrtm1 |
| Pkib | Pdzrn3 |
| Fes | Glce |
| Tmem141 | Zfp882 |
| Aim2 | Mboat2 |
| Susd3 | Atp8b1 |
| Tmem140 | Klhl32 |
| Gm4070 | Kcnab1 |
| Nxpe5 | Shisa8 |
| Cd200r1 | Kif21a |
| Adap2 | Card10 |
| Pdgfb | Car10 |
| Tlcd2 | Hmcn1 |
| Tfec | Zfp354c |
| Cd300lb | B4galnt4 |
| Podnl1 | Fzd2 |
| Gatm | Gulp1 |
| Psma8 | Egfl7 |
| Clec4n | Mtmr11 |
| Grk3 | Ect2 |
| Fcgr2b | Fat4 |
| C5ar2 | Gm5796 |
| Gpr141 | Catsperd |
| Rps6ka1 | Tgm3 |
| Lpar5 | Rab27b |
| Unc13d | Pitx1 |
| Cotl1 | Mrgprf |
| I830077J02Rik | Traip |
| Gdf15 | Faim2 |
| Adcy5 | Ces1a |
| Plcb2 | Clec3b |
| Il10 | Mcc |
| Rasal3 | Slc2a2 |
| Csf3r | Hnrnpa1l2-ps2 |
| Tnfsf13b | Fibin |
| Bank1 | Cts8 |
| Map3k9 | Pcsk1 |
| Dmpk | Cspg4b |
| Sema6b | Foxh1 |
| Rnaset2b | Ube2ql1 |
| Unc5a | Hoxd8 |
| Zfp979 | Itgb8 |
| Lilrb4a | Thbs3 |
| Rab32 | Gprc5a |
| Calcrl | Bicd1 |
| Pla2g7 | Gcat |
| Zmynd15 | 3110009E18Rik |
| Aldh3a1 | Cend1 |
| Cd22 | Postn |
| Cndp2 | Col16a1 |
| Gmfg | Xkr6 |
| Tmem71 | Hspa12a |
| Ptprj | Cfap20dc |
| Blvrb | Rasl11b |
| Prr13 | Tgfbr3l |
| Ms4a4a | Pnldc1 |
| Unc93b1 | Unc13c |
| Dock10 | Slc5a5 |
| Parvg | Spink10 |
| Cyp4f18 | Lpar2 |
| LOC102638047 | Ssc5d |
| Ifitm6 | Spta1 |
| Cela1 | Pcdhgb6 |
| Ms4a7 | Masp2 |
| Tmem104 | Cand2 |
| Tmem51 | Clip4 |
| Map3k5 | Hs3st3b1 |
| Cd200r4 | Pcolce |
| H2-D1 | Nckap5 |
| B3gnt8 | Egf |
| Il16 | Edil3 |
| Ehhadh | Twist1 |
| Mob3b | Rhd |
| Gdf3 | Gm2237 |
| Ccl2 | Col27a1 |
| Gdf10 | Map2k6 |
| Naip5 | Jcad |
| Fam53b | Pkhd1l1 |
| Cln3 | Mfsd2a |
| Ticam2 | Ube2cbp |
| Sp100 | Pcdh7 |
| Lat2 | Slco5a1 |
| Creg1 | Gli1 |
| Hp | Ociad2 |
| Gpr18 | Acvr2a |
| Psd4 | Pde5a |
| Abr | LOC115486128 |
| Sh3bp2 | LOC105244150 |
| Pgap6 | Gm46353 |
| Gm5431 | Gm42346 |
| Klra2 | Gm42049 |
| Pira1 | Gm4120 |
| H2-K1 | Gm40848 |
| Ccr3 | Gm40363 |
| Kif19a | Gm3973 |
| Mgat4a | Gm38699 |
| Tnn | Gm38690 |
| Mag | Gm3636 |
| Atp8a1 | Gm32719 |
| Pparg | Gm31513 |
| Ccl4 | Gm21297 |
| Spon1 | Gm17330 |
| Eif4e3 | Gm15246 |
| Ifi213 | Gm10265 |
| Pvrig | D830031N03Rik |
| Rnf149 | Duxbl3 |
| Ifi47 | Eppk1 |
| S100a1 |  |
| Sell |  |
| Napsa |  |
| Ch25h |  |
| Des |  |
| Igf1 |  |
| Heph |  |
| Fgf13 |  |
| Rab19 |  |
| Orai2 |  |
| Thsd4 |  |
| Tmem229b |  |
| Il10rb |  |
| Traf3ip3 |  |
| Atp1a3 |  |
| Stard8 |  |
| Ecscr |  |
| Rab39 |  |
| Sh3bgr |  |
| Fxyd5 |  |
| Lrp4 |  |
| S1pr1 |  |
| Pik3r6 |  |
| Il7r |  |
| Xlr3b |  |
| Sgk3 |  |
| Lilr4b |  |
| Slc46a3 |  |
| P2ry13 |  |
| Fcrl1 |  |
| Hgsnat |  |
| Inpp5j |  |
| Cnr2 |  |
| Ifi206 |  |
| Lpl |  |
| Lpcat2 |  |
| Adra1a |  |
| Gapt |  |
| Zeb2 |  |
| Arl5c |  |
| LOC100862446 |  |
| Ccrl2 |  |
| Pnpo |  |
| Susd5 |  |
| Ccl7 |  |
| Tnfsf13 |  |
| Rhbdf2 |  |
| Mcoln2 |  |
| Msrb1 |  |
| Pigz |  |
| Itga11 |  |
| Arhgap27 |  |
| Fut7 |  |
| Serping1 |  |
| Cd14 |  |
| Sp140 |  |
| Psmb9 |  |
| Pcdhga10 |  |
| Slc22a4 |  |
| Nlrp1a |  |
| Renbp |  |
| Ptafr |  |
| Nlrc4 |  |
| St18 |  |
| Jph2 |  |
| Rab7b |  |
| Cd5l |  |
| Zc3h12d |  |
| Gm7592 |  |
| Cdkn1a |  |
| H2-Q4 |  |
| Cd300a |  |
| Slc1a1 |  |
| Pon3 |  |
| Plek |  |
| Mfsd6 |  |
| Efs |  |
| Slfn4 |  |
| Aqp1 |  |
| P2ry2 |  |
| Dhrs3 |  |
| A630001G21Rik |  |
| Slc37a1 |  |
| Cysltr2 |  |
| Mefv |  |
| Adra2a |  |
| Klra3 |  |
| Plcg2 |  |
| Mrc1 |  |
| Armc3 |  |
| Hcst |  |
| Frzb |  |
| Arrb1 |  |
| Bco2 |  |
| Slfn1 |  |
| P2ry12 |  |
| Epb41l4a |  |
| Kcnab2 |  |
| Hcar2 |  |
| Apba1 |  |
| Id2 |  |
| AI467606 |  |
| Col2a1 |  |
| Pstpip1 |  |
| Dnajb13 |  |
| Ms4a14 |  |
| Rarres2 |  |
| Plxnb3 |  |
| 9930111J21Rik2 |  |
| Sema4d |  |
| Ifi27l2a |  |
| Ssh3 |  |
| Rnf130 |  |
| S100a8 |  |
| Tlr9 |  |
| Gde1 |  |
| Aldh3b1 |  |
| Cat |  |
| Npy |  |
| Stac2 |  |
| Map11 |  |
| Arrdc4 |  |
| Cd55b |  |
| Tmem243 |  |
| Sema4a |  |
| Rims3 |  |
| Cd300ld5 |  |
| Tpbgl |  |
| Naip6 |  |
| Pdzk1ip1 |  |
| Mfng |  |
| Dnase1l1 |  |
| Xylt2 |  |
| Gngt2 |  |
| Gna15 |  |
| Vav3 |  |
| Orai1 |  |
| Bcl2a1a |  |
| Akr1c18 |  |
| Fam78a |  |
| Plxdc1 |  |
| Tal1 |  |
| Pctp |  |
| Mgat5 |  |
| Lfng |  |
| Tspan32 |  |
| A530032D15Rik |  |
| Lrg1 |  |
| S100a16 |  |
| Abi3 |  |
| Traf1 |  |
| Rnf17 |  |
| Ecm1 |  |
| 0610040J01Rik |  |
| Gbp8 |  |
| Slc39a12 |  |
| Slfn2 |  |
| H2-M3 |  |
| Kng2 |  |
| Trim30c |  |
| Lilra6 |  |
| Cxcr4 |  |
| Fmo1 |  |
| Ninj1 |  |
| Acp2 |  |
| Ctsd |  |
| Cracr2b |  |
| Tcn2 |  |
| Lrrc27 |  |
| Gm6377 |  |
| Cxcl2 |  |
| Dlx3 |  |
| Il4ra |  |
| Epor |  |
| Tgfb1 |  |
| Itga4 |  |
| Il1a |  |
| Cd33 |  |
| Lyl1 |  |
| Slc13a5 |  |
| Slc37a2 |  |
| Scly |  |
| Batf |  |
| Nrros |  |
| Pid1 |  |
| Ptger2 |  |
| Ikbke |  |
| Zbp1 |  |
| Tjp3 |  |
| Mfsd13a |  |
| Kcnk6 |  |
| Cd163 |  |
| Akirin1 |  |
| Lrp3 |  |
| Oasl1 |  |
| Arg1 |  |
| Nbeal2 |  |
| Mocos |  |
| Exoc3l4 |  |
| Stk17b |  |
| Gcnt1 |  |
| St3gal6 |  |
| Trim29 |  |
| Tnfrsf1b |  |
| Cidec |  |
| St8sia4 |  |
| Smurf2 |  |
| Ppm1h |  |
| Cd274 |  |
| Slc13a2 |  |
| Rnpep |  |
| Sncaip |  |
| Pitpna |  |
| G6pdx |  |
| Ctsb |  |
| Nr3c2 |  |
| Ms4a4c |  |
| Lpin2 |  |
| Afp |  |
| Gsta3 |  |
| Gdf6 |  |
| Flt4 |  |
| Cmklr1 |  |
| Cd69 |  |
| Alox5 |  |
| Plin1 |  |
| Akr1b10 |  |
| Sdc3 |  |
| Rassf4 |  |
| Fah |  |
| Rhog |  |
| Bmp2 |  |
| P2rx4 |  |
| Dhdh |  |
| Tmem26 |  |
| St3gal5 |  |
| Rab20 |  |
| Psme2b |  |
| H2-T10 |  |
| Cds1 |  |
| Entpd1 |  |
| BC049352 |  |
| Ptprd |  |
| Cbln1 |  |
| Best1 |  |
| Taldo1 |  |
| Crlf3 |  |
| Rnf128 |  |
| Itga9 |  |
| Stk10 |  |
| Dnah8 |  |
| Fcrlb |  |
| Slc31a2 |  |
| Lgals9 |  |
| Syt8 |  |
| Maf |  |
| Lgmn |  |
| Arhgap19 |  |
| Atp2a3 |  |
| Casp4 |  |
| Frmd3 |  |
| Icosl |  |
| Wfdc18 |  |
| Ifi209 |  |
| Phf11b |  |
| Nfkbid |  |
| Rasgrp2 |  |
| Icam2 |  |
| Prdm1 |  |
| Kcnc3 |  |
| Tlr2 |  |
| Atp9a |  |
| Il13ra2 |  |
| Rhoh |  |
| Dab2 |  |
| Gab2 |  |
| Zfp710 |  |
| Ripk2 |  |
| Ptk2b |  |
| Tmem189 |  |
| Vnn3 |  |
| Parp14 |  |
| Plekhm1 |  |
| Otud7a |  |
| Pcdha8 |  |
| Susd1 |  |
| Sdf2l1 |  |
| Ptpre |  |
| Fcrls |  |
| Metrnl |  |
| Fos |  |
| Fnd3c2 |  |
| Rbp4 |  |
| 1700010I14Rik |  |
| Syt5 |  |
| Slc28a2b |  |
| Cfd |  |
| Cd151 |  |
| Gm21451 |  |
| Naip2 |  |
| Lacc1 |  |
| Syp |  |
| Fuca1 |  |
| Hmox2 |  |
| Tmsb4x |  |
| Vcam1 |  |
| Ctla2b |  |
| Dio3 |  |
| Pnpla7 |  |
| Rgs18 |  |
| Tfpi2 |  |
| Plcl2 |  |
| Cytip |  |
| Ppfibp2 |  |
| Slc12a7 |  |
| Rasgrp4 |  |
| Grk2 |  |
| Trim5 |  |
| Cerk |  |
| Uba7 |  |
| BC049715 |  |
| Hmox1 |  |
| 3830403N18Rik |  |
| Xlr4b |  |
| Gm38525 |  |
| Rilp |  |
| Gm15448 |  |
| Fgf2 |  |
| Ttc7 |  |
| Il1rn |  |
| LOC100503923 |  |
| Gm52481 |  |
| Gpr137b |  |
| Htr1b |  |
| Tmem156 |  |
| Tgm2 |  |
| Jaml |  |
| F5 |  |
| Slc25a10 |  |
| Rnaset2a |  |
| Sdcbp |  |
| Adssl1 |  |
| Gm4951 |  |
| Atp13a2 |  |
| Ccl8 |  |
| Ak8 |  |
| Tagap |  |
| Tnfsf9 |  |
| Il34 |  |
| Gfra4 |  |
| Grpr |  |
| Nceh1 |  |
| Bdkrb1 |  |
| Mfsd12 |  |
| Hgf |  |
| Cacna1f |  |
| Cmtm5 |  |
| Lhfpl4 |  |
| Rab8b |  |
| Fkbp1b |  |
| Fam222a |  |
| Ogfrl1 |  |
| Blvra |  |
| Jph3 |  |
| Cxcl9 |  |
| Dok7 |  |
| Pecam1 |  |
| Ggt5 |  |
| Rgs17 |  |
| Stx7 |  |
| Casz1 |  |
| Psmb10 |  |
| Engase |  |
| Kcnj10 |  |
| Cxcl13 |  |
| Rap2b |  |
| Card6 |  |
| Nod2 |  |
| Gm8909 |  |
| Tfap2a |  |
| Itm2b |  |
| Sh3bgrl2 |  |
| Olfr1033 |  |
| Pgm2l1 |  |
| Rsad2 |  |
| Wdr91 |  |
| Lce1g |  |
| Pgd |  |
| Zc3h12a |  |
| Tor4a |  |
| L1cam |  |
| Ajm1 |  |
| Gusb |  |
| Lnx1 |  |
| Lrrc3 |  |
| Man2b1 |  |
| Dnmt3l |  |
| Ceacam10 |  |
| BC147527 |  |
| Mtss1 |  |
| Osm |  |
| Tapbpl |  |
| Calhm6 |  |
| Fads6 |  |
| Madd |  |
| Prdx5 |  |
| Csf2ra |  |
| Syn1 |  |
| Tlr6 |  |
| Wwp1 |  |
| Clec10a |  |
| Fgf11 |  |
| Mrgpre |  |
| LOC101055663 |  |
| Pon2 |  |
| Scel |  |
| Kctd12 |  |
| Tnfrsf9 |  |
| Nrg4 |  |
| Bcl3 |  |
| 9930111J21Rik1 |  |
| Dusp1 |  |
| Fkbp15 |  |
| Micall2 |  |
| Gprin3 |  |
| Havcr2 |  |
| Sphk2 |  |
| Mef2a |  |
| Lipa |  |
| Psen2 |  |
| Macir |  |
| Cdk6 |  |
| Zdhhc14 |  |
| Sncg |  |
| Gng2 |  |
| M6pr |  |
| LOC115486430 |  |
| Pla2g15 |  |
| Mafb |  |
| Emilin2 |  |
| Glul |  |
| Abhd12 |  |
| Serpinb6b |  |
| Ankrd66 |  |
| Irf7 |  |
| Psd2 |  |
| Cfap53 |  |
| Usp25 |  |
| Trim30a |  |
| Ctsz |  |
| Fbxo33 |  |
| Ifi27l2b |  |
| Cyp4v3 |  |
| Prss46 |  |
| Stra6l |  |
| 3425401B19Rik |  |
| Atf7ip2 |  |
| Slfn3 |  |
| Pnck |  |
| Ssh2 |  |
| Spsb1 |  |
| Pced1b |  |
| Grid1 |  |
| Gm29825 |  |
| Kctd12b |  |
| Gbp2 |  |
| Galr2 |  |
| Mthfs |  |
| Il15 |  |
| Ccdc125 |  |
| Pram1 |  |
| Serinc3 |  |
| Scamp5 |  |
| Stard3 |  |
| Ccr2 |  |
| Ago4 |  |
| Tnfsf10 |  |
| Pcdhga5 |  |
| Slc9a3r1 |  |
| Creg2 |  |
| Oas2 |  |
| Ephx2 |  |
| Msx2 |  |
| Gpnmb |  |
| Slc26a7 |  |
| Mertk |  |
| Lgals3bp |  |
| Cercam |  |
| Dgkz |  |
| Dnase2a |  |
| Padi4 |  |
| St6galnac4 |  |
| Tpd52 |  |
| Isl2 |  |
| Ifi207 |  |
| Asb4 |  |
| Coro7 |  |
| Gab3 |  |
| Lonrf3 |  |
| Ralgapa2 |  |
| Ehd4 |  |
| Ptger4 |  |
| Erbb3 |  |
| Ap1b1 |  |
| Aldoc |  |
| H2-T-ps |  |
| Adrb2 |  |
| Npl |  |
| Gzme |  |
| Fam214b |  |
| Por |  |
| Kcnmb1 |  |
| Tnip1 |  |
| Gch1 |  |
| Nfkbie |  |
| Tcirg1 |  |
| Nog |  |
| Treml4 |  |
| 4933430I17Rik |  |
| H2-Ob |  |
| Cracr2a |  |
| Xdh |  |
| Syngr1 |  |
| Nxt2 |  |
| Atp6v0a1 |  |
| Isg15 |  |
| Pik3cd |  |
| Serpinb1c |  |
| Irgm1 |  |
| Tap2 |  |
| Zswim5 |  |
| Oasl2 |  |
| Il27 |  |
| Slit1 |  |
| Tmod1 |  |
| Osbpl8 |  |
| Radx |  |
| Rgs20 |  |
| Plin2 |  |
| Clec4b1 |  |
| Ly6k |  |
| Gbx2 |  |
| LOC101055672 |  |
| Cyth1 |  |
| Adamdec1 |  |
| Cpd |  |
| Zbtb8b |  |
| 2310030G06Rik |  |
| Tap1 |  |
| Colq |  |
| Ppt2 |  |
| Cryl1 |  |
| Gm46272 |  |
| Skap2 |  |
| Slc16a13 |  |
| Fgr |  |
| Slfn8 |  |
| Gstt3 |  |
| Nrcam |  |
| Adam15 |  |
| Ubl3 |  |
| Gbp4 |  |
| Slc6a6 |  |
| Eef1a2 |  |
| Smim24 |  |
| Cenpv |  |
| Gmip |  |
| Gm11545 |  |
| Htatip2 |  |
| Shpk |  |
| Selenop |  |
| Mlph |  |
| Pcdhb3 |  |
| Gpr183 |  |
| Ldlrap1 |  |
| Rbpms |  |
| Cdiptos |  |
| Wfdc1 |  |
| Pth1r |  |
| Dmrt2 |  |
| Pdxk |  |
| Rgs11 |  |
| Map3k1 |  |
| Olfr99 |  |
| Abcg3 |  |
| Syk |  |
| Fgl2 |  |
| Map3k11 |  |
| Gclm |  |
| Tnni2 |  |
| Phf11a |  |
| Akr1c13 |  |
| Calcb |  |
| Gpr146 |  |
| Ambp |  |
| Ifnar2 |  |
| Evi2b |  |
| Acss1 |  |
| Jun |  |
| Tmem114 |  |
| Pnpla2 |  |
| Sh3bp5 |  |
| Spef2 |  |
| Nlrc5 |  |
| Nkx3-2 |  |
| Fgd4 |  |
| Pde6h |  |
| C130050O18Rik |  |
| Ifih1 |  |
| Bmx |  |
| Mgst1 |  |
| Ifi208 |  |
| Tmem150b |  |
| Hexa |  |
| Pkd1l2 |  |
| Tspan14 |  |
| Plbd1 |  |
| Hvcn1 |  |
| Gpr27 |  |
| Tnfaip2 |  |
| C2 |  |
| Rerg |  |
| Ptpn7 |  |
| Paqr7 |  |
| LOC546061 |  |
| Nat8l |  |
| Plscr4 |  |
| Slc16a10 |  |
| Trim34a |  |
| Lmod1 |  |
| Matn1 |  |
| Ero1b |  |
| Zfp534 |  |
| Gltp |  |
| Kcnj2 |  |
| Trim30b |  |
| Slc27a1 |  |
| Gpr132 |  |
| Aph1c |  |
| Fli1 |  |
| Ly75 |  |
| Ppfia3 |  |
| Serpina3f |  |
| Rgs10 |  |
| Zfp982 |  |
| Atp6v1b2 |  |
| Spaar |  |
| Sucnr1 |  |
| Fam167a |  |
| Sdr42e1 |  |
| Shisa2 |  |
| Capg |  |
| Pou3f3 |  |
| Cdc42se2 |  |
| Cdk18 |  |
| Ly6e |  |
| Arhgdib |  |
| LOC100039029 |  |
| Irak2 |  |
| Phldb3 |  |
| Ldb3 |  |
| Ube2l6 |  |
| Pla1a |  |
| Pde2a |  |
| Lrp12 |  |
| Fuca2 |  |
| Strip2 |  |
| Tecpr1 |  |
| Kcnu1 |  |
| Nrp2 |  |
| Fth1 |  |
| Snx10 |  |
| P2ry1 |  |
| Mitf |  |
| Slc16a6 |  |
| Dapk1 |  |
| Cmtm6 |  |
| Plekhm3 |  |
| Gpr35 |  |
| Dram1 |  |
| Glrx |  |
| Ceacam16 |  |
| Clec1b |  |
| Gm2619 |  |
| Pcp4l1 |  |
| Acox3 |  |
| Grn |  |
| Retn |  |
| Rgs9 |  |
| Ceacam1 |  |
| Ramp1 |  |
| Nlk |  |
| Dnajc12 |  |
| Fam20c |  |
| Tmem268 |  |
| Cd83 |  |
| Sytl2 |  |
| Tbx2 |  |
| Mctp1 |  |
| Znfx1 |  |
| Fgd3 |  |
| Slc43a3 |  |
| Cd300ld4 |  |
| LOC115489410 |  |
| Bcl6b |  |
| Iigp1 |  |
| Zfp981 |  |
| Errfi1 |  |
| Gm52795 |  |
| Pcdha4 |  |
| Shfl |  |
| Arhgef6 |  |
| LOC100861978 |  |
| Grhl1 |  |
| Cd79b |  |
| LOC115489311 |  |
| Zfp811 |  |
| Slc7a4 |  |
| Sephs2 |  |
| Sez6l2 |  |
| Elmo1 |  |
| Ccn5 |  |
| Rbks |  |
| Pcdhb2 |  |
| Wdr49 |  |
| Cygb |  |
| Trex1 |  |
| Cerkl |  |
| Htr2b |  |
| Aldh1b1 |  |
| Pacc1 |  |
| Exoc6 |  |
| Creb5 |  |
| Prkn |  |
| H2-T24 |  |
| Ifngr1 |  |
| Pitpnc1 |  |
| LOC108167387 |  |
| Tspan13 |  |
| Dusp3 |  |
| Dpep1 |  |
| Cyp4f16 |  |
| Lyzl4 |  |
| Nrap |  |
| Atf3 |  |
| Slamf8 |  |
| Sdk2 |  |
| Nipal3 |  |
| Treml1 |  |
| Tnfsf15 |  |
| Gm46894 |  |
| Slc31a1 |  |
| Vamp5 |  |
| Pax2 |  |
| Gla |  |
| Gdpd5 |  |
| B3gnt7 |  |
| Syt3 |  |
| Herc6 |  |
| Qpct |  |
| Fsd2 |  |
| Gm13212 |  |
| Atp6v0d2 |  |
| H2-T23 |  |
| Lbp |  |
| Krt87 |  |
| Slc24a5 |  |
| Rbm47 |  |
| Spic |  |
| Chd7 |  |
| Gsap |  |
| Zdhhc18 |  |
| Slc36a2 |  |
| Spata48 |  |
| Cd101 |  |
| Rsc1a1 |  |
| BC035044 |  |
| Fam124a |  |
| Zranb3 |  |
| Erap1 |  |
| Prl2c5 |  |
| Epop |  |
| Gbp9 |  |
| Gsr |  |
| Fabp4 |  |
| Pip4k2a |  |
| Sod3 |  |
| Tent5a |  |
| Nos2 |  |
| Idua |  |
| Ccdc65 |  |
| Slc22a18 |  |
| Nacad |  |
| Spp1 |  |
| Hey1 |  |
| Sult2b1 |  |
| Ccl24 |  |
| Trim12a |  |
| Scgb3a1 |  |
| Mapk13 |  |
| Slc8b1 |  |
| Cyrib |  |
| Tmem176b |  |
| Mndal |  |
| Slc6a19 |  |
| Otor |  |
| Epb41l4b |  |
| Gpr31b |  |
| Tmc6 |  |
| Fhad1 |  |
| Epb41 |  |
| Gm42517 |  |
| Mansc1 |  |
| Trim65 |  |
| 1700001C19Rik |  |
| Draxin |  |
| Cyria |  |
| Mylpf |  |
| Cd302 |  |
| Enpp6 |  |
| Fam241a |  |
| B3gnt3 |  |
| Ccdc184 |  |
| Ap5b1 |  |
| Dpysl4 |  |
| Hfe |  |
| Adcy7 |  |
| Rnase2b |  |
| Irf1 |  |
| Acsl4 |  |
| Tlr3 |  |
| Irgm2 |  |
| Sec11c |  |
| Coa4 |  |
| Fcrl5 |  |
| Pdlim4 |  |
| Tppp |  |
| Ptpn22 |  |
| Acsl1 |  |
| Naip1 |  |
| Sod2 |  |
| Cmpk2 |  |
| Gimap9 |  |
| Tubb3 |  |
| Derl3 |  |
| Daglb |  |
| Rnf144b |  |
| Aldh1a2 |  |
| Chn2 |  |
| Gm36079 |  |
| Gm42368 |  |
| Tifa |  |
| Wnt4 |  |
| Gstt2 |  |
| Rasgrp3 |  |
| Gm33373 |  |
| Arhgap15 |  |
| Slc39a8 |  |
| Colec10 |  |
| Emb |  |
| Trem3 |  |
| Hmgcll1 |  |
| Shisal1 |  |
| Ccdc69 |  |
| Il18 |  |
| Tns4 |  |
| Sectm1a |  |
| Tspan18 |  |
| Neu1 |  |
| Snx8 |  |
| Gbp10 |  |
| Nfkbia |  |
| Lratd2 |  |
| Skint3 |  |
| Nfkbiz |  |
| Prkx |  |
| Rnf207 |  |
| Klhdc7a |  |
| Stat4 |  |
| Hrh2 |  |
| Akap3 |  |
| Stat1 |  |
| Hpse |  |
| Hap1 |  |
| St14 |  |
| Tg |  |
| Alpk1 |  |
| Mak |  |
| Gldn |  |
| Lims2 |  |
| Tmem151b |  |
| Lrrc32 |  |
| Olfr920 |  |
| Dkk3 |  |
| Adgrl4 |  |
| Selenbp1 |  |
| Tnfrsf21 |  |
| Aqp9 |  |
| Lgr6 |  |
| Card11 |  |
| Mdk |  |
| Rnasel |  |
| Cdcp3 |  |
