# Supplementary material for: Melatonin enhances osteoblastogenesis of senescent bone marrow stromal cells through NSD2‐mediated chromatin remodelling
Source: Clin Transl Med. 2022 Feb 27;12(2):e746. doi: 10.1002/ctm2.746 (PMC8882236; doi:10.1002/ctm2.746)
Supplement: Supplementary file 3 — Table S3 [file CTM2-12-e746-s005.docx]

**Table S 3. Overlapped genes of RNA-seq and ChIP-seq in aged mouse BMSCs treated with melatonin.**

| **GeneSymbol** |
| --- |
| Pard3b |
| Arhgap15 |
| Slc15a3 |
| Il33 |
| Macrod2 |
| Inpp4b |
| Twist1 |
| Tnfaip8 |
| Cd28 |
| Elmo1 |
| Cd180 |
| Arhgap6 |
| Ppm1l |
| Abcg2 |
| Pi15 |
| Mboat1 |
| Il1a |
| Mrc1 |
| Slc24a3 |
| Lvrn |
| Slc39a12 |
| Sema6d |
| Pparg |
| Qpct |
| Malrd1 |
| Adgrl4 |
| Sema3a |
| Atf4 |
| Slit2 |
| Rnf17 |
| Ccdc28b |
| A830018L16Rik |
| Tnip3 |
| Arhgap20 |
| Plcl2 |
| Tnfsf13b |
| Ppm1h |
| Mctp1 |
| Tbxas1 |
| Arhgap32 |
| Dab2 |
| Zfpm2 |
| Vav3 |
| Emcn |
| Adgrl3 |
| Chn2 |
| Pkhd1l1 |
| Epha7 |
| Plekha5 |
| Ugt1a7c |
| Ptpn22 |
| Map2 |
| Serpinb6b |
| Gcnt4 |
| Rasgrp3 |
| Mmp3 |
| Cdrt4 |
| Ly86 |
| Ssbp2 |
| Fpr2 |
| Mmp13 |
| Ccr2 |
| Kcnj16 |
| Edil3 |
| Scel |
| Map3k7cl |
| Arl5b |
| Osbpl6 |
| Postn |
| Por |
| Ccr3 |
| Spats2l |
| Cdk1a |
| Atrnl1 |
| Mertk |
| Mitf |
| Zdhhc14 |
| Pla2g7 |
| Mmp8 |
| Cxcl1 |
| Map3k5 |
| Tmem26 |
