# Supplementary material for: Melatonin enhances osteoblastogenesis of senescent bone marrow stromal cells through NSD2‐mediated chromatin remodelling
Source: Clin Transl Med. 2022 Feb 27;12(2):e746. doi: 10.1002/ctm2.746 (PMC8882236; doi:10.1002/ctm2.746)
Supplement: Supplementary file 4 — Supporting Information [file CTM2-12-e746-s001.docx]

**Key resources of this study**

| **Category** | **Source** | **Cat. No.** |
| --- | --- | --- |
| **Antibodies** | | |
| Anti-Histone H3 (di methyl K36) antibody-ChIP Grade | Abcam | ab9049 |
| Anti-Histone H3 (di methyl K36) antibody | Abcam | ab272158 |
| Anti-Histone H3 (tri methyl K27) antibody-ChIP Grade | Abcam | ab6002 |
| Anti-WHSC1/NSD2 antibody [29D1] - ChIP Grade | Abcam | ab75359 |
| Recombinant Anti-WHSC1/NSD2 antibody | Abcam | ab259940 |
| Anti-RUNX2 (C-12) | Santa Cruz Biotechnology | sc-390715 |
| Anti-rabbit β-actin | Abclonal | AC006 |
| Goat Anti-Rabbit IgG-HRP | Sigma-Aldrich | A0545 |
| Rabbit Anti Mouse IgG-HRP | Sigma-Aldrich | A9044-2ML |
| Anti-Histone H3 antibody - Nuclear Loading Control and ChIP Grade | Abcam | ab1791 |
| Leptin Receptor Antibody-C-terminal | Affinity | DF7139 |
| Rabbit IgG control Polyclonal antibody | Proteintech | 30000-0-AP |
| Anti-mouse IgG | Proteintech | B900620 |
| PE Mouse IgG1, κ Isotype Ctrl | BioLegend | 981804 |
| PE Mouse IgG2a, κ Isotype Ctrl | BioLegend | 981910 |
| PE anti-human CD105 Antibody | BioLegend | 323205 |
| PE anti-human CD45 Antibody | BioLegend | 304007 |
| PE anti-human CD14 | BioLegend | 982508 |
| PE anti-human CD90 (Thy1) Antibody | BioLegend | 328109 |
| PE anti-mouse CD105 Antibody | BioLegend | 120407 |
| PE Rat IgG2a, κ Isotype Ctrl Antibody | BioLegend | 400507 |
| PE anti-mouse CD90.2 Antibody | BioLegend | 105307 |
| PE anti-mouse CD14 Antibody | BioLegend | 150105 |
| PE anti-mouse CD45 Antibody | BioLegend | 147711 |
| PE Rat IgG2b, κ Isotype Ctrl Antibody | BioLegend | 400607 |
| Anti-pan-AKT antibody | Abcam | ab8805 |
| Anti-AKT (phospho T308) antibody | Abcam | ab38449 |
| Anti-ERK1 + ERK2 antibody [EPR17526] | Abcam | ab184699 |
| Anti-Erk1 (pT202/pY204) + Erk2 (pT185/pY187) antibody [EP197Y] | Abcam | ab76299 |
| Anti-NF-kB p65 antibody [E379] | Abcam | ab32536 |
| Anti-NF-kB p65 (phospho S536) antibody [EP2294Y] | Abcam | ab76302 |
| β-Catenin (D10A8) XP® Rabbit mAb | Cell Signaling Technology | 8480 |
| CREB (48H2) Rabbit mAb | Cell Signaling Technology | 9197 |
| NF-κB p65/RelA (D14E12) XP® Rabbit mAb | Cell Signaling Technology | 8242 |
| LEF1 (D6J2W) Rabbit mAb | Cell Signaling Technology | 76010 |
| **Drugs** | | |
| Melatonin | Sigma-Aldrich | M5250 |
| 2-methyl-2-butanol, tertiary amyl alcohol 99+ % | Sigma-Aldrich | 240486-5ML |
| 2,2,2-tribromoethanol | Sigma-Aldrich | T48402-5GM |
| Penicillin/streptomycin | BIOSOURCE | P303-100 |
| Puromycin 2HCL | SelleckChem | S7417 |
| β-glycerol phosphate | Sigma-Aldrich | 50020 |
| L-ascorbic acid phosphate | Wako | 013-12061 |
| Dexamethasone sodium phosphate | Sigma-Aldrich | D8893 |
| Glutamine | BIOSOURCE | P300-100 |
| Alpha MEM | Gibco | 12571-063 |
| 2-ME | GIBCO | 21985-023 |
| MK-2206 2HCl | SelleckChem | S1078 |
| SCH772984 | SelleckChem | S7101 |
| TPCA-1 | SelleckChem | S2824 |
| Wnt-C59 (C59) | SelleckChem | S7037 |
| **Enzymes** | | |
| RNase A, DNase and protease-free | Thermo Fisher | EN0531 |
| Proteinase K Solution, ChIP grade | Thermo Fisher | 26160 |
| Benzonase Nuclease | Sigma-Aldrich | E1014-25KU |
| FastAP Thermosensitive Alkaline Phosphatase | Thermo Fisher | EF0651 |
| AgeⅠ | NewEngland Biolabs | R0580S |
| NotI-HF | NewEngland Biolabs | R3189S |
| BamHI | NewEngland Biolabs | R0136S |
| XbaI | NewEngland Biolabs | R0145S |
| EcoRI | NewEngland Biolabs | R0101S |
| KpnI-HF | NewEngland Biolabs | R3142S |
| HindIII | NewEngland Biolabs | R104S |
| XhoI | NewEngland Biolabs | R0146S |
| DpnI | NewEngland Biolabs | R0176S |
| 2 × Phanta Max Master Mix (Dye Plus) | Vazyme | P525-01 |
| CutSmart Buffer | NewEngland Biolabs | 137204S |
| NEBuffer1 | NewEngland Biolabs | B7001S |
| NEBuffer2 | NewEngland Biolabs | B7002S |
| NEBuffer3 | NewEngland Biolabs | B7003O |
| NEBuffer4 | NewEngland Biolabs | B7004S |
| T4 DNA Ligase | NewEngland Biolabs | M0202S |
| 10×Buffer for T4 DNA ligase | NewEngland Biolabs | B0202S |
| Multiscribe Reverse Transcriptase | ABI | 4308228 |
| dNTP mix | ABI | 362275 |
| **Plasmids** | | |
| pCMV3-C-HA-NSD2 | Sino Biological | HG11530-CY |
| pCMV3-C-HA Negative Control Vector | Sino Biological | CV013 |
| pLV-C-FLAG-NSD2 | Sino Biological | HG11530-CFLN |
| pLV-C-FLAG Lentivirus Control | Sino Biological | LVCV-04 |
| psPAX_2_ | Addgene | 12260 |
| PMD_2_G | Gift from Dr. Xudong Wu, Tianjin Medical University, Dept. Cell Biology | |
| pLKO.1 vector | Gift from Dr. Feng Wang, Tianjin Medical University, Dept. Genetics | |
| NSD2-shRNA1 | Self-construction | |
| NSD2-shRNA2 | Self-construction | |
| NSD2-shRNA3 | Self-construction | |
| NSD2-shRNA4 | Self-construction | |
| pGL3-basic-hNSD2-promoter-2kb | Self-construction | |
| pGL3-basic-hNSD2-promoter-2kb-ΔBS1 | Self-construction | |
| pGL3-basic-hNSD2-promoter-2kb-ΔBS2 | Self-construction | |
| pGL3-basic-hNSD2-promoter-2kb-ΔBS3 | Self-construction | |
| **Critical Commercial Assays** | | |
| EvaGreen 2× qPCR MasterMix-Low ROX | abm | MasterMix-LR |
| 5×All-In-One RT MasterMix | abm | G490 |
| Pierce BCA Protein Assay Kit | Thermo SCIENTIFIC | 23225 |
| AxyPrep DNA Extraction Kit | AXYGEN | 295 AP-GX-250G |
| AxyPrep Plasmid Miniprep Kit | AXYGEN | 183 AP-MN-P-250G |
| Plasmid Maxi Kit(25) | QIAGEN | 12163 |
| EnVision G12 Doublestain System, Rabbit/Mouse(DAB+/Permanent Red) | Dako | K5361 |
| SuperSignal West Dura Extended Duration Substrate | ThermoFisher | 34580 |
| 9002 SimpleCHIP® Kit | Cell Signaling | 22188S |
| Simple CHIP® Kits-20C-Reagents | Cell Signaling | 45061S |
| ChIP-grade Protein A/G Magnetic Beads | Thermo SCIENTIFIC | 26162 |
| Lipofectamine 3000 Transfection Kit | Invitrogen | L3000-008 |
| Polybrene Infection / Transfection Reagent | Sigma-Aldrich | TR-1003 |
| CellTiter 96 Aqueous One Solution | Promega | G358B |
| NuPAGE 4-12% Bis-Tris Gel | Invitrogen | NP0335BOX |
| Poly (ethylene glycol) 8,000 | Sigma-Aldrich | TR-1003 |
| Senescence β-Galactosidase Staining Kit | Beyotime | C0602 |
| Alkaline Phosphatase Activity Colorimetric Assay Kit | BioVision | K412 |
| BCIP / NBT basic phosphatase reagent kit | Beyotime | C3206 |
| Alizarin red | Sigma-Aldrich | A5533 |
| 10% neutral formalin buffer | Sigma-Aldrich | HT501128 |
| Cetyl pyridinium chloride | Sigma-Aldrich | 1104006 |
| Na-phosphate buffer | Sigma-Aldrich | 71640 |
| 37% formaldehyde | Sigma-Aldrich | 252549 |
| p-nitrophenyl phosphate solution | Sigma-Aldrich | 487663 |
| p-NPP Substrate Buffer | Sigma-Aldrich | 487664 |
| Human MT (Melatonin) ELISA Kit | Elabscience | E-EL-H2016c |
| Mouse MT (Melatonin) ELISA Kit | Elabscience | E-EL-M0788c |
| Ficoll-Paque PLUS endotoxin tested | GE Healthcare | 17-1440-02 |
| TRIzol Reagent | Ambion, Life Science | 15596018 |
| Opti-MEM®I(1×) Reduced Serum | Gibco, Life Technologies | 31985-070 |
| Opti-protein XL Marker | abm | G266 |
| PageRuler Prestained protein Ladder | ThermoFisher Scientific | 26616 |
| 1Kb Ladder DNA Marker | Biomed | MD114 |
| 1Kb DNA Ladder | TIANCEN | MD111 |
| 100bp DNA Ladder | TRANS | BM301 |
| BM15000 DNA Marker | Biomed | MD106 |
| 1Kb Plus DNA Ladder | Solarbio | M1500 |
| Polyethylenimine, Linear, MW 25000, Transfection Grade (PEI 25K™) | Polysciences | 23966-1 |
| PEI-Transferrinfection Kit | ThermoFisher Scientific | BMS1003 |
| Toluidine blue O (C.I.52040) | Sigma-Aldrich | 92-31-9 |
| Hematoxylin and Eosin Staining Kit | Beyotime | C0105S |
| Universal Genomic DNA Extraction Kit | Solarbio | D2100 |
| QuickMutation Plus Gene Site Directed Mutation Kit | Beyotime | D0208S |
| The Immunodiagnostic Systems Limited Rat/Mouse P1NP EIA kit | Immunodiagnostic Systems | AC-33F1 |
| **Primers sequence** | | |
| humanGAPDH-F | TTGCCCTCAACGACCACTTT | |
| humanGAPDH-R | TGGTCCAGGGGTCTTACTCC | |
| humanNSD2-F | CCCATACGAAAGTGCAGACG | |
| humanNSD2-R | CACAGCTGGCACACATACTC | |
| humanRUNX2-F | GGACGAGGCAAGAGTTTCAC | |
| humanRUNX2-R | GAGGCGGTCAGAGAACAAAC | |
| humanCOL1A1-F | CAAAGGTGCTGATGGCTCTC | |
| humanCOL1A1-R | CCACTTTCACCCTTGTCACC | |
| humanOPN-F | ACACATATGATGGCCGAGGT | |
| humanOPN-R | CTCGCTTTCCATGTGTGAGG | |
| humanBGLAP-F | ACCGAGACACCATGAGAGCC | |
| humanBGLAP-R | CCATTGATACAGGTAGCGCCT | |
| humanMT1-F | TCAGGAACGCAGGAAACATC | |
| humanMT1-R | CCATCAGGAACCCACTGACT | |
| humanMT2-F | CCTTGCTGCCCAACTTCTTT | |
| humanMT2-R | CAGCGATAGGGAGGAGGAAG | |
| hNSD2sh#1-F | CCGGCGGAAAGCCAAGTTCACCTTTCTCGAGAAAGGTGAACTTGGCTTTCCGTTTTTG | |
| hNSD2sh#1-R | AATTCAAAAACGGAAAGCCAAGTTCACCTTTCTCGAGAAAGGTGAACTTGGCTTTCCG | |
| hNSD2sh#2-F | CCGGATCTTACTTCCCGGGTGTTTACTCGAGTAAACACCCGGGAAGTAAGATTTTTTG | |
| hNSD2sh#2-R | AATTCAAAAAATCTTACTTCCCGGGTGTTTACTCGAGTAAACACCCGGGAAGTAAGAT | |
| hNSD2sh#3-F | CCGGCCCAGAAAGAGCTTGGATATTCTCGAGAATATCCAAGCTCTTTCTGGGTTTTTG | |
| hNSD2sh#3-R | AATTCAAAAACCCAGAAAGAGCTTGGATATTCTCGAGAATATCCAAGCTCTTTCTGGG | |
| hNSD2sh#4-F | CCGGCCCAGAAAGAGCTTGGATATTCTCGAGAATATCCAAGCTCTTTCTGGGTTTTT | |
| hNSD2sh#4-R | AATTCAAAAACCCAGAAAGAGCTTGGATATTCTCGAGAATATCCAAGCTCTTTCTGGG | |
| mouseGAPDH-F | TCAAGCTCATTTCCTGGTATGACA | |
| mouseGAPDH-R | TAGGGCCTCTCTTGCTCAGT | |
| mouseNSD2-F | GCACAGTCTTCGGAAGCAGA | |
| mouseNSD2-R | AGCCCGATTTCGCTTCTTCA | |
| mouseRUNX2-F | GCGGTGCAAACTTTCTCCAG | |
| mouseRUNX2-R | ACTGCTTGCAGCCTTAAATATTCC | |
| mouseCOL1A1-F | CGATGGATTCCCGTTCGAGT | |
| mouseCOL1A1-R | CGATCTCGTTGGATCCCTGG | |
| mouseOPN-F | CTGGCTGAATTCTGAGGGACT | |
| mouseOPN-R | TTCTGTGGCGCAAGGAGATT | |
| mouseBGLAP-F | TTCTGCTCACTCTGCTGACC | |
| mouseBGLAP-R | GGGACTGAGGCTCCAAGGTA | |
| mouseRUNX2-A-F | ATATGGAACTAAGTTCAGAA | |
| mouseRUNX2-A-R | GGCCTTACCACAAGCCTTTT | |
| mouseRUNX2-B-F | ATCATATAATTATTATGTTG | |
| mouseRUNX2-B-R | ATGCAGCAGTATATGAAACT | |
| mouseBGLAP-A-F | CCGAACAAGCAAGAGGCATC | |
| mouseBGLAP-A-R | GCACCCTCCAGCGTCCAGTA | |
| mouseBGLAP-B-F | GTGATCCTCTGAGGCCAGAA | |
| mouseBGLAP-B-R | AGTGTCACCAGTTAATTGCT | |
| ChIP-qPCR-Runx2-F | CTGTTGCTCAGAACGCCACA | |
| ChIP-qPCR-Runx2-R | AACACAAATGCTGAAGGAGC | |
| ChIP-qPCR-Bglap-F | GCAAATGAGGACATTACTGA | |
| ChIP-qPCR-Bglap-R | GGAGCACTGGGTGGCCCTAG | |
| hNSD2-promoter-2kb-F-KpnI | GGGGTACCATGGCTAGAGTGCAGTGGCATGA | |
| hNSD2-promoter-2kb-R-HindIII | CCCAAGCTTTTAGCAGAAGCCGCGGGAAAAGT | |
| ChIP-PCR-C-F | CTTCCAAAGTGCTTGTATTA | |
| ChIP-PCR-C-R | GCCAAAGTGAAAGGATTGCT | |
| ChIP-PCR-B-F | CTCGCCAGGCTCCCCTGGGC | |
| ChIP-PCR-B-R | GCCGGTGTGATTTGCTGCCC | |
| ChIP-PCR-A-F | GCTTGGGGACCCGCGCGGGC | |
| ChIP-PCR-A-R | TCAGAGTAGCGCCGGGCGCG | |
| hΔBS1-F1-KpnI | GGGGTACCATGGCTAGAGTGCAGTGGCATGA | |
| hΔBS1-R1 | CCGCGCTCAGATCCGCGCCC | |
| hΔBS1-F2 | GGGCGCGGATCTGAGCGCGG | |
| hΔBS1-R2 | CCCAAGCTTTTAGCAGAAGCCGCGGGAAAAGT | |
| hΔBS1-F3 | GGGGTACCATGGCTAGAGTGCAGTGGCATGA | |
| hΔBS1-R3-HindIII | CCCAAGCTTTTAGCAGAAGCCGCGGGAAAAGT | |
| hΔBS2-F1-KpnI | GGGGTACCATGGCTAGAGTGCAGTGGCATGA | |
| hΔBS2-R1 | TTCAGGGAGCTCGGCCTTGA | |
| hΔBS2-F2 | TCAAGGCCGAGCTCCCTGAA | |
| hΔBS2-R2 | CCCAAGCTTTTAGCAGAAGCCGCGGGAAAAGT | |
| hΔBS2-F3 | GGGGTACCATGGCTAGAGTGCAGTGGCATGA | |
| hΔBS2-R3-HindIII | CCCAAGCTTTTAGCAGAAGCCGCGGGAAAAGT | |
| hΔBS3-F1-KpnI | GGGGTACCATGGCTAGAGTGCAGTGGCATGA | |
| hΔBS3-R1 | ACTGTGGGAGGCCGAGACAG | |
| hΔBS3-F2 | CTGTCTCGGCCTCCCACAGT | |
| hΔBS3-R2 | CCCAAGCTTTTAGCAGAAGCCGCGGGAAAAGT | |
| hΔBS3-F3 | GGGGTACCATGGCTAGAGTGCAGTGGCATGA | |
| hΔBS3-R3-HindIII | CCCAAGCTTTTAGCAGAAGCCGCGGGAAAAGT | |
