# Supplementary material for: Melatonin enhances osteoblastogenesis of senescent bone marrow stromal cells through NSD2‐mediated chromatin remodelling
Source: Clin Transl Med. 2022 Feb 27;12(2):e746. doi: 10.1002/ctm2.746 (PMC8882236; doi:10.1002/ctm2.746)
Supplement: Supplementary file 5 — Figure S1‐S6 [file CTM2-12-e746-s002.docx]

**Supplementary figures**


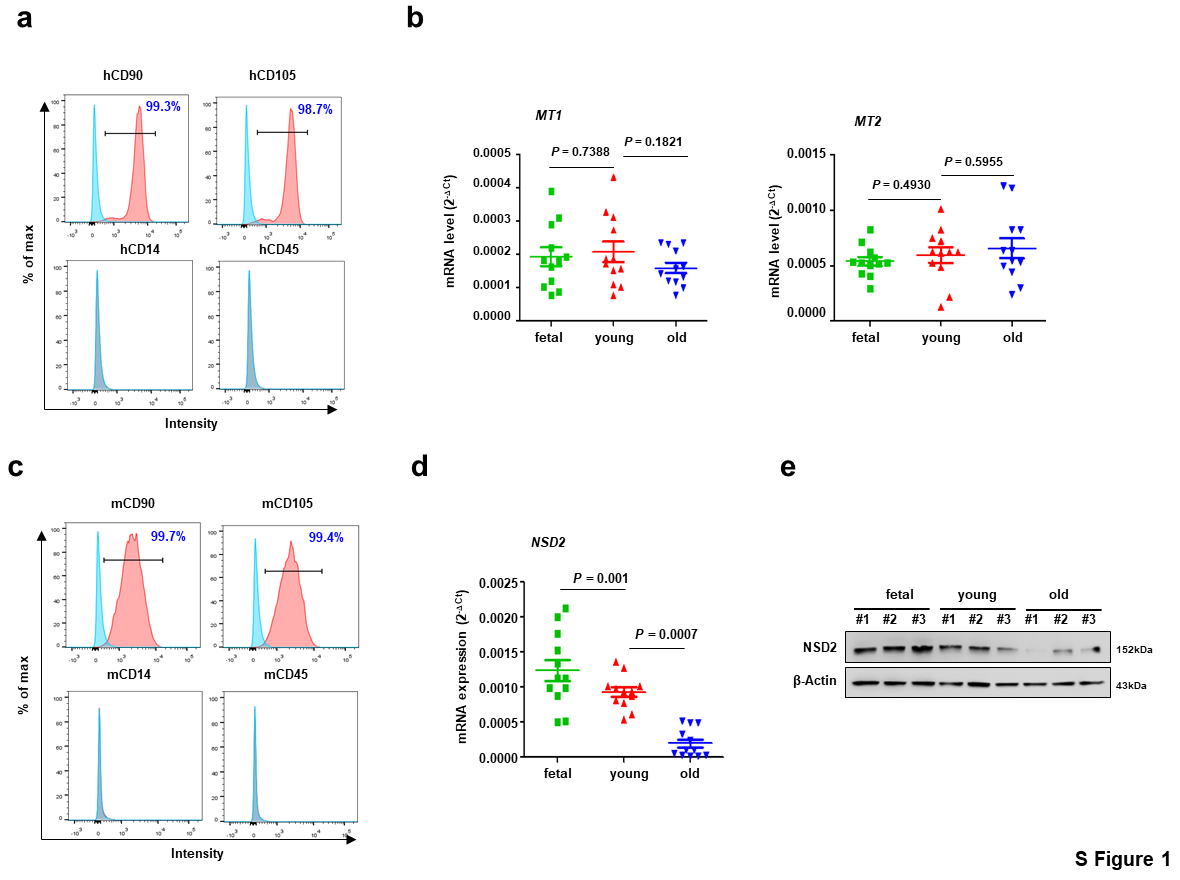


**Figure S 1. Expressions of mesenchymal stem cell markers and melatonin receptors in human and mouse MSCs. a,** Representative flow cytometry analysis of the expressions of positive (CD90, CD105) and negative (CD14, CD45) surface markers of MSCs isolated from human bone marrow. **b,** qPCR analysis of the expression of melatonin receptors in human MSCs. n = 12 human MSCs; n = 3 test per sample. **c**, Representative flow cytometry analysis of the expressions of positive (CD90, CD105) and negative (CD14, CD45) surface markers of MSCs isolated from mouse bone marrow. **d**, qPCR analysis of *NSD2* expression in BMSCs derived from the fetal, young and old donors (n = 12 samples per group). **e**, Representative Western blotting analysis of NSD2 protein in BMSCs from the fetal, young and old donors. Data are mean ± s.e.m. *P* values are determined by unpaired two-sided *t*-tests with Welch’s correction.


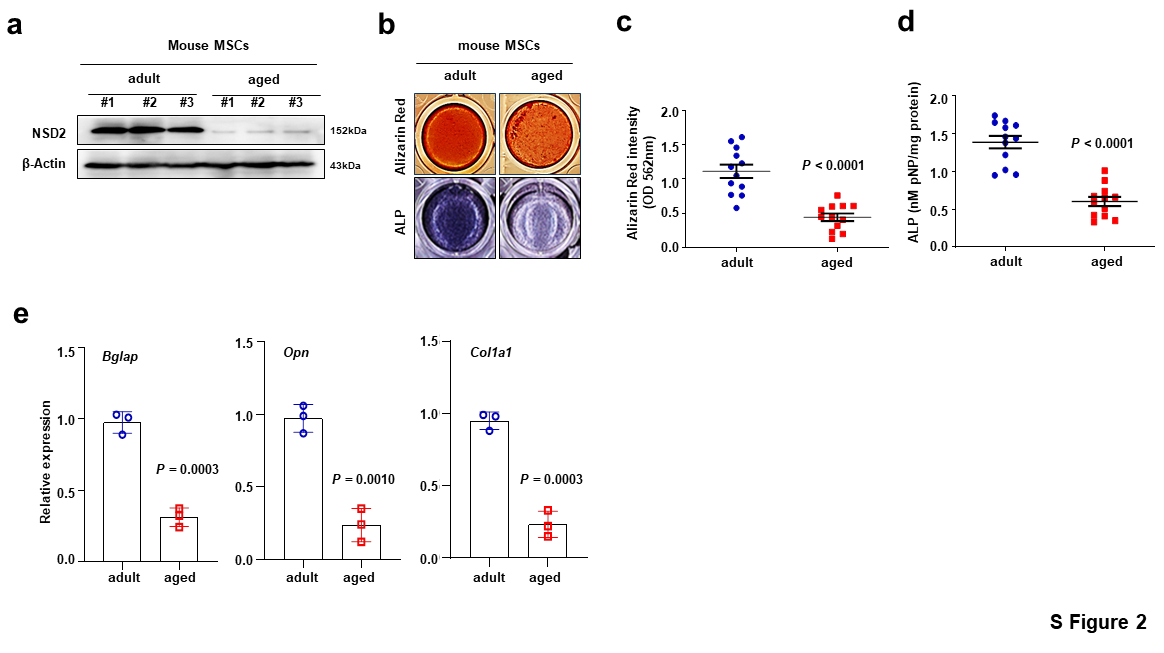


**Figure S 2.** **Osteogenesis of MSCs from adult and aged mouse.** **a,** Western blotting analysis of NSD2 expression in BMSCs from adult and aged mice. **b**, Representative images of Alizarin Red and alkaline phosphatase (ALP) staining for osteogenic differentiation from adult and aged BMSCs. Quantification of (**c**) Alizarin Red S staining and (**d**) ALP activity in each group (n = 12 samples per group). **e**, qPCR analysis of osteogenic marker genes expressions in MSCs from different groups cultured with osteogenic media for 14 days (n = 3 mice per group). Data are mean ± s.e.m. *P* values are determined by unpaired two-sided *t*-tests with Welch’s correction.


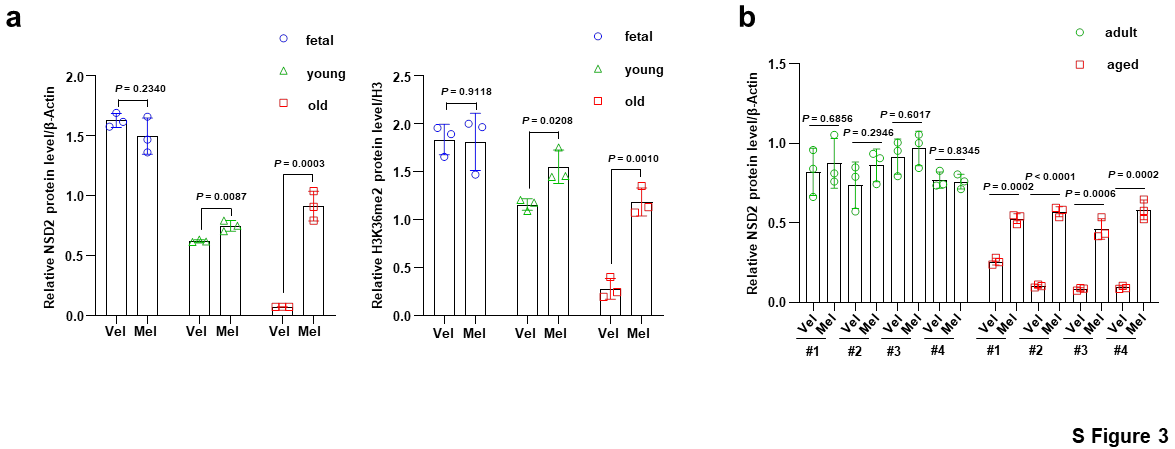


**Figure S 3.** **Melatonin stimulates NSD2 and H3K36me2 expression in senescent BMSCs.** **a, b,** Quantification of fig 4b, 4d Western blotting analysis (n = 3 samples per group). Data are mean ± s.e.m. *P* values are determined by paired two-sided *t*-tests with Welch’s correction.


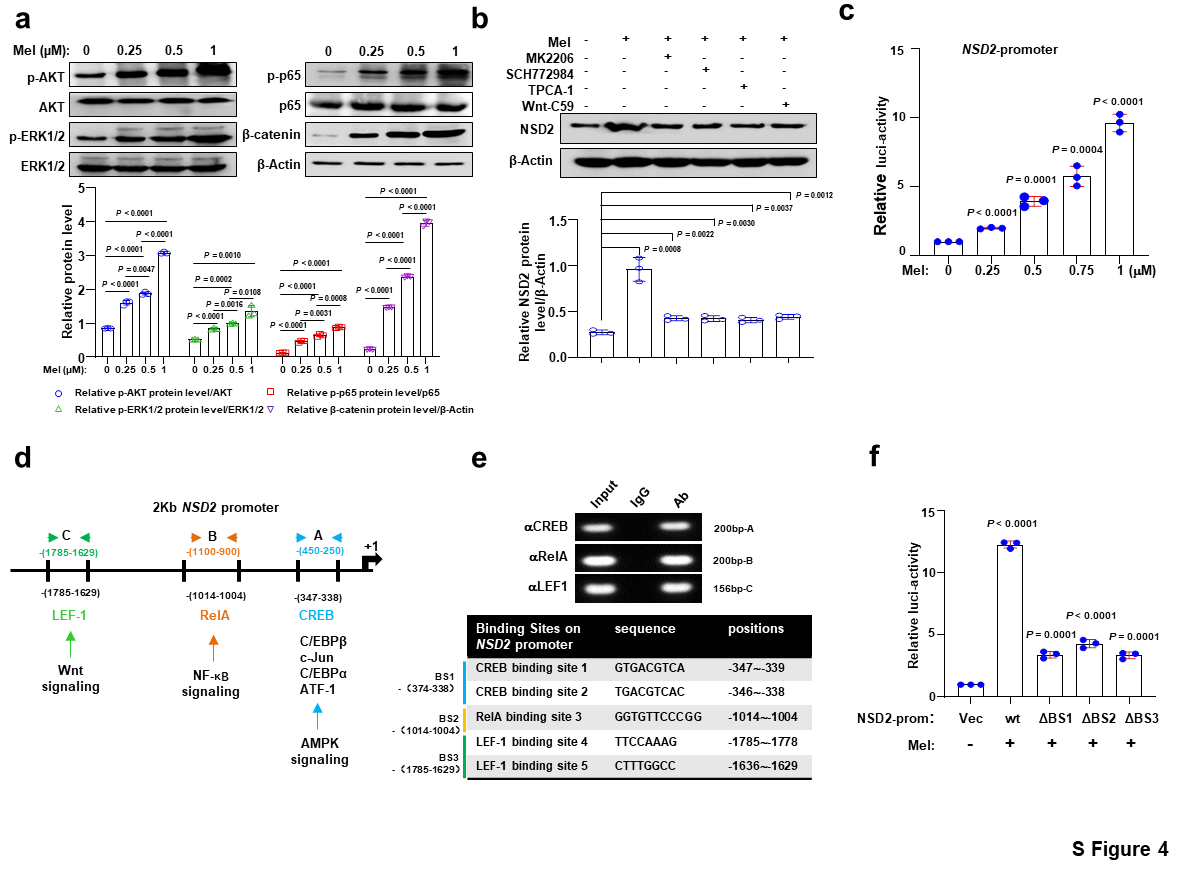


**Figure S 4. NSD2 is a direct downstream target gene of melatonin signaling. a,** Western blotting assay of phosphorylation of ERK1/2, AKT, p65, and the accumulation of β-catenin in human BMSCs treated with 1 μM melatonin for 24 hr, and their corresponding quantifications (n = 3 samples per group). **b,** Western blotting assay of NSD2 expression in BMSCs treated with 1 μM melatonin for 24 hr in presence or absence of inhibitors of AKT (MK2206, 1μM), ERK1/2 (SCH772984, 0.5μM), p65 (TPCA-1, 1μM), and TCF4/LEF1 (Wnt-C59, 1μM), respectively, and their corresponding quantifications (n = 3 samples per group). **c,** Luciferase assay of a 2Kb *NSD2-*promoter transfected in human BMSCs and treated with increasing amount of melatonin for 24 hr. **d,** Schematic illustration showing representative transcriptional factors on the *NSD2* promoter and their positions. **e,** Chromatin immunoprecipitation (ChIP)-PCR assay to detect the binding of these TFs on the *NSD2* promoter in human BMSCs. **f**, Luciferase assay of the *NSD2* promoter with CREB, RelA and LEF1 binding sties mutations in human BMSCs and treated with 1 μM melatonin for 24 hr. Data are mean ± s.e.m. *P* values are determined by two-sided *t*-tests with Welch’s correction.


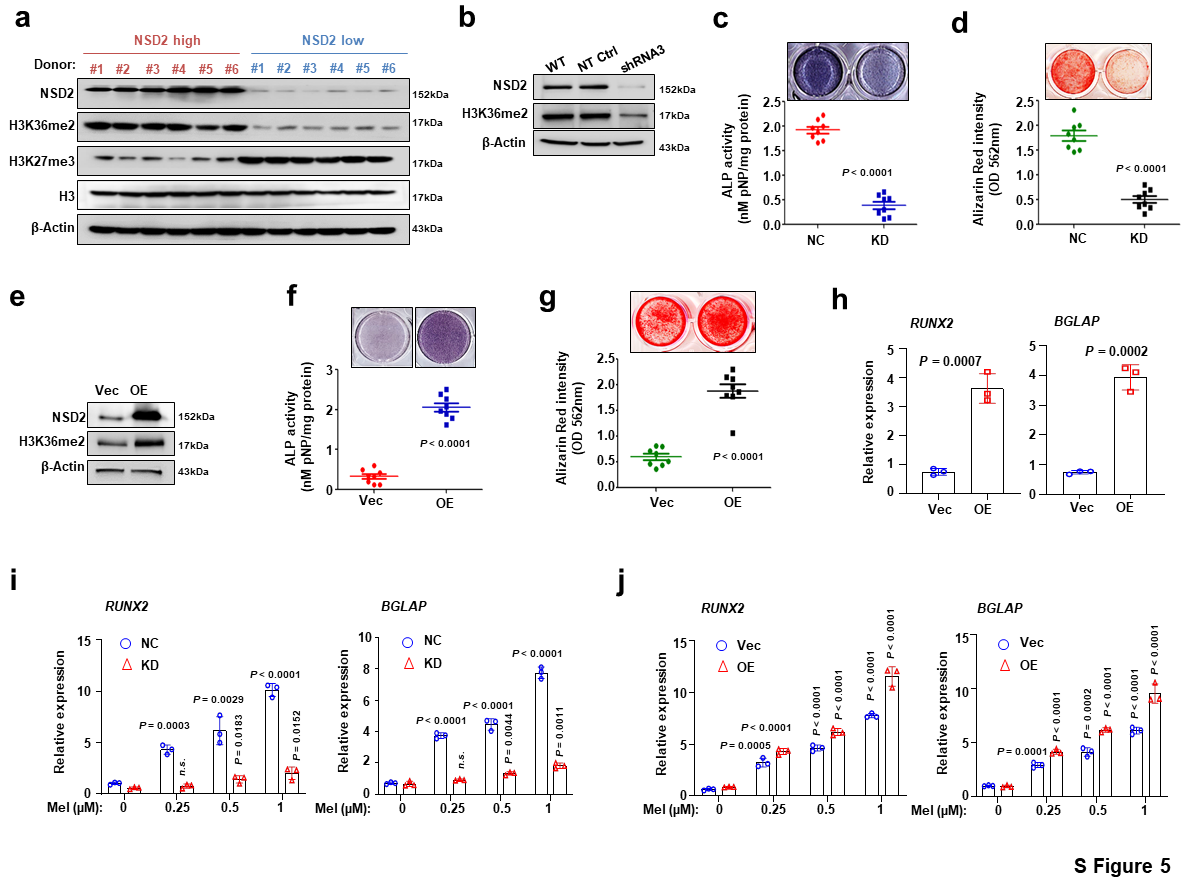


**Figure S 5. Effect of melatonin on osteogenesis of BMSCs depends on NSD2**.

**a**, Western blotting assay of NSD2 expression, the corresponding H3K36me2 and H3K27me3 levels in human BMSCs derived from different donors. **b,** Representative Western blotting analysis of NSD2 knockdown by lentivirus carrying shRNA after 72 hours, and the levels of H3K36me2 in human MSCs. **c,** Representative images of ALP staining and the quantification of ALP activity (n = 8 MSCs per group). **d,** Representative images of Alizarin Red staining and the quantification of Alizarin Red intensity (n = 8 MSCs per group). **e,** Representative Western blotting analysis of lentivirus carrying NSD2 overexpression for 72 hours, and the levels of H3K36me2 in human MSCs. Representative images of (**f**) ALP staining, (**g)** Alizarin Red staining, and their corresponding quantifications (n = 8 MSCs per group). **h,** qPCR assay of expressions of *RUNX2* and *BGALP* in human MSCs with NSD2 overexpression (n = 3 independent experiments). *RUNX2* and *BGLAP* expressions in human MSCs with (**i)** NSD2 silencing and (**j**) NSD2 overexpression, and treated with increasing amount of melatonin for 24 hours (n = 3 independent experiments). Data are mean ± s.e.m. *P* values are determined by paired two-sided *t*-tests with Welch’s correction.


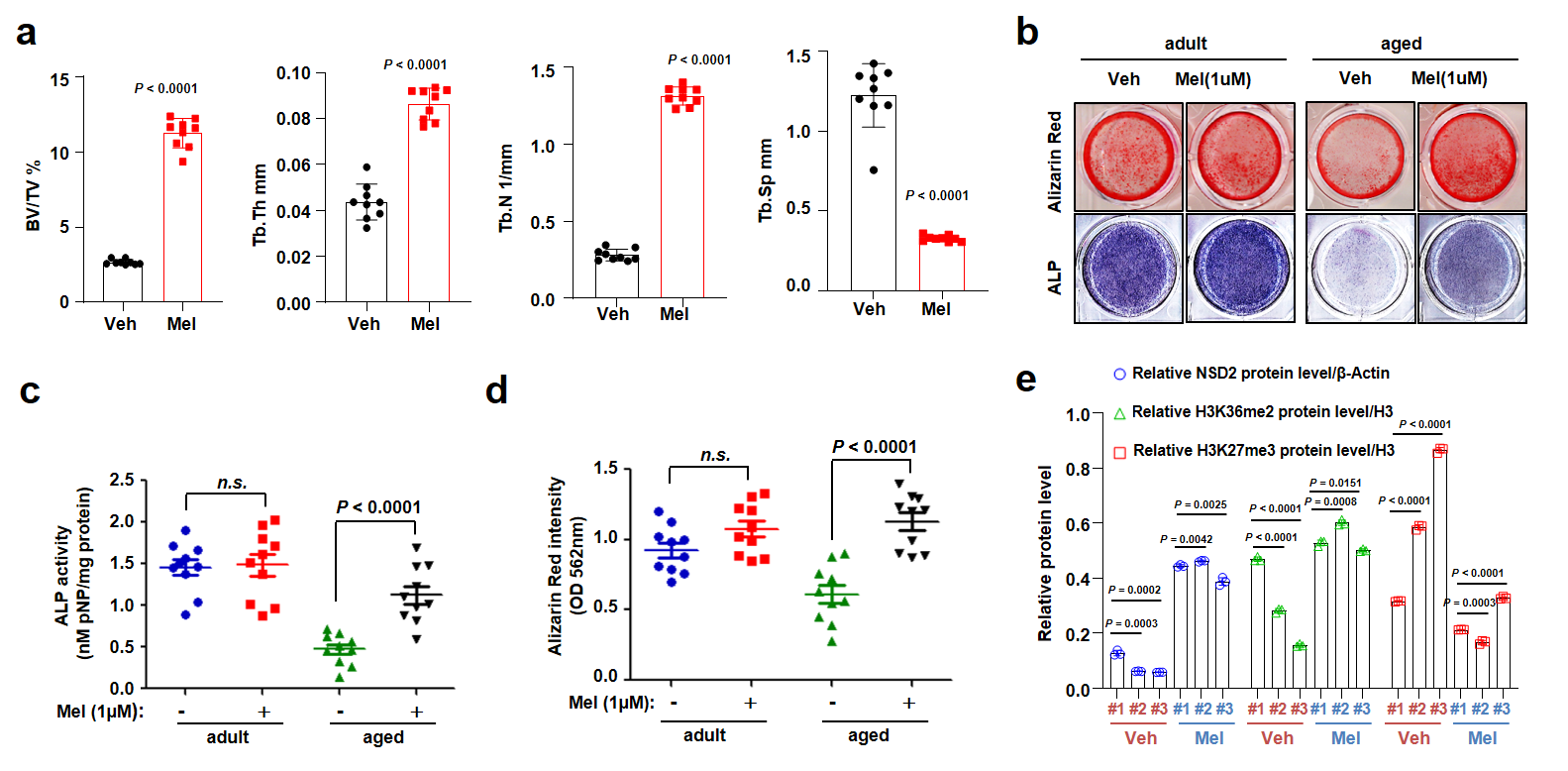


**Figure S 6.** **Melatonin ameliorates osteogenesis of senescent BMSCs *in vivo*.**

**a,** Quantification of microCT analysis of bone volume ratio to tissue volume (BV/TV), trabecular thickness (Tb.Th), and trabecular number (Tb.N) and trabecular separation (Tb.Sp) in aged mice treated with vehicle control or melatonin (n = 9). **b**, Representative images of ALP and Alizarin Red S staining of BMSCs from adult and aged cultured with osteogenic media in presence with vehicle or melatonin for 14 days. Quantification of (**c**) ALP activity and (**d**) Alizarin Red S staining of BMSCs from adult and aged mice treated with vehicle or melatonin (n = 10). **e**, Quantification of fig 7h Western blotting analysis (n = 3 samples per group). Data are mean ± s.e.m. *P* values are determined by paired two-sided *t*-tests with Welch’s correction.
